# Catalyst-Free Three-Component Synthesis of 3-Aminoalkyl Chromones Using Rongalite as a C1 Synthon

**DOI:** 10.3390/molecules31091432

**Published:** 2026-04-26

**Authors:** Juanjuan Gao, Xinlei Fu, Ruoying Hu, Jinhua Wan, Kai Yang, Zhaowen Liu, Zhiqin Huang

**Affiliations:** Jiangxi Province Key Laboratory of Pharmacology of Traditional Chinese Medicine, School of Pharmacy, Gannan Medical University, Ganzhou 341000, China; gaoya0758@163.com (J.G.); fxl05020502@163.com (X.F.); 18270932058@163.com (R.H.); 13007261183@163.com (J.W.); kai_yangyang@126.com (K.Y.)

**Keywords:** chromones, *o*-hydroxyaryl enaminones, rongalite, aromatic amines

## Abstract

Herein, a facile and efficient strategy for one-pot synthesis of 3-aminoalkyl chromones from *o*-hydroxyaryl enaminones and aromatic amines using inexpensive rongalite as a C1 source has been developed. This three-component protocol enables domino aminomethylation and annulation of *o*-hydroxyphenyl enaminones without the need for transition metals or oxidants, featuring simple operation, a broad substrate scope, and readily available raw materials, providing a series of 3-aminomethyl chromones in moderate-to-good yields.

## 1. Introduction

**Figure 1:**
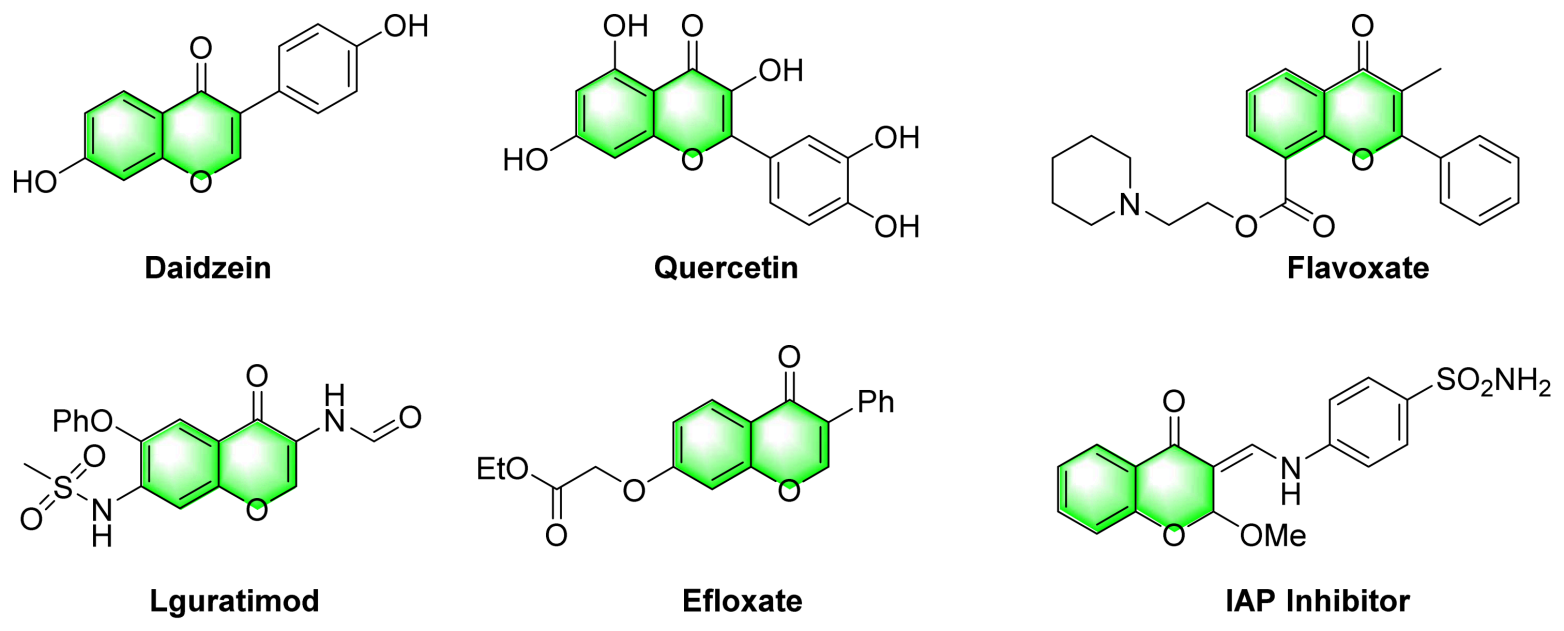
Natural products and bioactive molecules containing a chromone skeleton.

Given that chromone skeletons are widely present in natural products, pharmaceuticals, and various compounds with significant application value, they have been introduced as a pharmacophore into many bioactive molecules for development (Figure 1) [1,2,3,4,5,6,7,8]. Consequently, the synthesis of chromones has long been a continuously studied research topic in the field of organic synthesis [9,10,11]. In its early stages, the direct functionalization of readily accessible chromones and structurally analogous natural compounds (e.g., flavonoids) was the primary strategy for preparing chromone derivatives [12,13,14]. Over the past decade, significant progress has been made in chromone cyclization methodologies, enabling the use of more flexible acyclic starting materials and leveraging diverse transformation pathways for the construction of chromone skeletons [15,16,17,18]. Owing to its high reliability and versatility, the domino C-H functionalization/chromone cyclization of *o*-hydroxyphenyl enaminones stands out as one of the most successful protocols in this field, having been widely applied to the synthesis of chromone derivatives featuring highly diverse substituents at the C2 or C3 position [19,20,21,22,23,24,25,26,27,28].

**Scheme 1:**
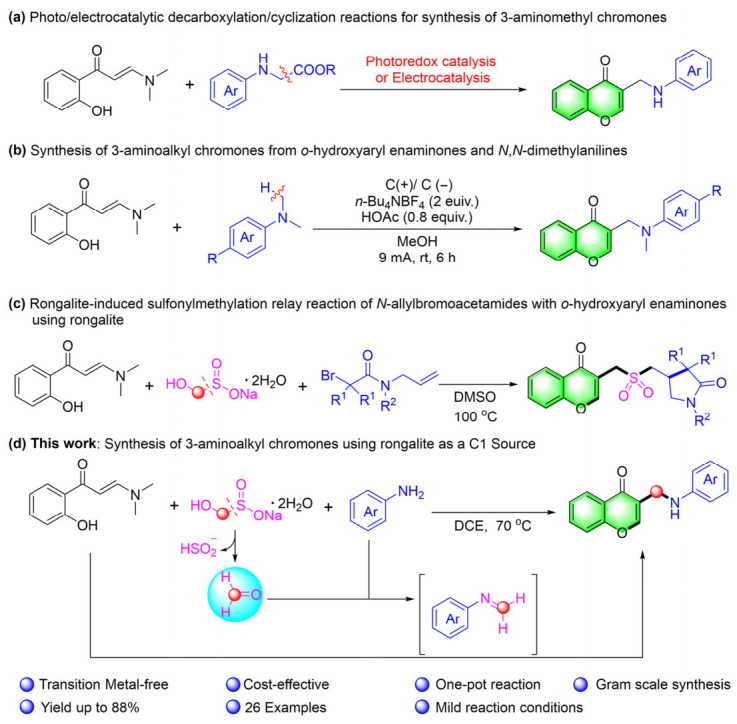
Reported examples of chromone annulation of *o*-hydroxyaryl enaminones.

As a precursor of the chromone skeleton, enaminones can directly introduce various functional groups to construct C3 substituted chromones, such as alkyl [29,30,31,32], aryl [33,34], allyl [35,36], alkenyl [37], acyl [38,39], trifluoromethyl [40], sulfenyl [41,42], selenyl [43,44], amin [45], and phosphoryl [46] groups. It is worth noting that the synthesis of 3-aminomethyl chromones has also garnered significant attention. In 2022, Zhu’s group reported the successful visible-light-driven sequential decarboxylation/cyclization reactions of *o*-hydroxyaryl enaminones with *N*-arylglycine (esters), enabling the efficient synthesis of 3-aminoalkyl chromones [47,48,49]. Cheng’s group developed an innovative electrocatalytic decarboxylation strategy, which also achieved the same transformation in 2024 (Scheme 1a) [50]. He’s group developed an electrochemical catalytic approach for the regioselective synthesis of 3-aminoalkyl chromones via tandem C-H bond alkylation of *o*-hydroxyaryl enaminones and *p*-alkyl-substituted *N*,*N*-dimethylanilines (Scheme 1b) [51].

Rongalite (NaHOCH_2_SO_2_), a versatile and cost-effective reagent (ca. $ 0.03/g), has a wide range of applications in organic synthesis [52]. It often participates in various transformation reactions as a sulfoxylate dianion [53,54,55,56], hydride-free reducing agent [57], single-electron donor [58,59], and C1 synthon [60,61,62,63,64,65,66,67,68]. In 2025, Zhao and coworkers developed an efficient sulfonylmethylation relay reaction of *N*-allylbromoacetamides with *o*-hydroxyaryl enaminones using rongalite as the “SO_2_” and “C1” sources to access lactam−chromone hybrid alkyl−alkyl sulfones. (Scheme 1c) [69]. Inspired by the aforementioned research and our long-standing interest in the derivatization reactions of heterocyclic compounds [70,71,72,73,74], including chromones [75,76], we herein report a one-pot synthesis of 3-aminoalkyl chromones using *o*-hydroxyphenyl enaminones, rongalite, and aromatic amines as substrates. The specific strategy is outlined as follows: amines react with in situ-generated formaldehyde from rongalite to form enamine intermediates, which then undergo a reaction with *o*-hydroxyaryl enaminones to yield 3-aminoalkyl chromones (Scheme 1d).

## 2. Results and Discussion

### 2.1. Optimization of Reaction Conditions

To start this work, *o*-hydroxyaryl enaminone **1a** (0.2 mmol), rongalite **2** (0.3 mmol) and aniline **3a** (0.3 mmol) were selected to identify the proper conditions for the three-component reaction (Table 1). Initially, we screened different reaction solvents at 70 °C for 12 h under additive- and catalyst-free conditions. The yield of the target product **4a** in 1,2-dichloroethane (DCE) (83%) is higher than that in other solvents, such as *N*,*N*-dimethylformamide (DMF), toluene, acetonitrile, 1,4-dioxane, ethanol, ethylene glycol, 1,1,1,3,3,3-hexafluoro-2-propanol (HFIP), water, and dichloromethane (Table 1, entries 1–10). The reaction temperature of 70 °C was identified as the most effective temperature, and neither lower nor higher temperatures increased the yield of the product **4a** (Table 1, entries 10–12). Increasing the amount of aniline **3a** to 2 equivalents can increase the yield of **4a** to 88% (Table 1, entry 10). The established conditions for synthesizing 3-aminoalkyl chromones are as follows: *o*-hydroxyaryl enaminone **1a** (0.2 mmol), rongalite **2** (0.3 mmol), and aniline **3a** (0.4 mmol) in DCE (1 mL) at 70 °C for 12 h.

### 2.2. Scope of Reaction Substrates

**Scheme 2:**
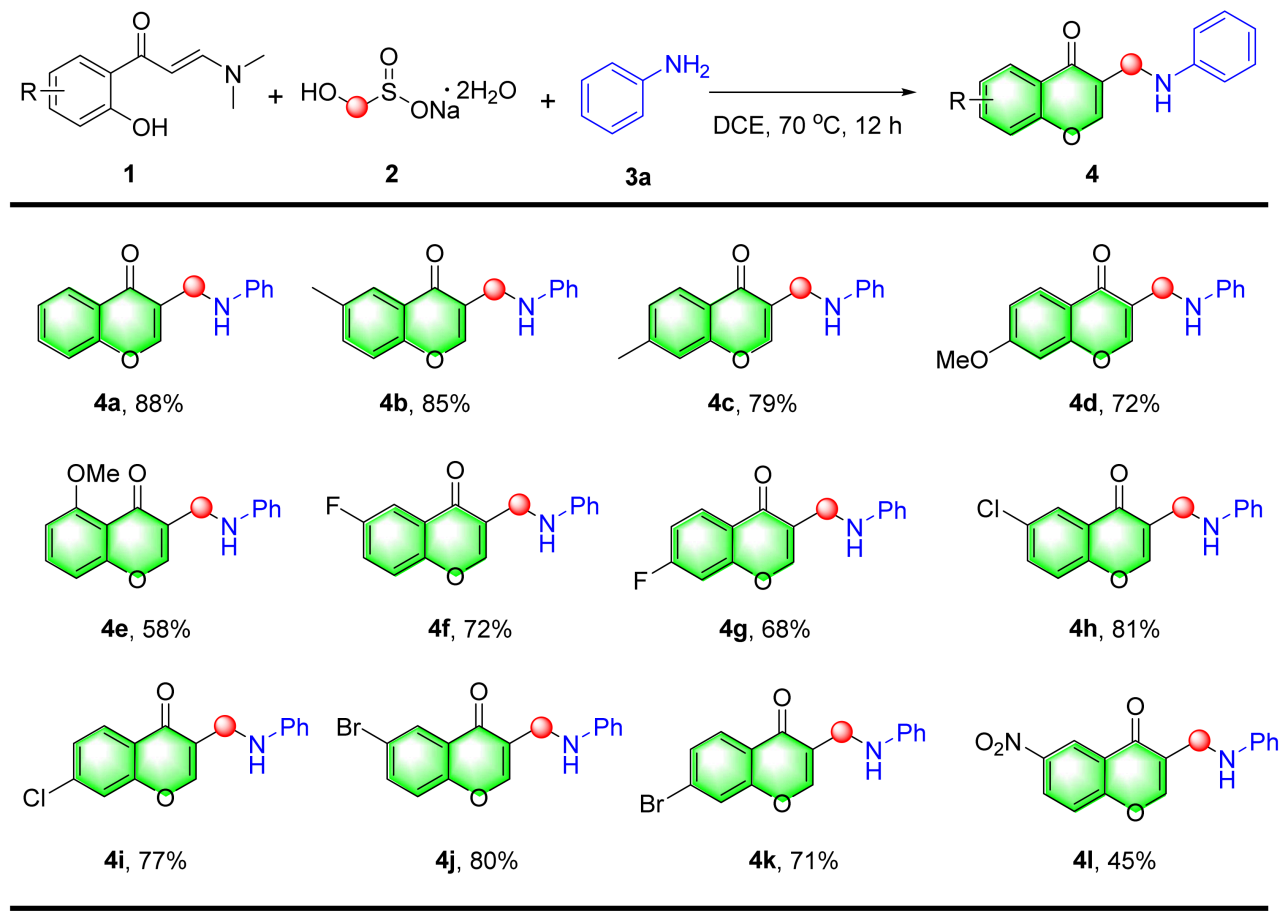
Substrate scope of *o*-hydroxyaryl enaminones **1**. Reaction conditions: **1** (0.2 mmol, 1.0 equiv.), **2** (0.3 mmol, 1.5 equiv.), **3a** (0.4 mmol, 2 equiv.), DCE (1.0 mL), 70 °C, 12 h.

After optimizing the aminomethylation reaction conditions, we concentrated on the scope of the *o*-hydroxyaryl enaminones; the results are shown in Scheme 2. As expected, *o*-hydroxyaryl enaminones with different substituents on the benzene ring, such as the methyl, methoxy, and halo atoms (F, Cl, Br), were tolerated well, producing moderate-to-good yields (58–85%) of the corresponding products **4b**–**4k**. Furthermore, the NO_2_-substituted *o*-hydroxyphenyl enaminone also provided the desired 3-aminoalkyl chromone **4l** in an acceptable yield (45%). This relatively lower yield can be attributed to the strong electron-withdrawing effect of the nitro group, which attenuates the nucleophilic reactivity of the molecule.

**Scheme 3:**
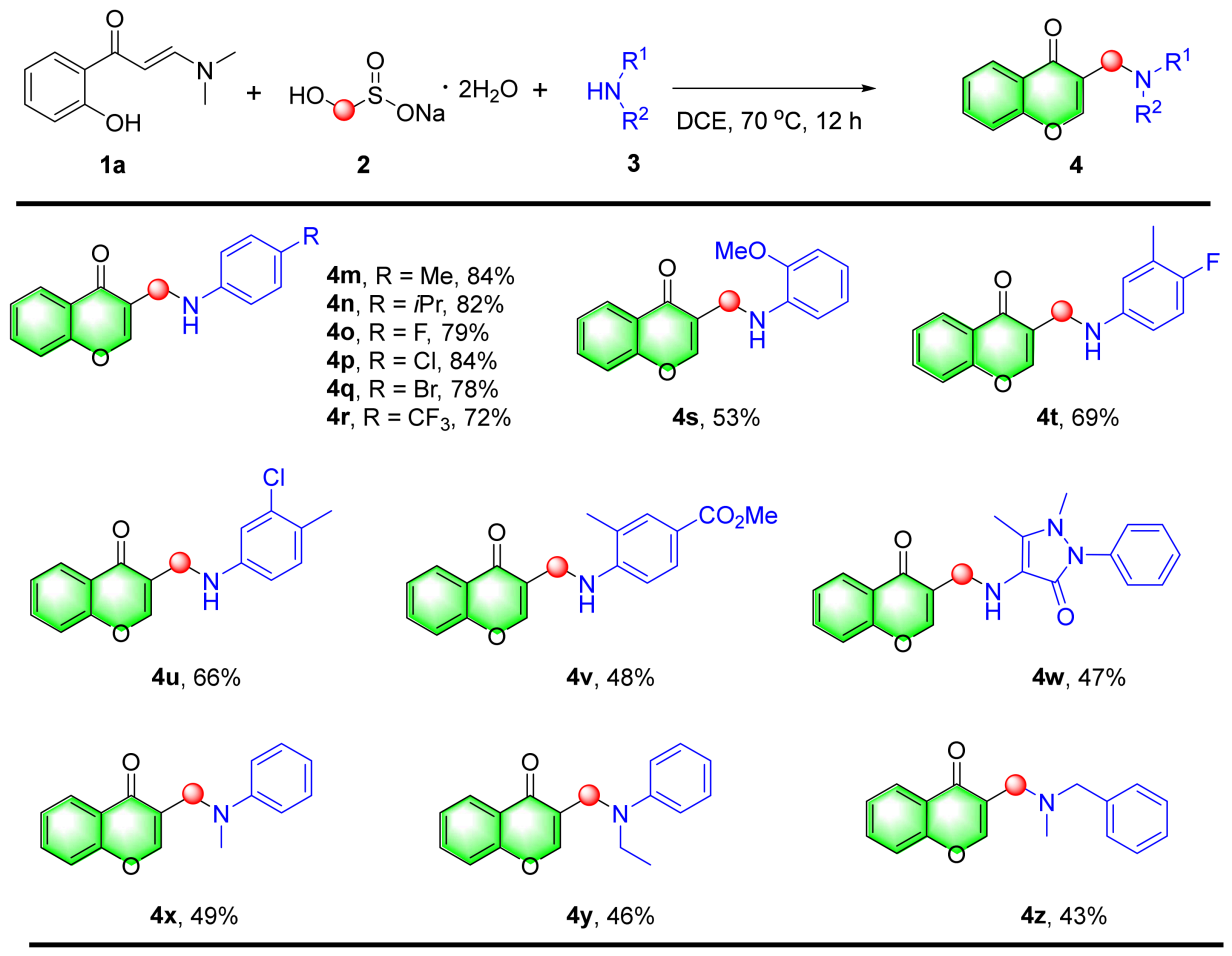
Substrate scope of *o*-hydroxyaryl enaminones **3**. Reaction conditions: **1a** (0.2 mmol, 1.0 equiv.), **2** (0.3 mmol, 1.5 equiv.), **3** (0.4 mmol, 2 equiv.), DCE (1.0 mL), 70 °C, 12 h. Isolated yields based on **1**.

Next, the scope of amine **3** was also investigated. As shown in Scheme 3, 4-substituted anilines bearing electron-donating groups (methyl and isopropyl), halogen groups (fluoro, chloro, bromo), and an electron-withdrawing group (trifluoromethyl) can work smoothly, producing the corresponding 3-aminomethyl chromones **4m**–**4r** in good-to-excellent yields (72–84%). 2-methoxyaniline and disubstituted anilines are also suitable for this conversion, producing the corresponding products **4s**–**4v** in 48–69% yields. As anticipated, 4-aminoantipyrine, an important pharmaceutical intermediate, is also compatible with this protocol, delivering the desired product **4w** in a satisfactory yield (47%). Furthermore, secondary amines such as *N*-methylaniline, *N*-ethylaniline, and *N*-methylbenzylamine can also yield the desired product **4x**–**4z** in acceptable yields (43–49%).

### 2.3. Plausible Reaction Mechanism

**Scheme 4:**
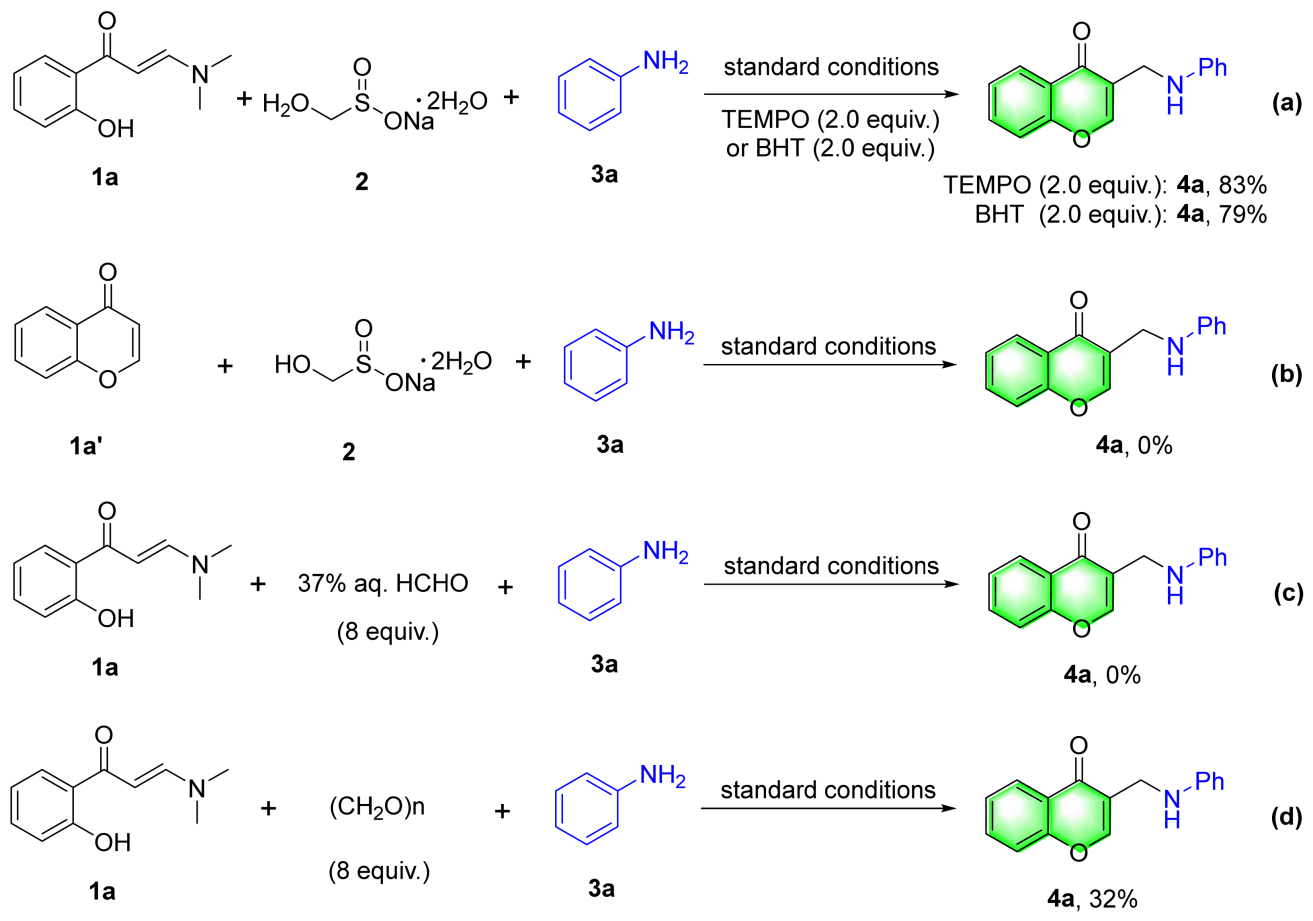
Control experiments.

In order to elucidate the reaction mechanism, control experiments were performed under the standard conditions. When 2.0 equivalents of the radical scavengers TEMPO (2,2,6,6-tetramethyl-1-piperidinyloxy) or BHT (butylated hydroxytoluene) were added to the reaction system, the target compound **4a** was still obtained in isolated yields of 83% and 79%, respectively (Scheme 4a). The results indicate that this transformation may not involve the radical process. Furthermore, chromone **1a’** failed to produce the target compound **4a** under the standard reaction conditions (Scheme 4b), which indicates that the reaction process may not undergo the formation of the intermediate of chromone **1a’**. To further clarify the role of rongalite in this transformation, we first attempted to replace rongalite with aqueous formaldehyde (formalin) under standard reaction conditions, yet the target product **4a** was not detected (Scheme 4c). We hypothesized that this failure might stem from the excessive water content in formalin, which hindered the desired transformation. To verify this hypothesis, we substituted rongalite with paraformaldehyde under identical standard conditions and successfully obtained product **4a** with a 32% yield (Scheme 4d). These results indicate that rongalite probably acts as the formaldehyde source and exhibits superior reactivity compared to paraformaldehyde in this transformation.

**Scheme 5:**
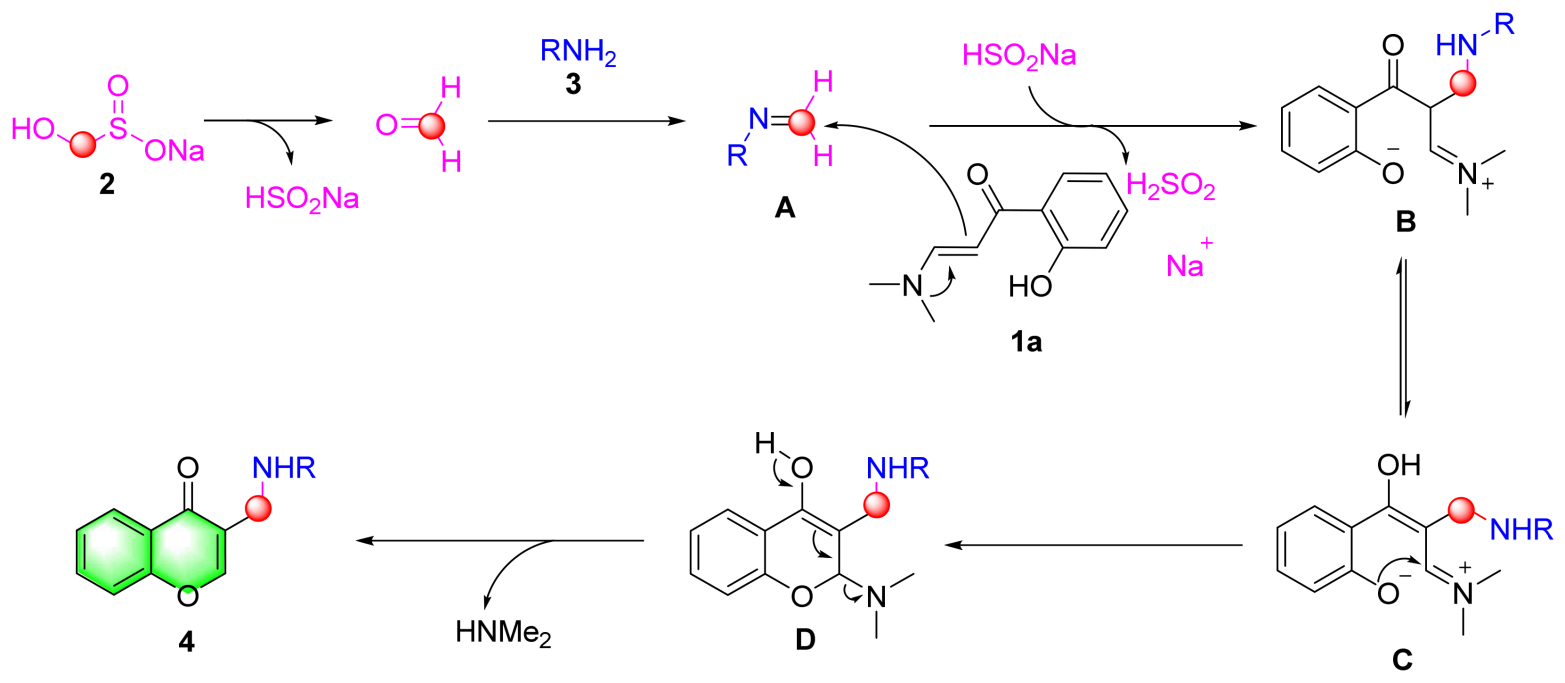
Plausible reaction mechanism.

Based on the aforementioned control experiments and the relevant previous literature [60,61,62,63,64,65,66,67,73,74], a plausible mechanism for the three-component domino reaction is proposed (Scheme 5). Initially, rongalite undergoes dissociation to generate formaldehyde and HSO_2_^−^. Amine **3** reacts with formaldehyde generated in situ from rongalite to produce intermediate **A**, which then undergoes a nucleophilic addition reaction with *o*-hydroxyaryl enaminone **1** to obtain intermediate **B**. Subsequently, tautomerization occurs to form intermediate **C**. Then, intermediate **C** undergoes intramolecular cyclization to form cyclic intermediate **D**. Finally, dimethylamine is eliminated to produce the corresponding compound **4**.

### 2.4. Gram-Scale Synthesis of Compound ***4a*** and Its Synthetic Applications

**Scheme 6:**
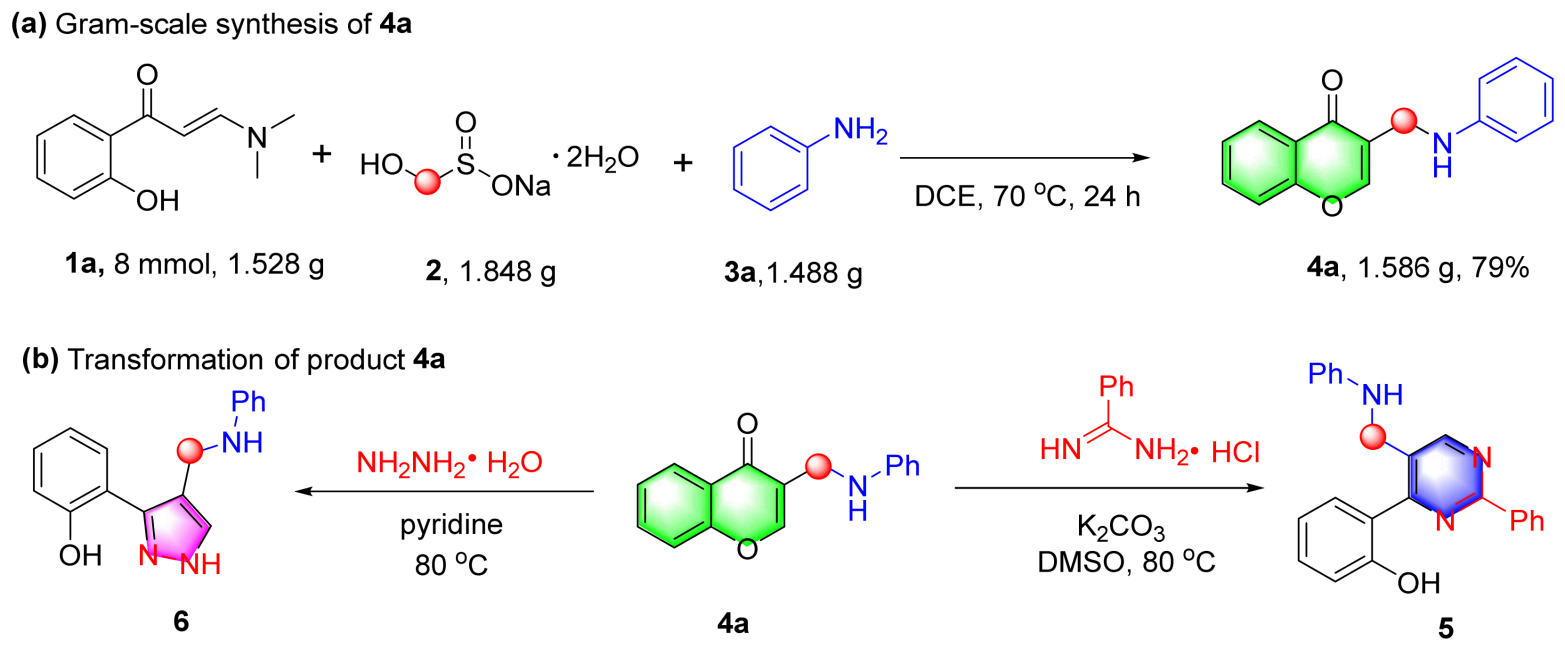
(**a**) Gram-scale synthesis of compound **4a**; (**b**) Synthetic applications of **4a**.

To demonstrate the synthetic scalability and versatility of our developed protocol, a gram-scale experiment was carried out using 8 mmol *o*-hydroxyaryl enaminone **1a** (1.068 g), 12 mmol rongalite **2** (1.848 g), and 16 mmol aniline **3a** (1.488 g). The target product **4a** was obtained with an isolated yield of 79% when the reaction time was extended to 24 h (Scheme 6a). After successfully developing a facile and efficient method for synthesizing 3-aminoalkyl chromones, our group further explored the derivatization potential applications of these compounds (Scheme 6b). As anticipated, compound **4a** reacted with benzamidine hydrochloride to afford pyrimidine derivative **5** at a 86% yield. In addition, pyrazole derivative **6** was obtained with a yield of 65% by reacting **4a** with hydrazine hydrate.

## 3. Materials and Methods

### 3.1. General Information

Melting point determination (m.p.) was performed on a Büchi Melting Point B-545 instrument (Büchi, Flawil, Switzerland) without correction. ^1^H, ^13^C and ^19^F NMR spectra were collected on a BRUKER DRX-4O0 spectrometer (Bruker, Ettlingen, Germany) in CDCl_3_ using tetramethylsilane (TMS) as an internal standard. High-resolution mass spectra (HRMSs) were obtained with a LCMS-IT-TOF mass spectrometer (Waters Corporation, Milford, MA, USA). TLC was performed by using commercially prepared 100–400 mesh silica gel plates (GF254) and visualization was detected at 254 or 365 nm. All reagents and solvents were purchased from commercial sources and used without further purification. (2*E*)-3-(dimethylamino)-1-(2-hydroxyphenyl)prop-2-en-1-ones **1** was synthesized from 2′-hydroxyacetophenones and *N*,*N*-dimethylformamide dimethyl acetal (see Supplementary Materials for details) [75,76].

### 3.2. Experimental Procedure for Compounds ***4***

In a 25 mL oven-dried round-bottom flask, enaminone **1** (0.2 mmol, 1 equiv.), rongalite **2** (0.3 mmol, 1.5 equiv.) and amine **3** (0.4 mmol, 2.0 equiv.) were dissolved in DCE and stirred for 12 h at 70 °C. After the reaction was complete, the solvent was evaporated, and the residue was dissolved in ethyl acetate and washed with brine. The organic layer was dried over anhydrous sodium sulfate, concentrated, and the crude mixture was purified using silica gel column chromatography with ethyl acetate and petroleum ether as the eluent to isolate the product **4**.

### 3.3. Experimental Procedure for Compound ***5***

3-((Phenylamino)methyl)-4*H*-chromen-4-one **4a** (0.2 mmol, 50.3 mg), benzamidine hydrochloride (0.4 mmol, 62.4 mg, 2.0 equiv.), and K_2_CO_3_ (0.4 mmol, 2.0 equiv.) in DMSO (1.0 mL) were stirred in a ground-glass test tube at 80 °C for 4 h. After completion of the reaction, the mixture was extracted with ethyl acetate and aqueous ammonium chloride solution, dried over anhydrous Na_2_SO_4_, and concentrated under reduced pressure. The crude product was purified by flash column chromatography using a mixed eluent of petroleum ether/ethyl acetate (*v*/*v* = 1:1), producing 60.7 mg of pure, yellow solid product **5** with a yield of 86%.

### 3.4. Experimental Procedure for Compound ***6***

To a reaction tube fitted with a stirrer, 3-((Phenylamino)methyl)-4*H*-chromen-4-one **4a** (0.2 mmol, 50.3 mg), hydrazine hydrate 80% (1.0 mmol, 10.0 equiv.), and pyridine (1.0 mL) were added sequentially. Subsequently, the reaction mixture was heated to 80 °C and stirred at this temperature for 8 h. After completion of the reaction, the mixture was extracted with ethyl acetate and aqueous ammonium chloride solution, dried over anhydrous Na_2_SO_4_, and concentrated under reduced pressure. The crude product was then separated and purified by flash column chromatography using a mixed eluent of petroleum ether/ethyl acetate (*v*/*v* = 2:1), yielding 34.5 mg of a white solid product **6** with a yield of 65%.

### 3.5. Characterization Data for All Products ***4a***–***4z***, ***5*** and ***6***

**3-((Phenylamino)methyl)-4*H*-chromen-4-one** (**4a**): Yellow solid, 88% yield; m.p. 125–128 °C (m.p. 145–149 °C [48]); ^1^H NMR (400 MHz, CDCl_3_), *δ* 8.15 (d, *J* = 7.6 Hz, 1H, ArH), 7.83 (s, 1H, ArH), 7.59–7.55 (m, 1H, ArH), 7.35–7.30 (m, 2H, ArH), 7.11–7.07 (m, 2H, ArH), 6.66–6.62 (m, 1H, Ar), 6.57 (d, *J* = 8.4 Hz, 2H, ArH), 4.20 (s, 2H, CH_2_) ppm; ^13^C NMR (100 MHz, CDCl_3_), *δ* 178.0, 156.6, 153.2, 147.5, 133.7, 129.3, 125.7, 125.2, 123.9, 121.3, 118.2, 118.1, 113.4, 40.5 ppm.

**6-Methyl-3-((phenylamino)methyl)-4*H*-chromen-4-one** (**4b**): Yellow solid, 85% yield; m.p. 121–123 °C (m.p. 115–116 °C [50]); ^1^H NMR (400 MHz, CDCl_3_), *δ* 8.00 (s, 1H, ArH), 7.89 (s, 1H, ArH), 7.46 (dd, *J* = 8.0, 1.6 Hz, 1H, ArH), 7.31 (d, *J* = 8.0 Hz, 1H, ArH), 7.18–7.14 (m, 2H, ArH), 6.74–6.69 (m, 1H, ArH), 6.64 (d, *J* = 7.6 Hz, 2H, ArH), 4.26 (s, 2H, CH_2_), 2.45 (s, 3H, CH_3_) ppm; ^13^C NMR (100 MHz, CDCl_3_), *δ* 178.1, 154.9, 153.1, 147.5, 135.1, 135.0, 129.3, 124.9, 123.5, 121.0, 118.1, 117.9, 113.5, 40.5, 20.9 ppm.

**7-Methyl-3-((phenylamino)methyl)-4*H*-chromen-4-one** (**4c**): Yellow solid, 79% yield; m.p. 117–119 °C; ^1^H NMR (400 MHz, CDCl_3_), *δ* 8.10 (d, *J* = 8.4 Hz, 1H, ArH), 7.86 (s, 1H, ArH), 7.22–7.15 (m, 4H, ArH), 6.73–6.70 (m, 1H, ArH), 6.64 (d, *J* = 7.6 Hz, 2H, ArH), 4.42 (br, 1H, NH), 4.26 (s, 2H, CH_2_), 2.47 (s, 3H, CH_3_) ppm; ^13^C NMR (100 MHz, CDCl_3_), *δ* 177.9, 156.7, 152.9, 147.5, 145.1, 129.3, 126.7, 125.4, 121.6, 121.1, 118.0, 117.8, 113.4, 40.5, 21.8 ppm; ESI-HRMS, m/z: Calcd for C_17_H_16_NO_2_^+^ [M + H]^+^: 266.1176, found: 266.1182.

**7-Methoxy-3-((phenylamino)methyl)-4*H*-chromen-4-one** (**4d**): Yellow solid, 72% yield; m.p. 148–150 °C; ^1^H NMR (400 MHz, CDCl_3_), *δ* 8.12 (d, *J* = 8.8 Hz, 1H, ArH), 7.83 (s, 1H, ArH), 7.18–7.14 (m, 2H, ArH), 6.96 (dd, *J* = 8.8, 2.4 Hz, 1H, ArH), 6.79 (d, *J* = 2.4 Hz, 1H, ArH), 6.73–6.70 (m, 1H, ArH), 6.64 (d, *J* = 7.6 Hz, 2H, ArH), 4.24 (s, 2H, CH_2_), 3.89 (s, 3H, OCH_3_) ppm; ^13^C NMR (100 MHz, CDCl_3_), *δ* 177.3, 164.1, 158.3, 152.6, 147.5, 129.3, 127.0, 121.0, 118.0, 114.7, 113.4, 112.8, 100.1, 55.8, 40.5 ppm; ESI-HRMS, m/z: Calcd for C_17_H_16_NO_3_^+^ [M + H]^+^: 282.1125, found: 282.1136.

**5-Methoxy-3-((phenylamino)methyl)-4*H*-chromen-4-one** (**4e**): Yellow solid, 58% yield; m.p. 125–127 °C; ^1^H NMR (400 MHz, CDCl_3_), *δ* 7.70 (s, 1H, ArH), 7.54–7.49 (m, 1H, ArH), 7.18–7.13 (m, 2H, ArH), 6.97 (d, *J* = 8.4 Hz, 1H, ArH), 6.78 (d, *J* = 8.0 Hz, 1H, ArH), 6.72–6.68 (m, 1H, ArH), 6.63 (d, *J* = 7.6 Hz, 2H, ArH), 4.46 (br, 1H, NH), 4.20 (s, 2H, CH_2_), 3.98 (s, 3H, OCH_3_) ppm; ^13^C NMR (100 MHz, CDCl_3_), *δ* 177.9, 159.9, 158.6, 151.1, 147.6, 133.8, 129.3, 122.2, 117.9, 114.6, 113.5, 110.2, 106.1, 56.4, 40.5 ppm; ESI-HRMS, m/z: Calcd for C_17_H_16_NO_3_^+^ [M + H]^+^: 282.1125, found: 282.1136.

**6-Fluoro-3-((phenylamino)methyl)-4*H*-chromen-4-one** (**4f**): Yellow solid, 72% yield; m.p. 119–121 °C (m.p. 118–119 °C [50]); ^1^H NMR (400 MHz, CDCl_3_), *δ* 7.92 (s, 1H, ArH), 7.86 (dd, *J* = 8.0, 2.8 Hz, 1H, ArH), 7.46–7.42 (m, 1H, ArH), 7.41–7.37 (m, 1H, ArH), 7.19–7.15 (m, 2H, ArH), 6.75–6.70 (m, 1H, ArH), 6.64 (d, *J* = 8.4 Hz, 2H, ArH), 4.34 (br, 1H, NH), 4.28 (s, 2H, CH_2_) ppm; ^19^F NMR (376 MHz, CDCl_3_), *δ* −115.03 ppm; ^13^C NMR (100 MHz, CDCl_3_), *δ* 177.2, 159.5 (d, *J* = 245.3 Hz), 153.4, 152.8, 147.3, 129.3, 124.9 (d, *J* = 7.3 Hz), 122.0 (d, *J* = 25.5 Hz), 120.7, 120.3 (d, *J* = 8.0 Hz), 118.1, 113.3, 110.4 (d, *J* = 23.5 Hz), 40.3 ppm.

**7-Fluoro-3-((phenylamino)methyl)-4*H*-chromen-4-one** (**4g**): Yellow solid, 68% yield; m.p. 131–133 °C; ^1^H NMR (400 MHz, CDCl_3_), *δ* 8.25–8.21 (m, 1H, ArH), 7.87 (s, 1H, ArH), 7.19–7.09, (m, 4H, ArH), 6.75–6.70 (m, 1H, ArH), 6.63 (d, *J* = 8.0 Hz, 2H, ArH), 4.34 (br, 1H, NH), 4.34 (s, 2H, CH_2_) ppm; ^19^F NMR (376 MHz, CDCl_3_), *δ* −102.67 ppm; ^13^C NMR (100 MHz, CDCl_3_), *δ* 177.1, 165.6 (d, *J* = 253.7 Hz), 157.5 (d, *J* = 13.4 Hz), 153.2, 147.3, 129.4, 128.2 (d, *J* = 10.6 Hz), 121.5, 120.7 (d, *J* = 2.4 Hz), 118.1, 114.0 (d, *J* = 22.7 Hz), 113.9, 113.3, 104.7 (d, *J* = 25.1 Hz), 40.3 ppm; ESI-HRMS, m/z: Calcd for C_16_H_13_FNO_2_^+^ [M + H]^+^: 270.0925, found: 270.0928.

**6-Chloro-3-((phenylamino)methyl)-4*H*-chromen-4-one** (**4h**): Yellow solid, 81% yield; m.p. 115–117 °C (m.p. 102–103 °C [50]); ^1^H NMR (400 MHz, CDCl_3_), *δ* 8.18 (d, *J* = 2.0 Hz, 1H, ArH), 7.89 (s, 1H, ArH), 7.58 (dd, *J* = 8.8, 2.0 Hz, 1H, ArH), 7.38 (d, *J* = 8.8 Hz, 1H, ArH), 7.19–7.14 (m, 2H, ArH), 6.74–6.70 (m, 1H, ArH), 6.63 (d, *J* = 8.0 Hz, 2H, ArH), 4.32 (br, 1H, NH), 4.27 (s, 2H, CH_2_) ppm; ^13^C NMR (100 MHz, CDCl_3_), *δ* 176.8, 154.9, 153.3, 147.3, 133.9, 131.1, 129.4, 125.1, 124.7, 121.4, 119.9, 118.2, 113.4, 40.3 ppm.

**7-Chloro-3-((phenylamino)methyl)-4*H*-chromen-4-one** (**4i**): Yellow solid, 77% yield; m.p. 132–134 °C; ^1^H NMR (400 MHz, CDCl_3_), *δ* 8.14 (d, *J* = 8.4 Hz, 1H, ArH), 7.86 (s, 1H, ArH), 7.43 (s, 1H, ArH), 7.35 (d, *J* = 8.4 Hz, 1H, ArH), 7.19–7.14 (m, 2H, ArH), 6.75–6.70 (m, 1H, ArH), 6.63 (d, *J* = 8.4 Hz, 2H, ArH), 4.26 (s, 2H, CH_2_) ppm; ^13^C NMR (100 MHz, CDCl_3_), *δ* 177.2, 156.6, 153.2, 147.3, 139.8, 129.4, 127.1, 126.1, 122.3, 121.7, 118.2, 115.1, 113.3, 40.3 ppm; ESI-HRMS, m/z: Calcd for C_16_H_13_ClNO_2_^+^ [M + H]^+^: 286.0629, found: 286.0652.

**6-Bromo-3-((phenylamino)methyl)-4*H*-chromen-4-one** (**4j**): Yellow solid, 80% yield; m.p. 107–109 °C (m.p. 92–93 °C [50]); ^1^H NMR (400 MHz, CDCl_3_), *δ* 8.33 (dd, *J* = 2.4 Hz, 1H, ArH), 7.89 (s, 1H, ArH), 7.70 (dd, *J* = 8.8, 2.4 Hz, 1H, ArH), 7.30 (d, *J* = 9.2 Hz, 1H, ArH), 7.18–7.14 (m, 2H, ArH), 6.74–6.70 (m, 1H, ArH), 6.62 (d, *J* = 8.4 Hz, 2H, ArH), 4.32 (br, 1H, NH), 4.26 (s, 2H, CH_2_) ppm; ^13^C NMR (100 MHz, CDCl_3_), *δ* 176.7, 155.3, 153.3, 147.3, 136.7, 129.4, 128.3, 125.1, 121.6, 120.2, 118.6, 118.2, 113.4, 40.3 ppm.

**7-Bromo-3-((phenylamino)methyl)-4*H*-chromen-4-one** (**4k**): Yellow solid, 71% yield; m.p. 139–141 °C; ^1^H NMR (400 MHz, CDCl_3_), *δ* 8.08 (d, *J* = 8.4 Hz, 1H, ArH), 7.87 (s, 1H, ArH), 7.63 (d, *J* = 1.6 Hz, 1H, ArH), 7.52 (dd, *J* = 8.4, 1.6 Hz, 1H, ArH), 7.20–7.15 (m, 2H, ArH), 6.76–6.71 (m, 1H, ArH), 6.63 (d, *J* = 8.0 Hz, 2H, ArH), 4.32 (br, 1H, NH), 4.27 (s, 2H, CH_2_) ppm; ^13^C NMR (100 MHz, CDCl_3_), *δ* 177.3, 156.6, 153.1, 147.3, 129.4, 129.2, 128.8, 128.0, 127.1, 121.3, 118.2, 113.3, 112.8, 40.3 ppm; ESI-HRMS, m/z: Calcd for C16H13BrNO2+ [M + H]+: 330.0124, found: 330.0144.

**6-Nitro-3-((phenylamino)methyl)-4*H*-chromen-4-one** (**4l**): Yellow solid, 45% yield; m.p. 118–120 °C (m.p. 115–116 °C [77]); ^1^H NMR (400 MHz, CDCl_3_), *δ* 9.10 (d, *J* = 2.4 Hz, 1H, ArH), 8.49 (dd, *J* = 9.2, 2.4 Hz, 1H, ArH), 7.95 (s, 1H, ArH), 7.58 (d, *J* = 9.2 Hz, 1H, ArH), 7.18 (t, *J* = 8.0 Hz, 2H, ArH), 6.77–6.73 (m, 1H, ArH), 6.63 (d, *J* = 8.0 Hz, 2H, ArH), 4.32 (s, 2H, CH_2_) ppm; ^13^C NMR (100 MHz, CDCl_3_), *δ* 176.5, 159.3, 153.4, 147.0, 144.7, 129.4, 128.0, 123.8, 122.5, 122.3, 120.0, 118.4, 113.3, 40.1 ppm.

**3-((*p*-Tolylamino)methyl)-4*H*-chromen-4-one** (**4m**): Yellow solid, 84% yield; m.p. 128–130 °C (m.p. 142–143 °C [50]); ^1^H NMR (400 MHz, CDCl_3_), *δ* 8.23 (d, *J* = 8.0 Hz, 1H, ArH), 7.91 (s, 1H, ArH), 7.68–7.63 (m, 1H, ArH), 7.44–7.38 (m, 2H, ArH), 6.98 (d, *J* = 8.0 Hz, 2H, ArH), 6.57 (d, *J* = 8.0 Hz, 2H, ArH), 4.34 (br, 1H, NH), 4.25 (s, 2H, CH_2_), 2.22 (s, 3H, CH_3_) ppm; ^13^C NMR (100 MHz, CDCl_3_), *δ* 178.1, 156.6, 153.2, 145.1, 133.7, 129.8, 127.3, 125.7, 125.1, 123.8, 121.4, 118.2, 113.6, 40.8, 20.4 ppm; ESI-HRMS, m/z: Calcd for C_17_H_16_NO_2_^+^ [M + H]^+^: 266.1176, found: 266.1171.

**3-(((4-Isopropylphenyl)amino)methyl)-4*H*-chromen-4-one** (**4n**): Yellow solid, 82% yield; m.p. 99–101 °C; ^1^H NMR (400 MHz, CDCl_3_), *δ* 8.23 (dd, *J* = 8.0, 0.8 Hz, 1H, ArH), 7.92 (s, 1H, ArH), 7.68–7.64 (m, 1H, ArH), 7.44–7.39 (m, 2H, ArH), 7.04 (d, *J* = 8.0 Hz, 2H, ArH), 6.60 (d, *J* = 8.4 Hz, 2H, ArH), 4.26 (s, 2H, CH_2_), 1.19 (d, *J* = 7.2 Hz, 6H, 2CH_3_) ppm; ^13^C NMR (100 MHz, CDCl_3_), *δ* 178.1, 156.6, 153.2, 145.4, 138.6, 133.7, 127.2, 125.7, 125.1, 123.9, 121.4, 118.2, 113.4, 40.7, 33.2, 24.2 ppm; ESI-HRMS, m/z: Calcd for C_19_H_20_NO_2_^+^ [M + H]^+^: 294.1489, found: 294.1498.

**3-(((4-Fluorophenyl)amino)methyl)-4*H*-chromen-4-one** (**4o**): Yellow solid, 79% yield; m.p. 142–144 °C (m.p. 150–151 °C [77]); ^1^H NMR (400 MHz, CDCl_3_), *δ* 8.23 (d, *J* = 7.6 Hz 1H, ArH), 7.91 (s, 1H, ArH), 7.69–7.65 (m, 1H, ArH), 7.45–7.40 (m, 2H, ArH), 6.90–6.85 (m, 2H, ArH), 6.60–6.57 (m, 2H, ArH), 4.22 (s, 2H, CH_2_) ppm; ^19^F NMR (376 MHz, CDCl_3_), *δ*, ppm: −127.2; ^13^C NMR (100 MHz, CDCl_3_), *δ* 178.0, 156.5, 156.2 (d, *J* = 234.3 Hz), 153.2, 143.8 (d, *J* = 2.0 Hz), 133.8, 125.7, 125.2, 123.8, 121.1, 118.2, 115.8 (d, *J* = 22.1 Hz), 114.4 (d, *J* = 7.5 Hz), 41.3 ppm.

**3-(((4-Chlorophenyl)amino)methyl)-4*H*-chromen-4-one** (**4p**): Yellow solid, 84% yield; m.p. 131–133 °C (m.p. 124–128 °C [47]); ^1^H NMR (400 MHz, CDCl_3_), *δ* 8.15 (d, *J* = 8.0 Hz, 1H, ArH), 7.82 (s, 1H, ArH), 7.61–7.58 (m, 1H, ArH), 7.45–7.32 (m, 3H, ArH), 7.03 (d, *J* = 8.8 Hz, 2H, ArH), 6.49 (d, *J* = 8.8 Hz, 2H, ArH), 4.16 (s, 2H, CH_2_) ppm; ^13^C NMR (100 MHz, CDCl_3_), *δ* 178.0, 156.6, 153.1, 146.1, 133.8, 129.2, 125.7, 125.3, 123.8, 122.7, 120.9, 118.2, 114.6, 40.7 ppm.

**3-(((4-Bromophenyl)amino)methyl)-4*H*-chromen-4-one** (**4q**): Yellow solid, 78% yield; m.p. 125–127 °C (m.p. 121–126 °C [47]); ^1^H NMR (400 MHz, CDCl_3_), *δ* 8.15 (d, *J* = 8.0 Hz 1H, ArH), 7.82 (s, 1H, ArH), 7.62–7.58 (m, 1H, ArH), 7.40–7.32 (m, 3H, ArH), 7.19–7.15 (m, 2H, ArH), 6.45 (d, *J* = 8.4 Hz, 2H, ArH), 4.16 (s, 2H, CH_2_) ppm; ^13^C NMR (100 MHz, CDCl_3_), *δ* 178.0, 156.6, 153.1, 146.5, 133.9, 132.0, 125.7, 125.3, 123.8, 120.8, 118.2, 115.0, 109.7, 40.5 ppm; ESI-HRMS, m/z: Calcd for C_16_H_13_BrNO_2_^+^ [M + H]^+^: 330.0124, found: 330.0093.

**3-(((4-(Trifluoromethyl)phenyl)amino)methyl)-4*H*-chromen-4-one** (**4r**): White solid, 72% yield; m.p. 152–154 °C (mp 146–147 °C [77]); ^1^H NMR (400 MHz, CDCl_3_), *δ* 8.16 (dd, *J* = 8.0, 1.6 Hz 1H, ArH), 7.84 (s, 1H, ArH), 7.63–7.59 (m, 1H, ArH), 7.39–7.32 (m, 4H, ArH), 7.19 (s, 1H, ArH), 6.59 (d, *J* = 8.4 Hz, 2H, ArH), 4.23 (s, 2H, CH_2_) ppm; ^19^F NMR (376 MHz, CDCl_3_), *δ* −61.16 ppm:; ^13^C NMR (100 MHz, CDCl_3_), *δ* 177.9, 156.6, 153.1, 150.0, 133.9, 126.7 (q, *J* = 3.2 Hz), 125.5 (q, *J* = 34.0 Hz), 124.5, 124.2 (q, *J* = 272.2 Hz), 123.8, 120.6, 119.1, 118.2, 112.5, 37.1 ppm.

**3-(((2-Methoxyphenyl)amino)methyl)-4*H*-chromen-4-one** (**4s**): Yellow solid, 53% yield; m.p. 133–135 °C (mp 130–131 °C [77]); ^1^H NMR (400 MHz, CDCl_3_), *δ* 8.16 (dd, *J* = 8.0, 1.2 Hz 1H, ArH), 7.80 (s, 1H, ArH), 7.58–7.54 (m, 1H, ArH), 7.34–7.29 (m, 2H, ArH), 6.77–6.73 (m, 1H, ArH), 6.70 (d, *J* = 7.6 Hz, 1H, ArH), 6.62–6.58 (m, 1H, ArH), 6.51 (d, *J* = 7.6 Hz, 1H, ArH), 4.24 (s, 2H, CH_2_), 3.77 (s, 3H, CH_3_) ppm; ^13^C NMR (100 MHz, CDCl_3_), *δ* 177.9, 156.6, 153.2, 147.2, 137.4, 133.6, 125.7, 125.1, 123.8, 121.4, 121.3, 118.2, 117.2, 110.2, 109.6, 55.5, 39.8 ppm.

**3-(((4-Fluoro-3-methylphenyl)amino)methyl)-4*H*-chromen-4-one** (**4t**): Yellow solid, 69% yield; m.p. 129–131 °C; ^1^H NMR (400 MHz, CDCl_3_), *δ* 8.16 (d, *J* = 8.0 Hz 1H, ArH), 7.81 (s, 1H, ArH), 7.61–7.57 (m, 1H, ArH), 7.37–7.32 (m, 2H, ArH), 6.73–6.69 (m, 2H, ArH), 6.47–6.44 (m, 1H, ArH), 5.22 (d, *J* = 2.4 Hz, 1H, ArH), 4.20 (s, 2H, CH_2_), 2.09 (s, 3H, CH_3_) ppm; ^19^F NMR (376 MHz, CDCl_3_), *δ*, ppm: −127.8; ^13^C NMR (100 MHz, CDCl_3_), *δ* 178.1, 156.5, 155.7 (d, *J* = 234 Hz), 153.2, 141.7 (d, *J* = 2 Hz), 133.8, 125.7, 125.2, 124.8 (d, *J* = 7 Hz), 123.8, 121.1, 118.2, 117.1 (d, *J* = 23 Hz), 112.8 (d, *J* = 22 Hz), 111.2 (d, *J* = 8 Hz), 41.1, 17.6 ppm; ESI-HRMS, m/z: Calcd for C_17_H_15_FNO_2_^+^ [M + H]^+^: 284.1081, found: 284.1071.

**3-(((3-Chloro-4-methylphenyl)amino)methyl)-4*H*-chromen-4-one** (**4u**): Yellow solid, 66% yield; m.p. 133–135 °C (mp 112–114 °C [29]); ^1^H NMR (400 MHz, CDCl_3_), *δ* 8.15 (d, *J* = 8.0 Hz 1H, ArH), 7.83 (s, 1H, ArH), 7.60–7.57 (m, 1H, ArH), 7.37–7.32 (m, 2H, ArH), 6.91 (d, *J* = 8.4 Hz, 1H, ArH), 6.59 (s, 1H, ArH), 6.39 (dd, *J* = 8.4, 0.8 Hz 1H, ArH), 4.15 (s, 2H, CH_2_), 2.16 (s, 3H, CH_3_) ppm; ^13^C NMR (100 MHz, CDCl_3_), *δ* 178.0, 156.6, 153.1, 146.6, 134.9, 133.8, 131.4, 125.7, 125.2, 125.0, 123.8, 120.9, 118.2, 113.8, 112.3, 40.8, 18.9 ppm; ESI-HRMS, m/z: Calcd for C_17_H_15_ClNO_2_^+^ [M + H]^+^: 300.0786, found: 300.0764.

**Methyl 3-methyl-4-(((4-oxo-4*H*-chromen-3-yl)methyl)amino)benzoate** (**4v**): White solid, 48% yield; m.p. 139–141 °C; ^1^H NMR (400 MHz, CDCl_3_), *δ* 8.22 (d, *J* = 7.2 Hz 1H, ArH), 7.96 (s, 1H, ArH), 7.68–7.64 (m, 1H, ArH), 7.44–7.40 (m, 2H, ArH), 7.34 (d, *J* = 7.6 Hz, 1H, ArH), 7.31 (s, 1H, ArH), 7.09 (d, *J* = 7.6 Hz 1H, ArH), 4.51 (br, 21H, NH), 4.35 (s, 2H, CH_2_), 3.87 (s, 3H, OCH_3_), 2.20 (s, 3H, CH_3_) ppm; ^13^C NMR (100 MHz, CDCl_3_), *δ* 178.1, 167.6, 156.5, 153.2, 145.5, 133.8, 130.2, 128.9, 128.5, 125.7, 125.2, 123.9, 120.9, 119.0, 118.2, 110.7, 51.9, 40.6, 17.7 ppm; ESI-HRMS, m/z: Calcd for C_19_H_18_NO_4_^+^ [M + H]^+^: 324.1230, found: 324.1261.

**1,5-Dimethyl-4-(((4-oxo-4*H*-chromen-3-yl)methyl)amino)-2-phenyl-1,2-dihydro-3*H*-pyrazol-3-one** (**4w**): Yellow solid, 47% yield; m.p. 134–136 °C; ^1^H NMR (400 MHz, CDCl_3_), *δ* 8.23 (d, *J* = 8.0 Hz 1H, ArH), 7.02 (s, 1H, ArH), 7.68–7.64 (m, 1H, ArH), 7.49–7.38 (m, 6H, ArH), 7.25–7.18 (m, 1H, ArH), 4.25 (s, 2H, CH_2_), 2.83 (s, 3H, CH_3_), 2.12 (s, 3H, CH_3_) ppm; ^13^C NMR (100 MHz, CDCl_3_), *δ* 178.1, 162.2, 156.6, 153.8, 141.6, 135.3, 133.6, 129.0, 126.0, 125.6, 125.1, 123.9, 123.0, 122.3, 120.3, 118.3, 42.8, 37.7, 10.3 ppm; ESI-HRMS, m/z: Calcd for C_21_H_20_N_3_O_3_^+^ [M + H]^+^: 362.1499, found: 362.1518.

**3-((Methyl(phenyl)amino)methyl)-4*H*-chromen-4-one** (**4x**): Yellow solid, 49% yield; m.p. 125–127 °C (mp 131–136 °C [47]); ^1^H NMR (400 MHz, CDCl_3_), *δ* 8.18 (d, *J* = 7.6 Hz, 1H, ArH), 7.62–7.58 (m, 2H, ArH), 7.38–7.33 (m, 2H, ArH), 7.19–7.15 (m, 3H, ArH), 6.68 (m, 3H, ArH), 4.40 (s, 2H, CH_2_), 3.01 (s, 3H, CH_3_) ppm; ^13^C NMR (100 MHz, CDCl_3_), *δ* 178.1, 156.6, 152.8, 133.6, 129.3, 125.6, 125.1, 123.7, 120.1, 118.2, 117.0, 112.3, 49.2, 38.9 ppm.

**3-((Ethyl(phenyl)amino)methyl)-4*H*-chromen-4-one** (**4y**): Yellow solid, 46% yield; m.p. 118–120 °C; ^1^H NMR (400 MHz, CDCl_3_), *δ* 8.19 (d, *J* = 8.0 Hz, 1H, ArH), 7.62–7.57 (m, 2H, ArH), 7.38–7.33 (m, 2H, ArH), 7.19–7.12 (m, 3H, ArH), 6.65–6.61 (m, 3H, ArH), 4.38 (s, 2H, CH_2_), 4.45–4.40 (q, *J* = 6.8 Hz, 2H, CH_2_), 1.17 (t, *J* = 6.8 Hz, 3H, CH_3_) ppm; ^13^C NMR (100 MHz, CDCl_3_), *δ* 178.2, 156.7, 153.0, 147.6, 133.6, 129.4, 125.6, 125.1, 123.6, 120.1, 118.2, 116.5, 112.1, 46.6, 45.5, 12.2 ppm; ESI-HRMS, m/z: Calcd for C_18_H_18_NO_2_^+^ [M + H]^+^: 280.1332, found: 280.1316.

**3-((Benzyl(methyl)amino)methyl)-4*H*-chromen-4-one** (**4z**): Yellow solid, 43% yield; m.p. 135–137 °C; ^1^H NMR (400 MHz, CDCl_3_), *δ* 8.24–8.22 (m, 1H, ArH), 8.07 (s, 1H, ArH), 7.68–7.63 (m, 1H, ArH), 7.47–7.44 (m, 1H, ArH), 7.39–7.36 (m, 3H, ArH), 7.34–7.30 (m, 2H, ArH), 7.25 (d, *J* = 7.2 Hz, 1H, ArH), 3.63 (s, 2H, CH_2_), 3.54 (s, 2H, CH_2_), 2.28 (s, 3H, CH_3_) ppm; ^13^C NMR (100 MHz, CDCl_3_), *δ* 177.7, 156.4, 154.6, 138.8, 133.4, 128.9, 128.3, 127.1, 125.9, 125.0, 123.9, 118.1, 113.0, 62.1, 51.4, 42.4 ppm; ESI-HRMS, m/z: Calcd for C_18_H_18_NO_2_^+^ [M + H]^+^: 280.1332, found: 280.1344.

**2-(2-Phenyl-5-((phenylamino)methyl)pyrimidin-4-yl)phenol** (**5**): Yellow solid, 86% yield; m.p. 168–169 °C (mp 181–182 °C [50]); ^1^H NMR (400 MHz, CDCl_3_), *δ* 8.96 (s, 1H, ArH), 8.38–8.36 (m, 2H, ArH), 7.70 (d, *J* = 9.6 Hz, 1H, ArH), 7.52–7.47 (m, 3H, ArH), 7.41–7.37 (m, 1H, ArH), 7.20 (t, *J* = 8.0 Hz, 2H, ArH), 7.12 (d, *J* = 8.4 Hz, 1H, ArH), 6.94–6.89 (m, 1H, ArH), 6.81–6.76 (m, 1H, ArH), 6.61 (d, *J* = 8.0 Hz, 2H, ArH), 4.48 (s, 2H, CH_2_) ppm; ^13^C NMR (100 MHz, CDCl_3_), *δ* 163. 7, 161.8, 161.0, 158.7, 147.0, 136.2, 132.8, 131.4, 130.8, 129.5, 129.0, 128.1, 126.7, 119.5, 119.0, 118.7, 118.5, 113.2, 44.6 ppm.

**(4-((Phenylamino)methyl)-1*H*-pyrazol-3-yl)phenol** (**6**): White solid, 65% yield; m.p. 124–126 °C; ^1^H NMR (400 MHz, CDCl_3_), *δ* 7.62–7.58 (m, 2H, ArH), 7.24–7.19 (m, 3H, ArH), 7.05 (d, *J* = 8.0 Hz, 1H, ArH), 6.92–6.87 (m, 1H, ArH), 6.79–6.75 (m, 1H, ArH), 6.616 (d, *J* = 8.0 Hz, 2H, ArH), 4.40 (s, 2H, CH_2_) ppm; ^13^C NMR (100 MHz, CDCl_3_), *δ* 155.9, 147.7, 129.4, 129.4, 127.7, 119.7, 118.2, 117.2, 113.2 ppm; ESI-HRMS, m/z: Calcd for C_16_H_16_N_3_O^+^ [M + H]^+^: 266.1288, found: 266.1294.

## 4. Conclusions

In conclusion, a practical protocol for the one-pot synthesis of 3-aminoalkyl chromones was successfully developed through the domino aminomethylation and annulation of *o*-hydroxyphenyl enaminones by employing rongalite as a cost-effective and powerful in situ methylenating agent. The method exhibits a broad substrate scope and does not necessitate any additives or transition metal reagents, enabling efficient preparation of diverse 3-aminomethyl chromones. Gram-scale synthesis can also be successfully carried out, and the resulting products are amenable to various useful further transformations, which is expected to facilitate the further design and rapid synthesis of structurally diverse 3-aminoalkyl chromone derivatives.

## Figures and Tables

**Table 1:** Optimization of reaction conditions ^a^.

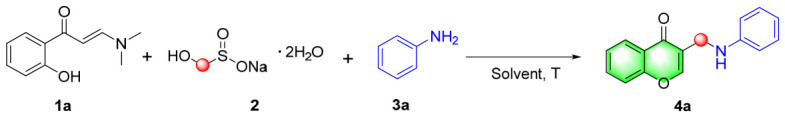			
Entry	Solvent	Temperature (°C)	Yield of 4a (%) ^b^
1	DMF	70	0
2	Toluene	70	16
3	MeCN	70	48
4	1,4-dioxane	70	36
5	EtOH	70	62
6	HOCH_2_CH_2_OH	70	66
7	HFIP	70	56
8	H_2_O	100	trace
9	CH_2_Cl_2_	50	58
10	DCE	70	83
11	DCE	80	80
12	DCE	60	72
13 ^c^	DCE	70	74
14 ^d^	DCE	70	83
15 ^e^	DCE	70	88

^a^ Reaction conditions: **1a** (0.2 mmol, 1.0 equiv.), **2** (0.3 mmol, 1.5 equiv.), **3a** (0.3 mmol, 1.5 equiv.), solvent (1.0 mL), 70 °C, 12 h. ^b^ Isolated yields based on **1a**; ^c^ 6 h; ^d^ 18 h; ^e^ **3a** (0.4 mmol, 2 equiv.).

## Data Availability

The original contributions presented in this study are included in the article/Supplementary Material. Further inquiries can be directed to the corresponding authors.

## Supplementary Materials

The following supporting information can be downloaded at https://www.mdpi.com/article/10.3390/molecules31091432/s1, ^1^H, ^13^C and ^19^F NMR spectra for all compounds **4a**–**4z**, **5** and **6**.

## Abbreviations

The following abbreviations are used in this manuscript:

DMF	*N*,*N*-dimethylformamide
DCE	1,2-dichloroethane
HFIP	1,2-dichloroethane
