# Supplementary material for: Catalyst-Free Three-Component Synthesis of 3-Aminoalkyl Chromones Using Rongalite as a C1 Synthon
Source: Molecules. 2026 Apr 26;31(9):1432. doi: 10.3390/molecules31091432 (PMC13165258; doi:10.3390/molecules31091432)

Supplementary Materials

Catalyst-free three-component synthesis of 3-aminoalkyl chromones using rongalite as a C1 synthon

Juanjuan Gao [1], Xinlei Fu [1], Ruoying Hu [1], Jinhua Wan [1], Kai Yang [1],

Zhaowen Liu [1,*], Zhiqin Huang [1,*]

[1] Jiangxi Province Key Laboratory of Pharmacology of Traditional Chinese Medicine, School of pharmacy, Gannan Medical University, Ganzhou 341000, P. R. China; gaoya0758@163.com (J.G.); fxl05020502@163.com (X.L.); 18270932058@163.com (R.H.); 13007261183@163.com (J.W.); kai_yangyang@126.com (K.Y.).

* Correspondence: liuzhaowen@gmu.edu.cn (Z.L.); hzqzll@126.com (Z.H.).

Table of Contents

Experimental Procedure for Compounds 1a-1l..[S2]

Control experiments..[S3]

References...[S4]

NMR Spectra..[S5]

Experimental Procedure for Compounds 1a-1l

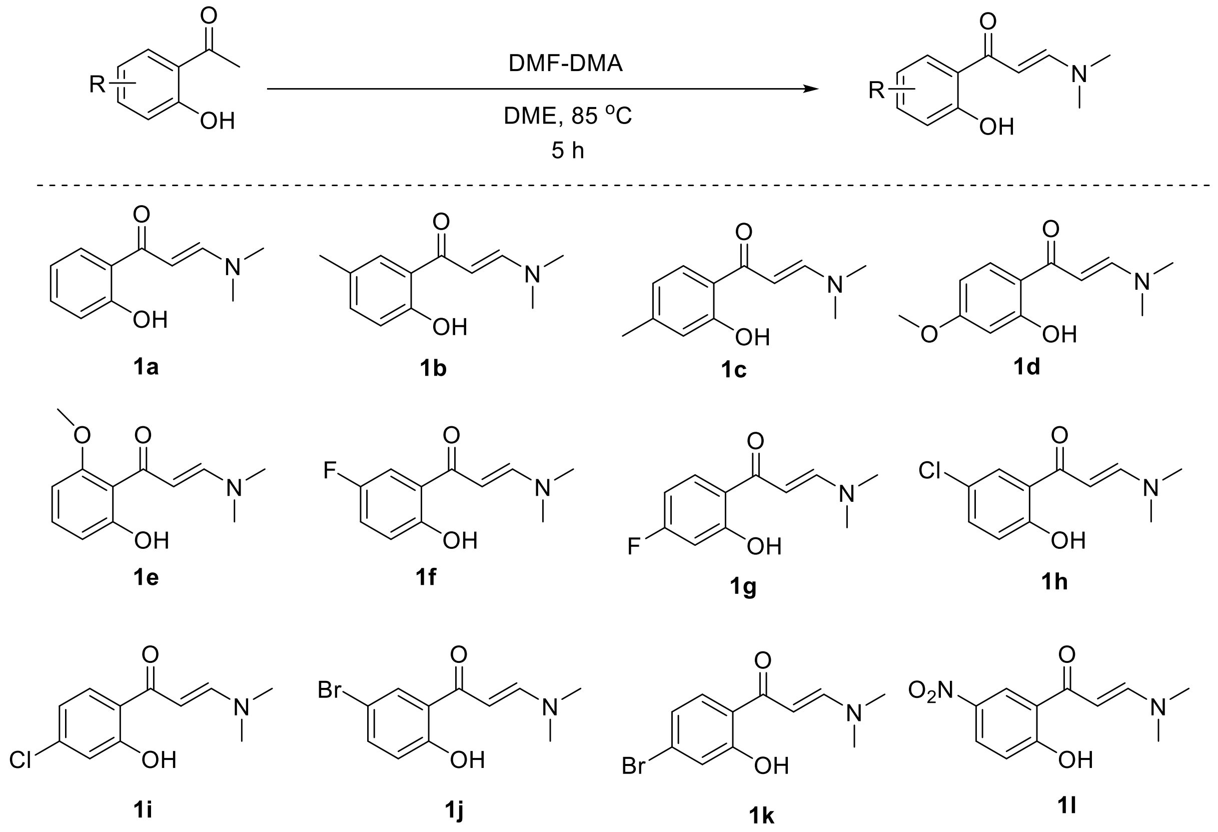

Scheme S1

According to the reported procedure [1,2], compounds 1a-1l were synthesized. 2'-Hydroxyacetophenone (20.0 mmol, 1.0 equiv) and N,N-Dimethylformamide dimethyl acetal (60.0 mmol, 3.0 equiv) in DMA (30.0 mL) was refluxed for 5 h. After monitoring the end of the reaction on TLC, the mixture was cooled to room temperature. Upon completion of the reaction, the resulting mixture was concentrated in vacuo. The crude product is recrystallized in petroleum ether to obtain the required compound **1a**-**1l**.

Control experiments

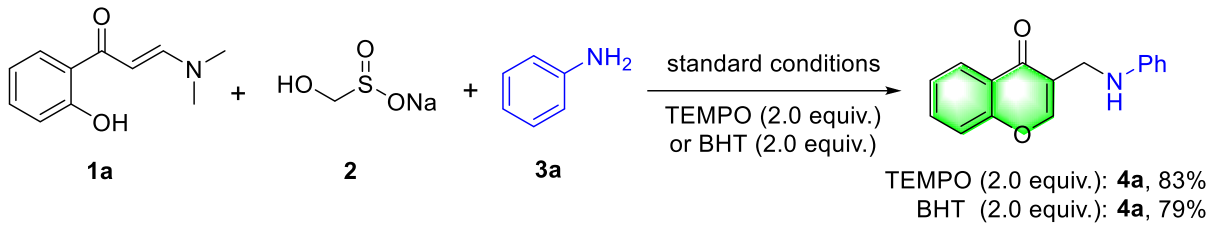

Scheme S2

In a 25 mL oven-dried roundbottom flask, enaminone **1a** (0.2 mmol, 1 equiv.), rongalite **2** (0.3 mmol, 1.5 equiv.), amine **3a** (0.4 mmol, 2.0 equiv.) and 2,2,6,6-Tetramethyl-1-piperinedinyloxy (TEMPO) (0.4 mmol, 2 equiv.) or 2,2,6,6-tetramethyl-1-piperidinyloxy (BHT) (0.4 mmol, 2 equiv.) were dissolved in DCE and stirred for 12 h at 70 °C. After the reaction was complete, the solvent was evaporated, and the residue was dissolved in ethyl acetate and washed with brine. The organic layer was dried over anhydrous sodium sulfate, concentrated, and the crude mixture was purified using silica gel column chromatography with ethyl acetate and petroleum ether as the eluent to isolate the product **4**.

References

1. Wu, L.-H.;Liu, X.; Liu, Z.-W.; Chen, Z.-X.; Fu, X.-L.; Yang, K. Metal-free synthesis of difluoro/trifluoromethyl carbinol-containing chromones via tandem cyclization of o-hydroxyaryl enaminone. *Org. Biomol. Chem*. **2023**, *21*, 9236-9241.
2. Yang, K.; Wu, L.; Fu, X.; Chen, W.; Liu, Z.; Peng, X. HFIP promoted cyclization of o-hydroxyaryl enaminones with chlorohydrocarbon for synthesis of chromone-containing triarylmethanes. *Tetrahedron*, **2024**, *156*, 133948.

NMR Spectra

^{1}H NMR spectrum of compound 4a

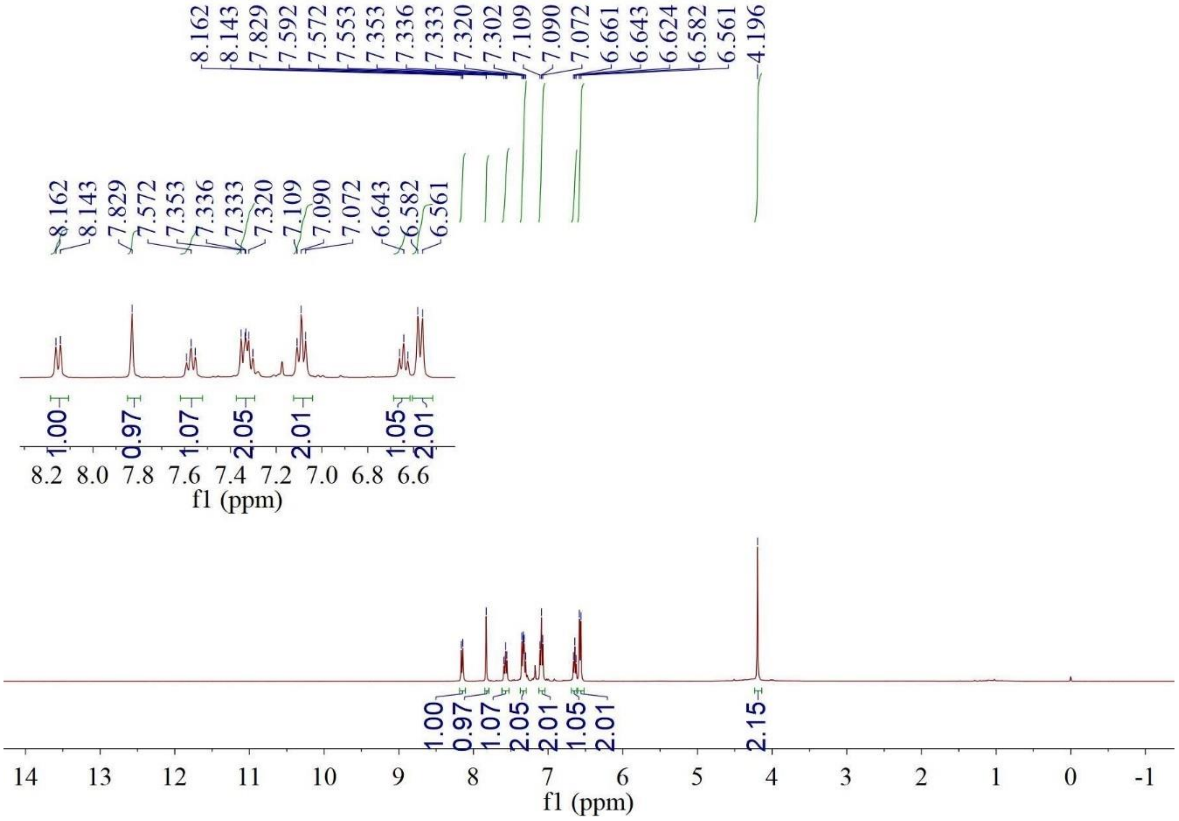

^{13}C NMR spectrum of compound 4a

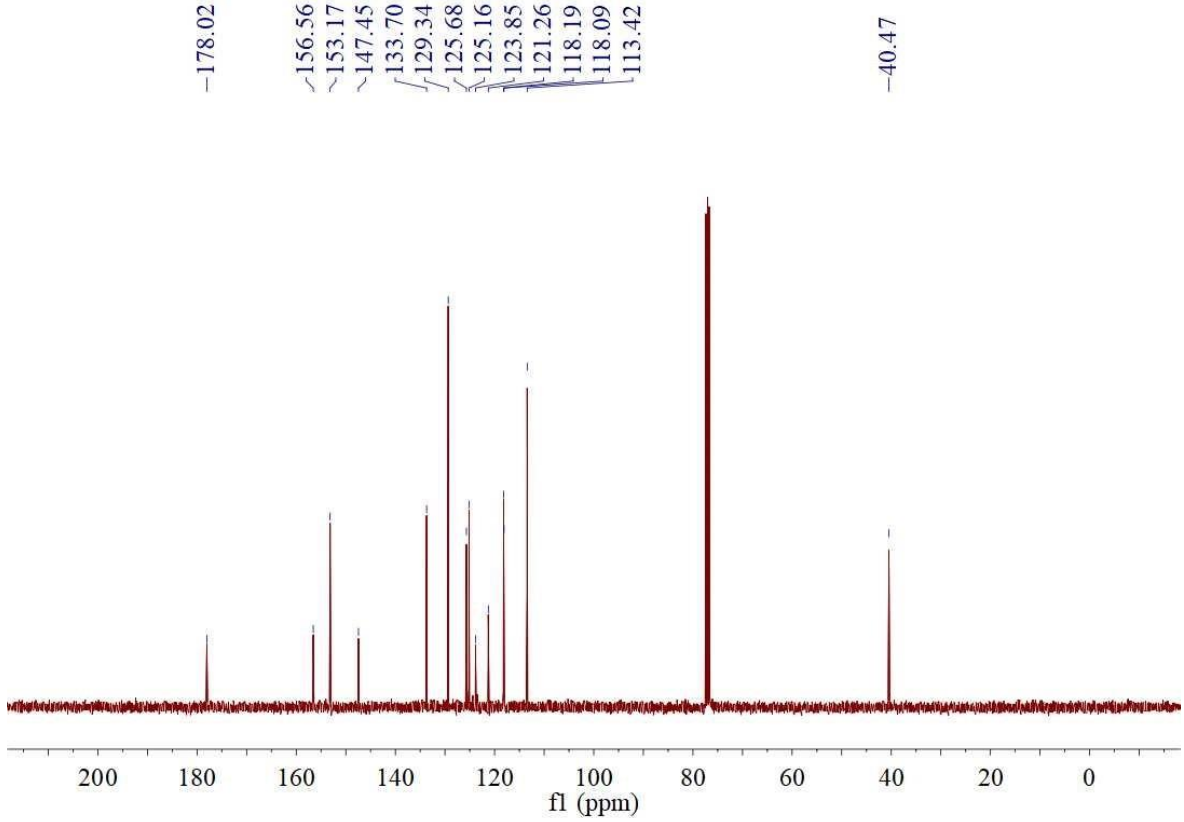

^{1}H NMR spectrum of compound 4b

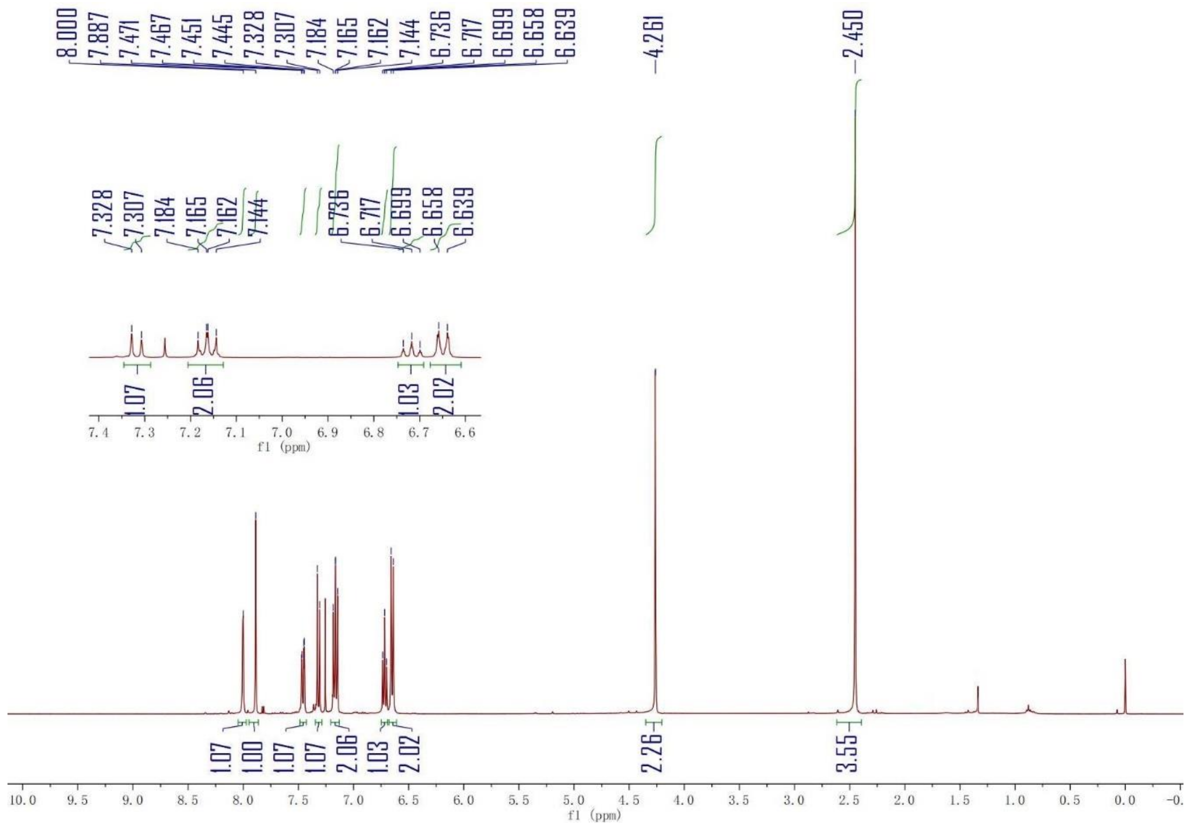

^{13}C NMR spectrum of compound 4b

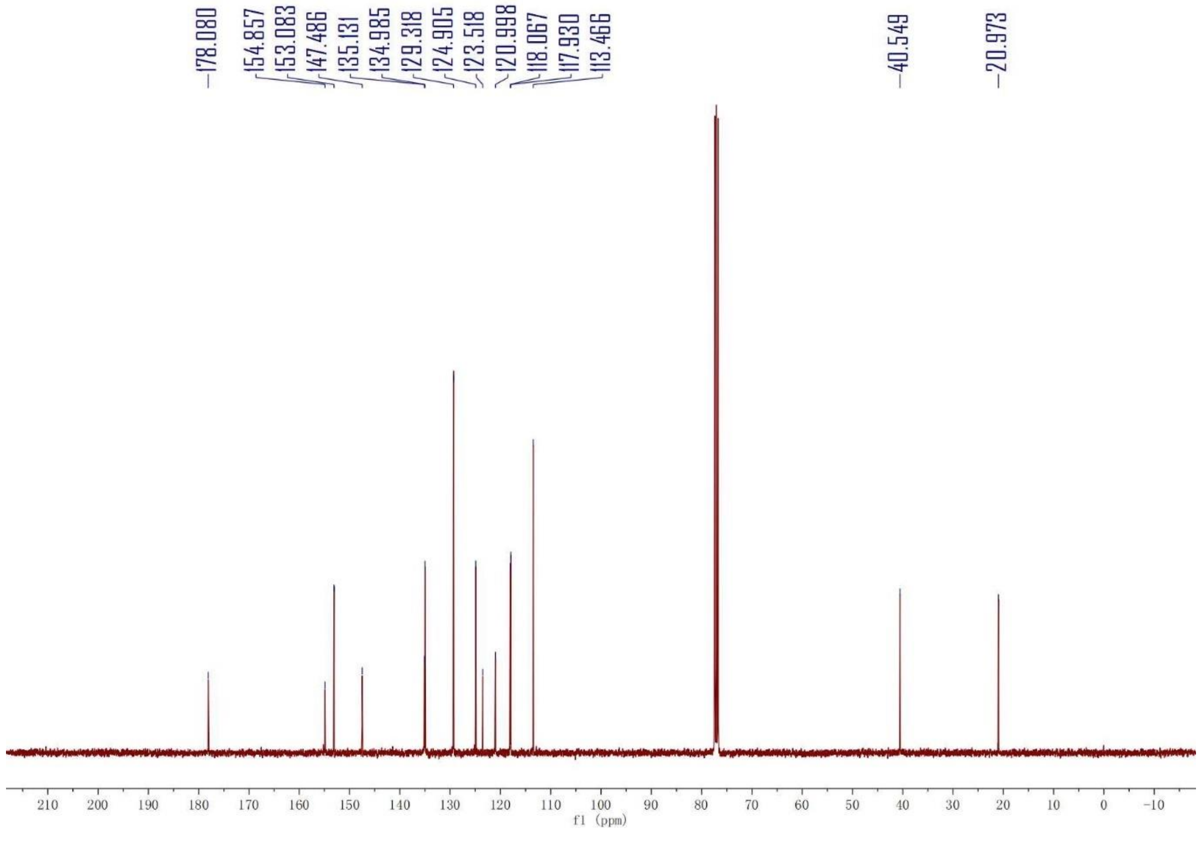

^{1}H NMR spectrum of compound 4c

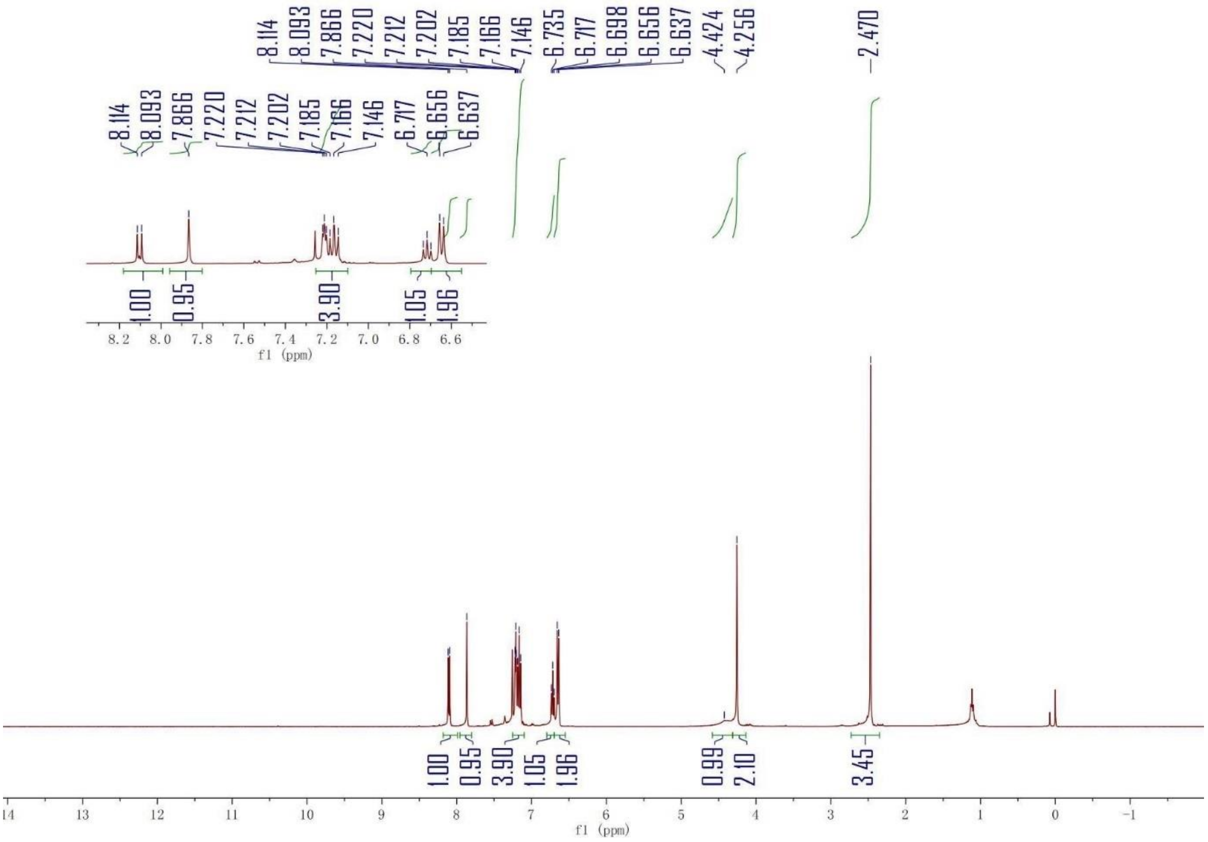

^{13}C NMR spectrum of compound 4c

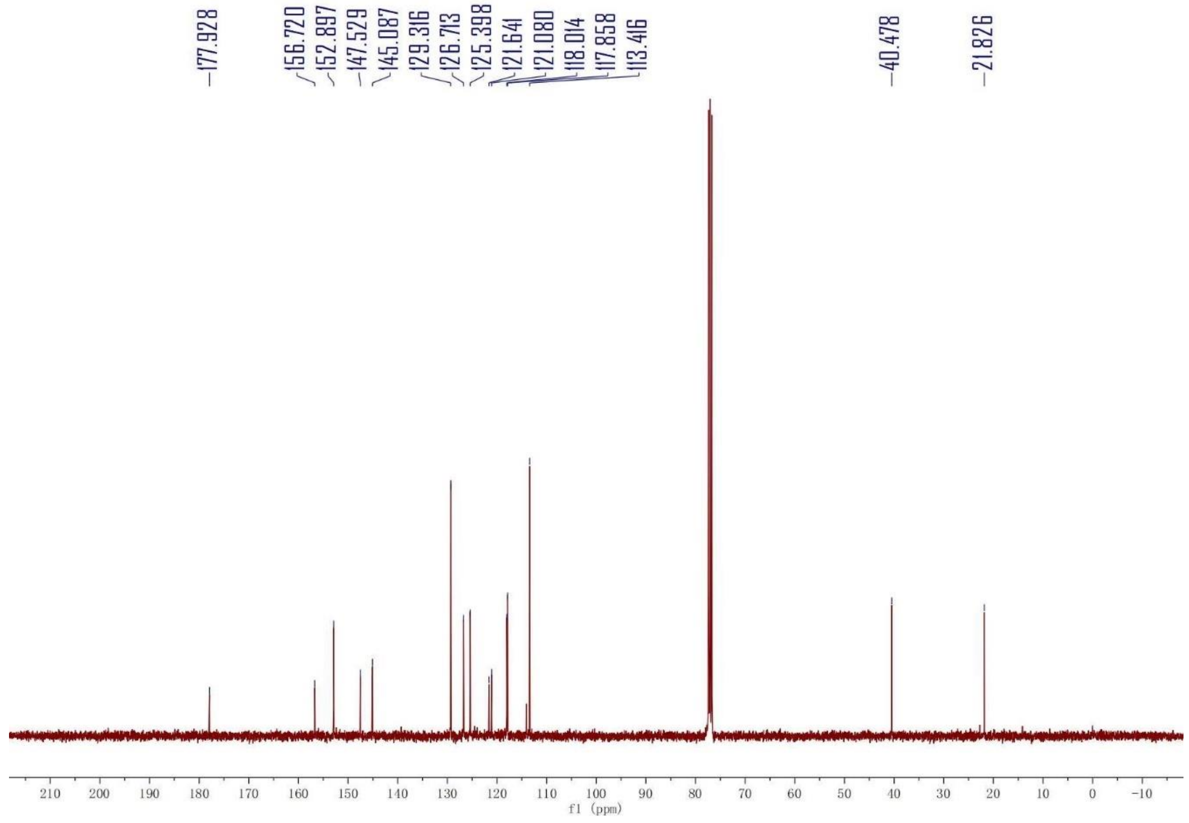

^{1}H NMR spectrum of compound 4d

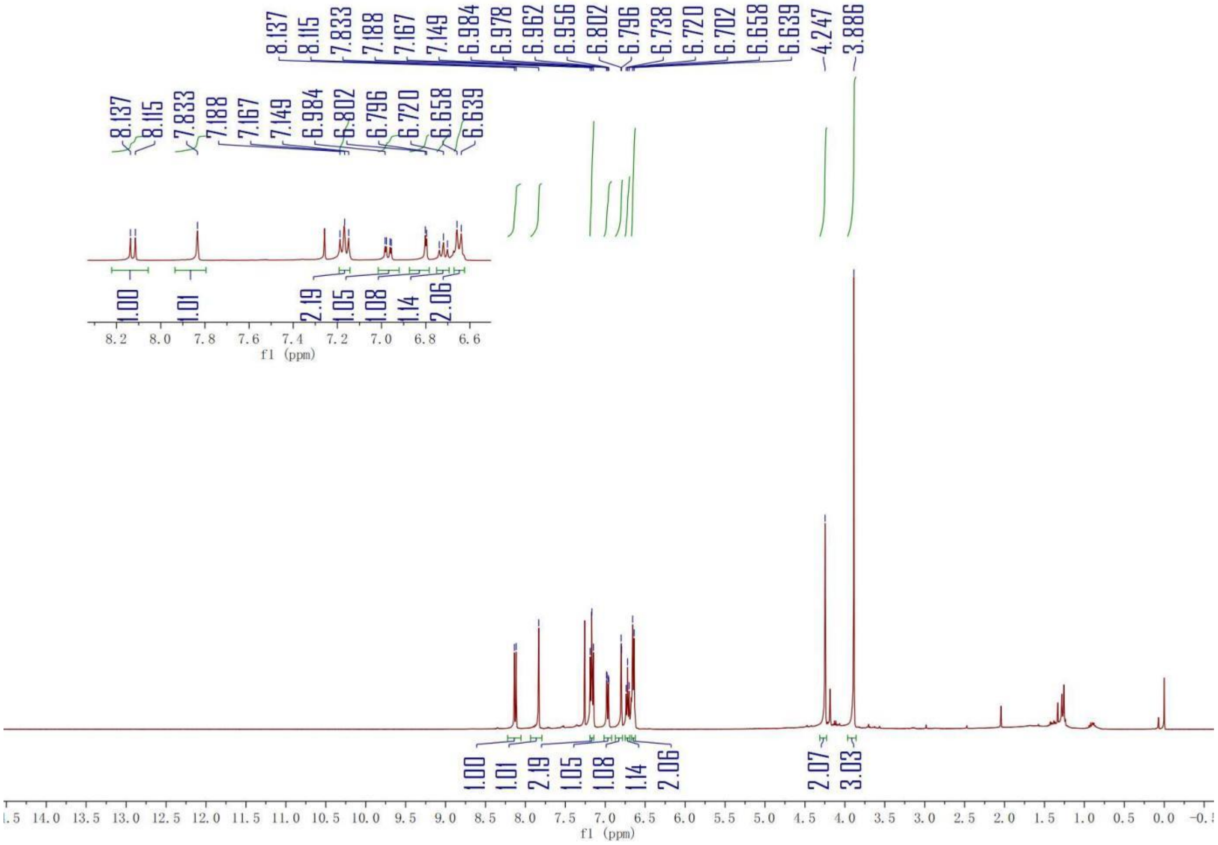

^{13}C NMR spectrum of compound 4d

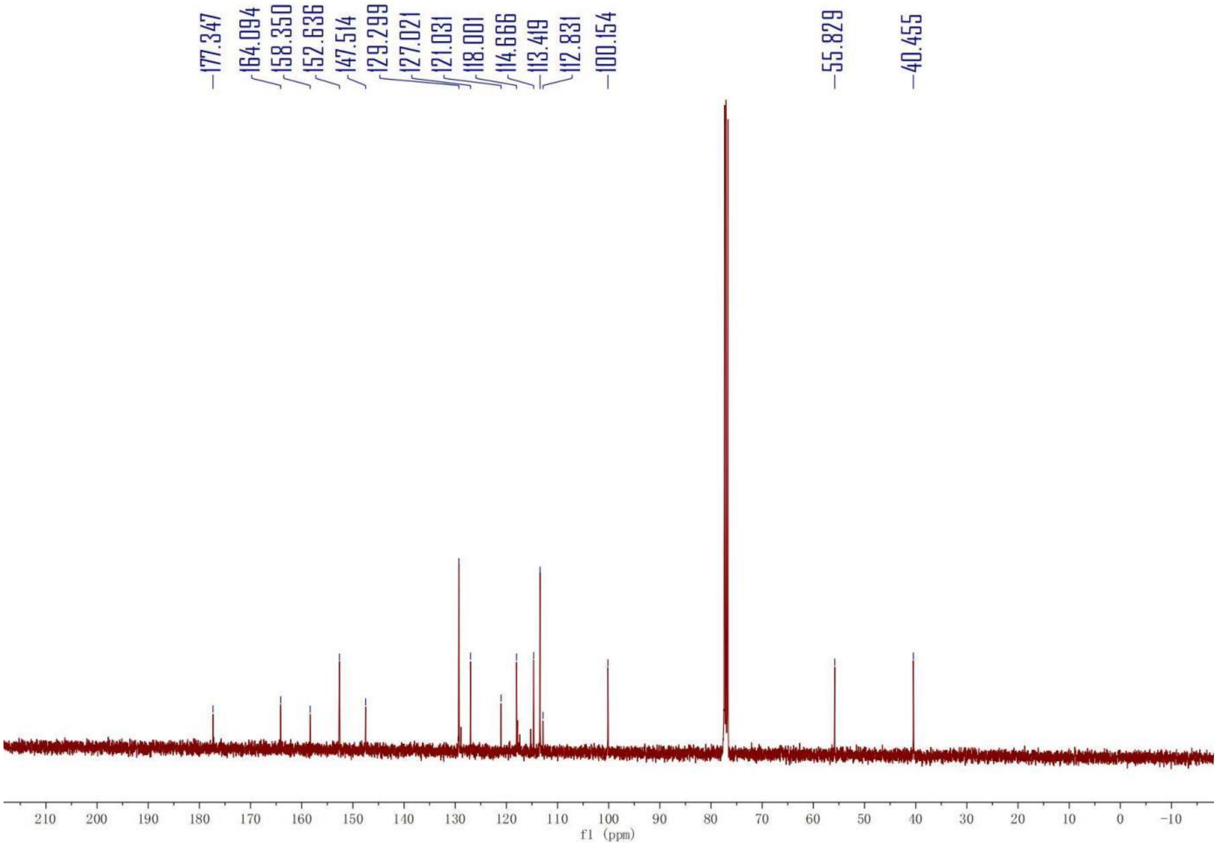

^{1}H NMR spectrum of compound 4e

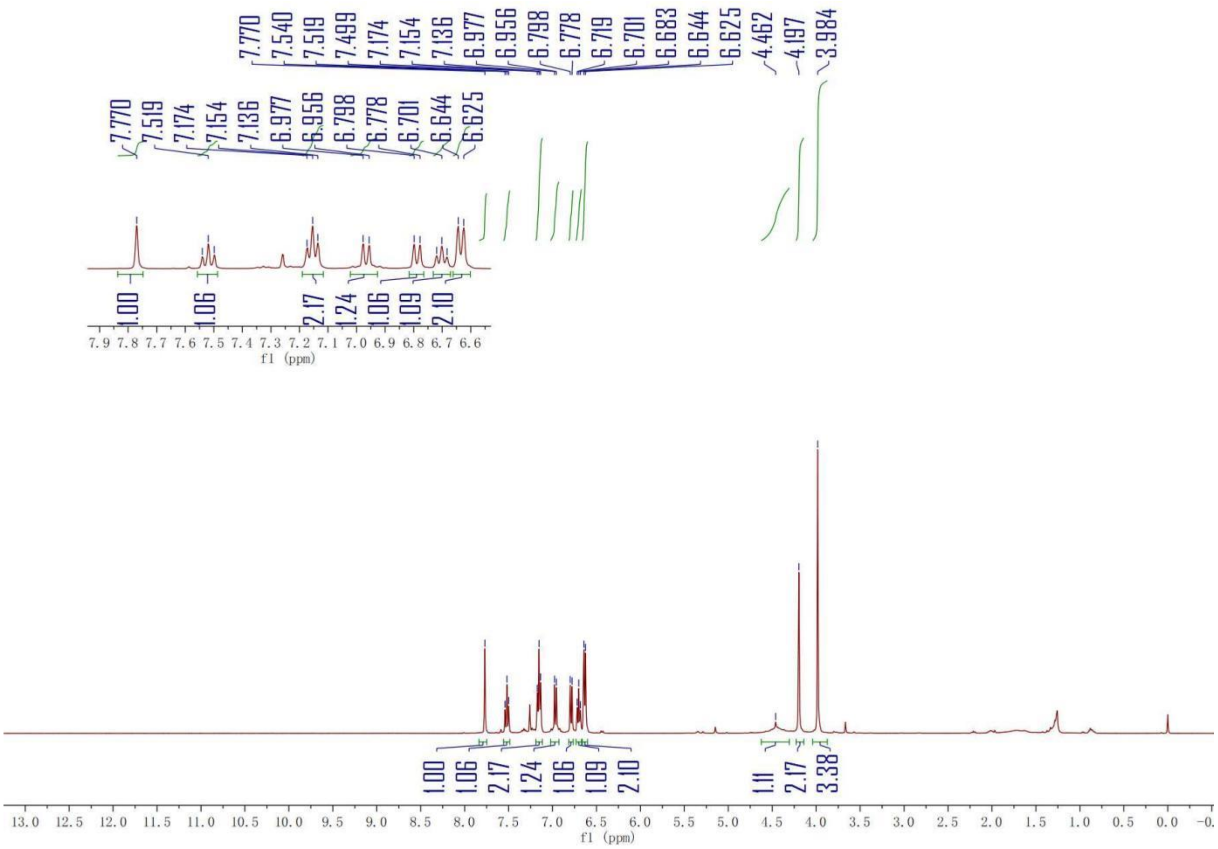

^{13}C NMR spectrum of compound 4e

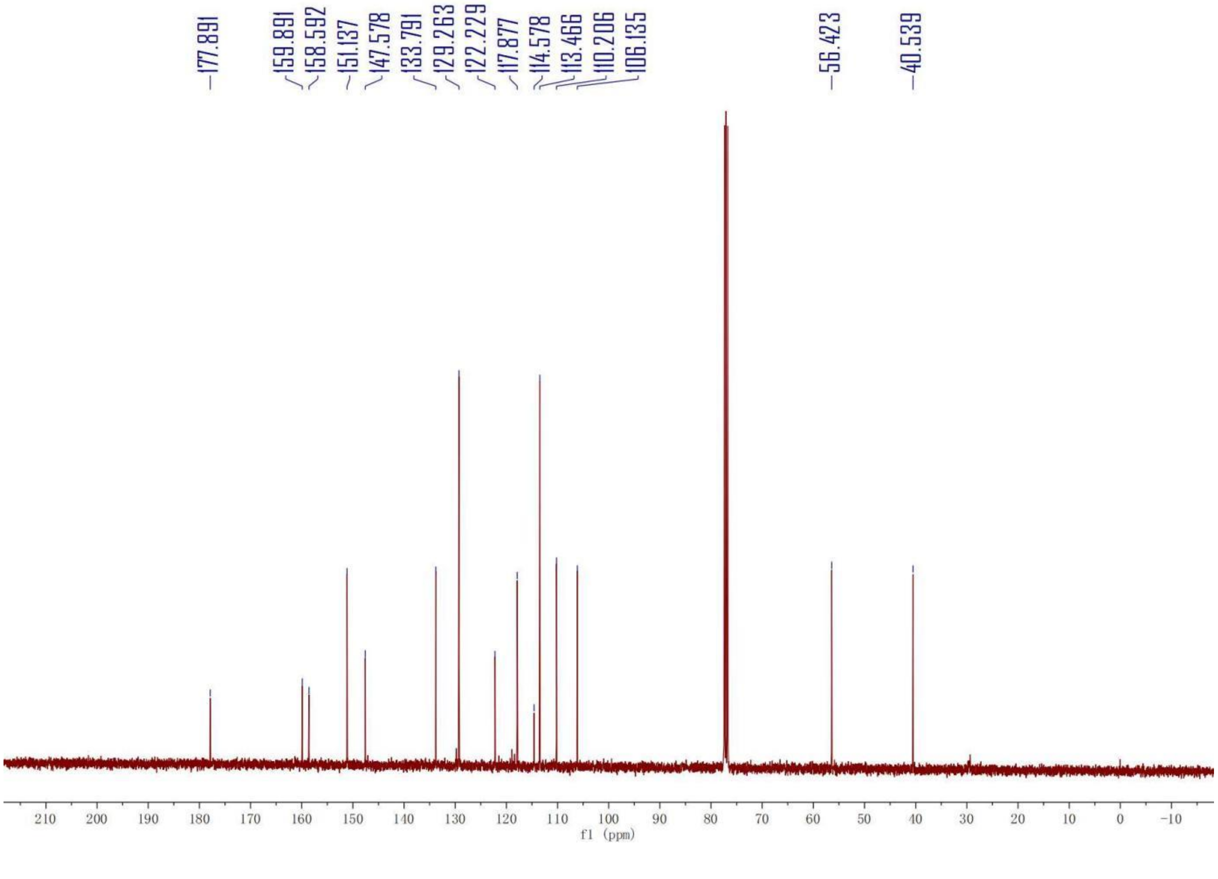

^{1}H NMR spectrum of compound 4f

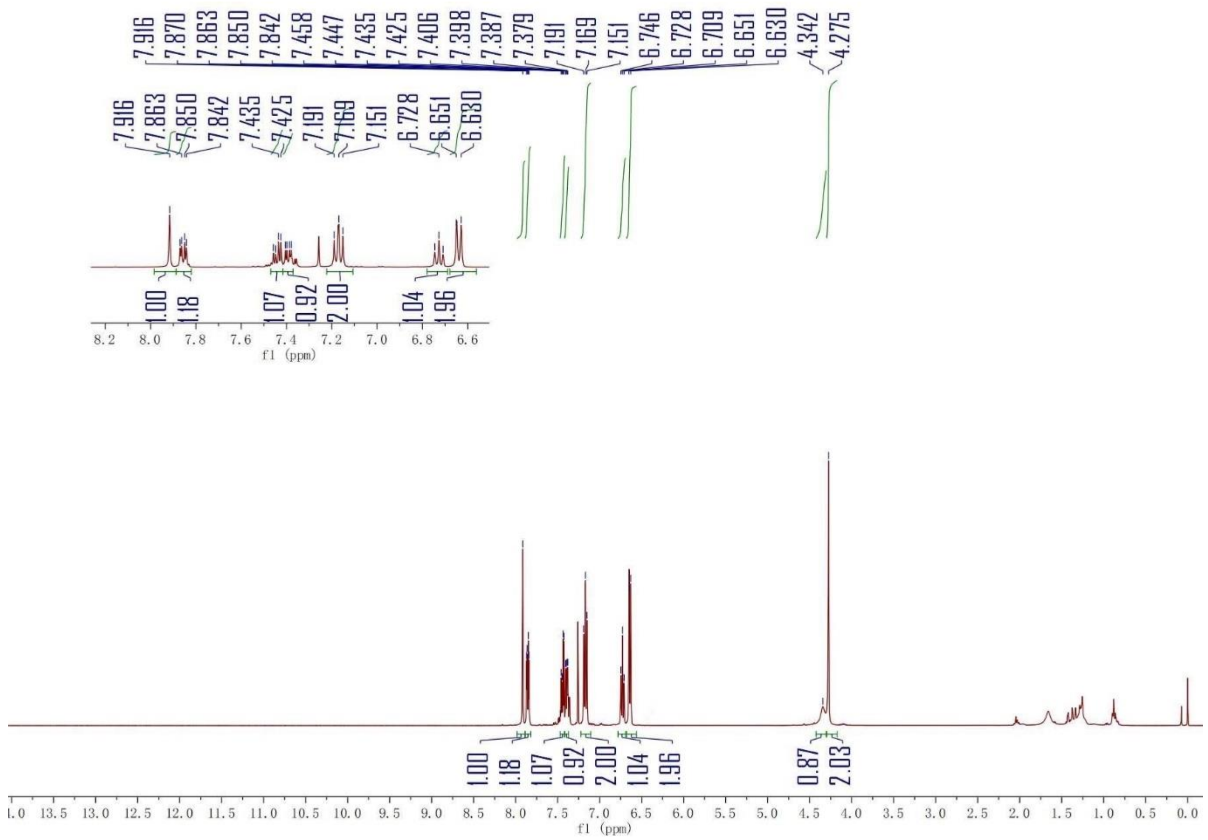

^{13}C NMR spectrum of compound 4f

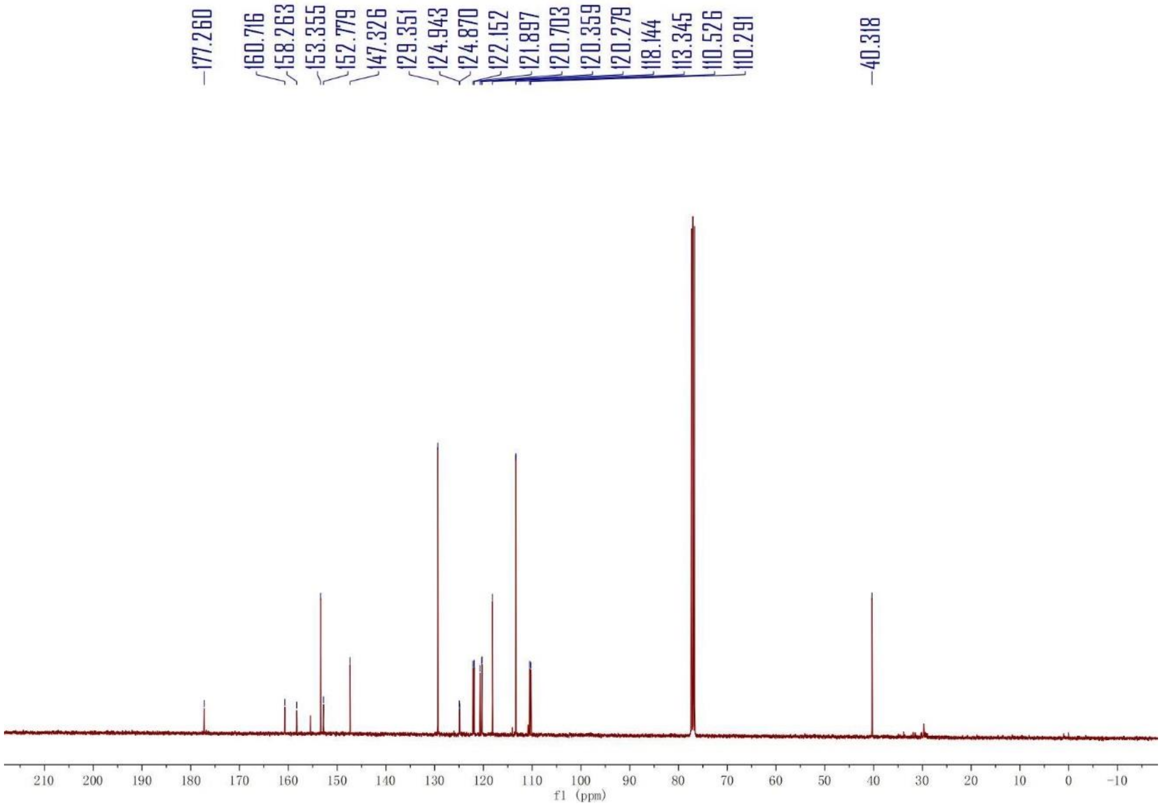

^{19}F NMR spectrum of compound 4f

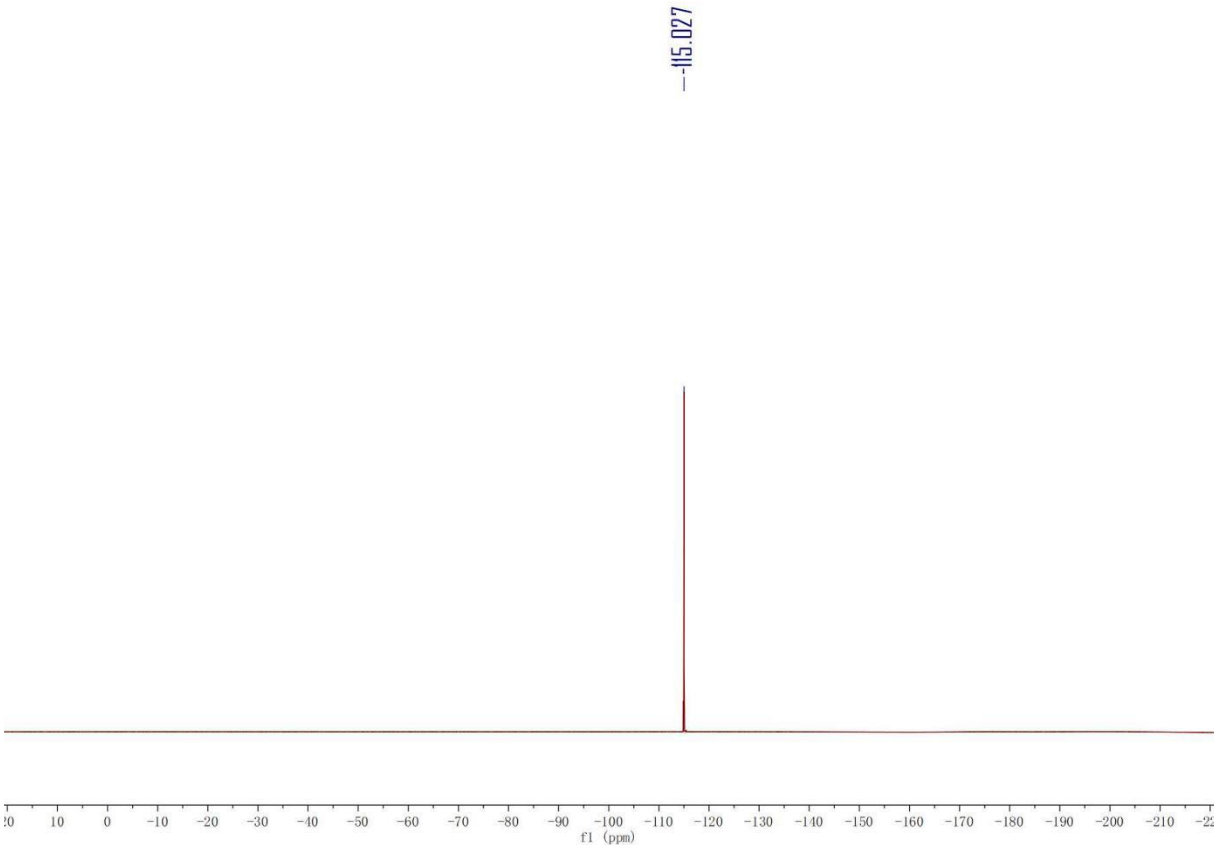

^{1}H NMR spectrum of compound 4g

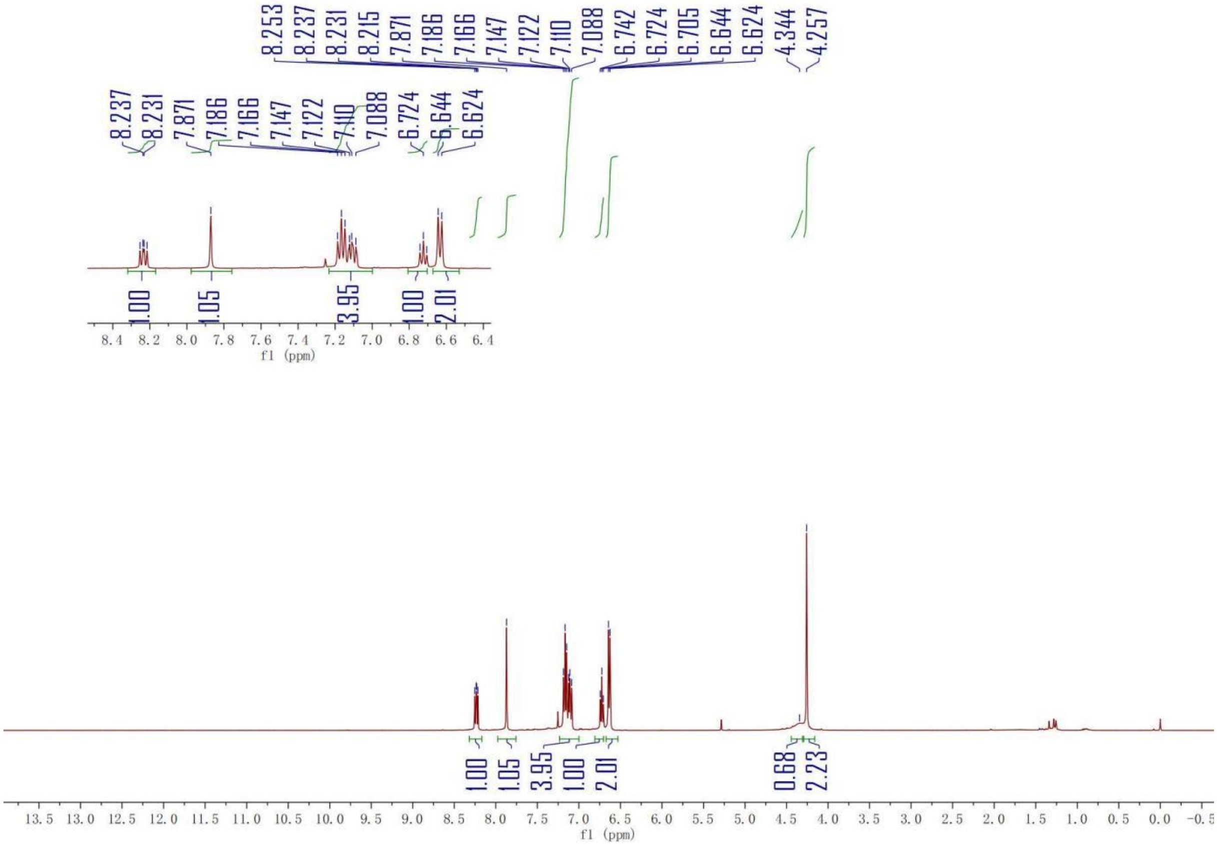

^{13}C NMR spectrum of compound 4g

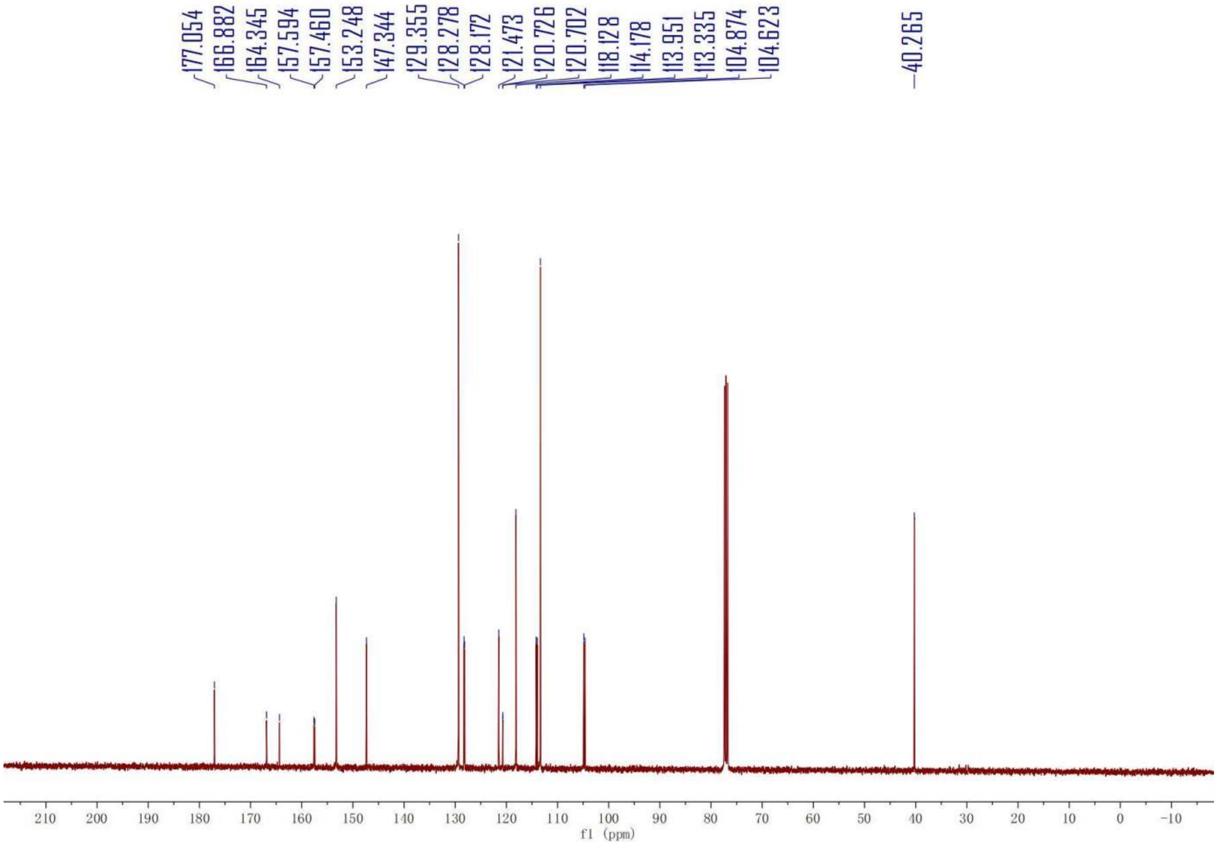

^{19}F NMR spectrum of compound 4g

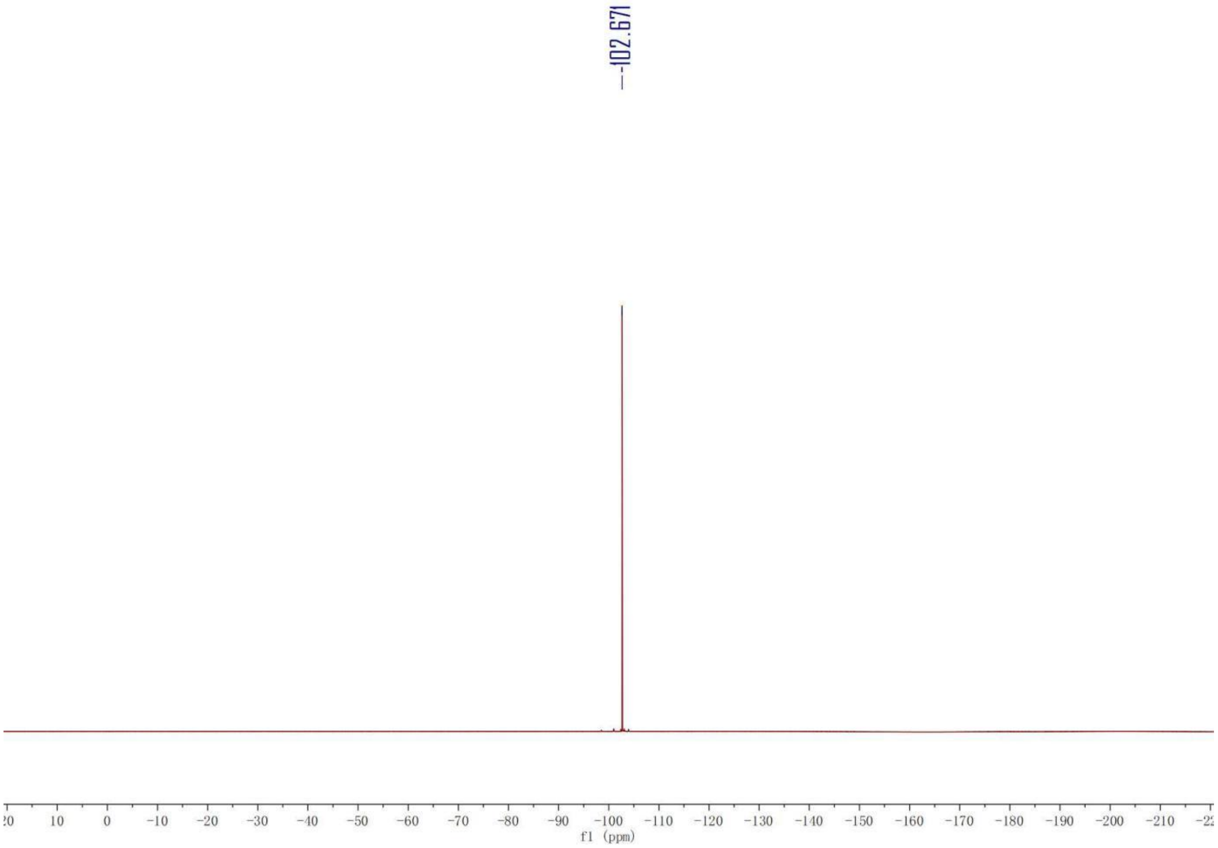

^{1}H NMR spectrum of compound 4h

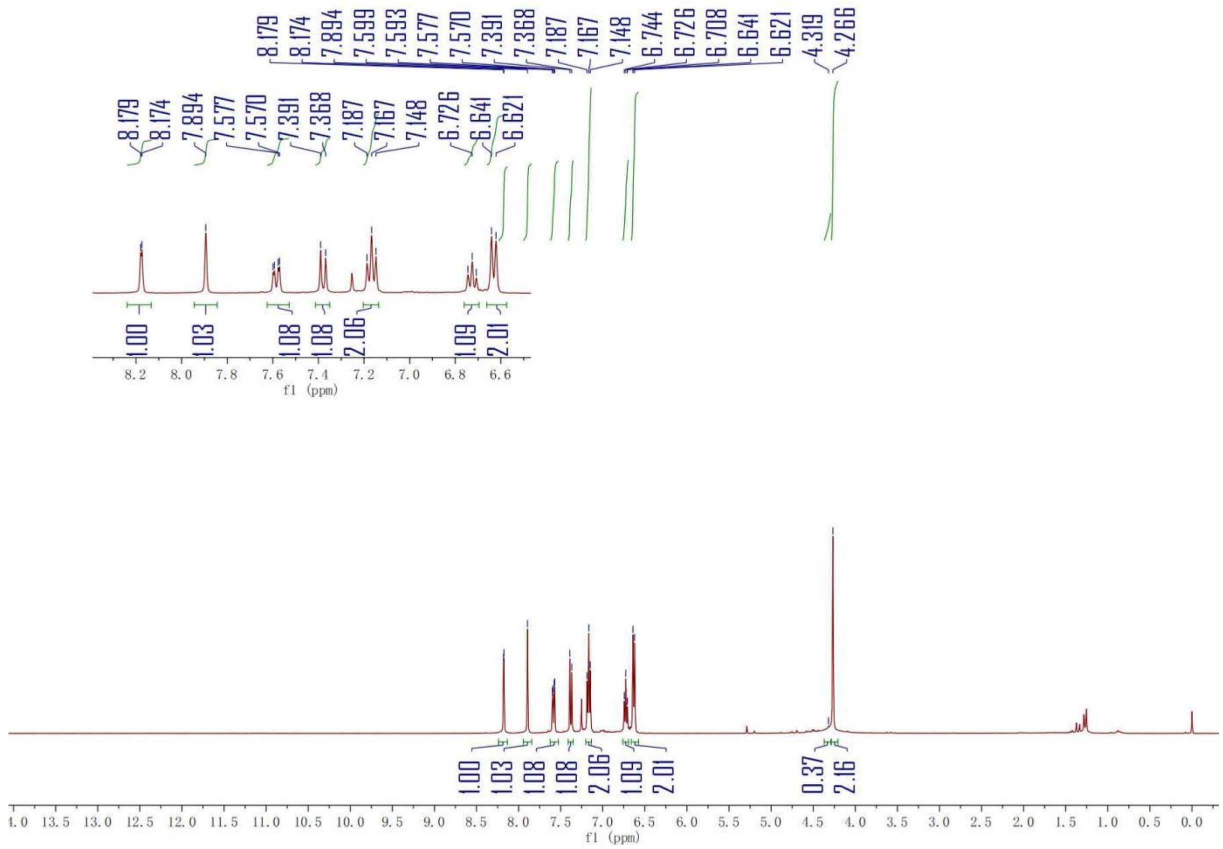

^{13}C NMR spectrum of compound 4h

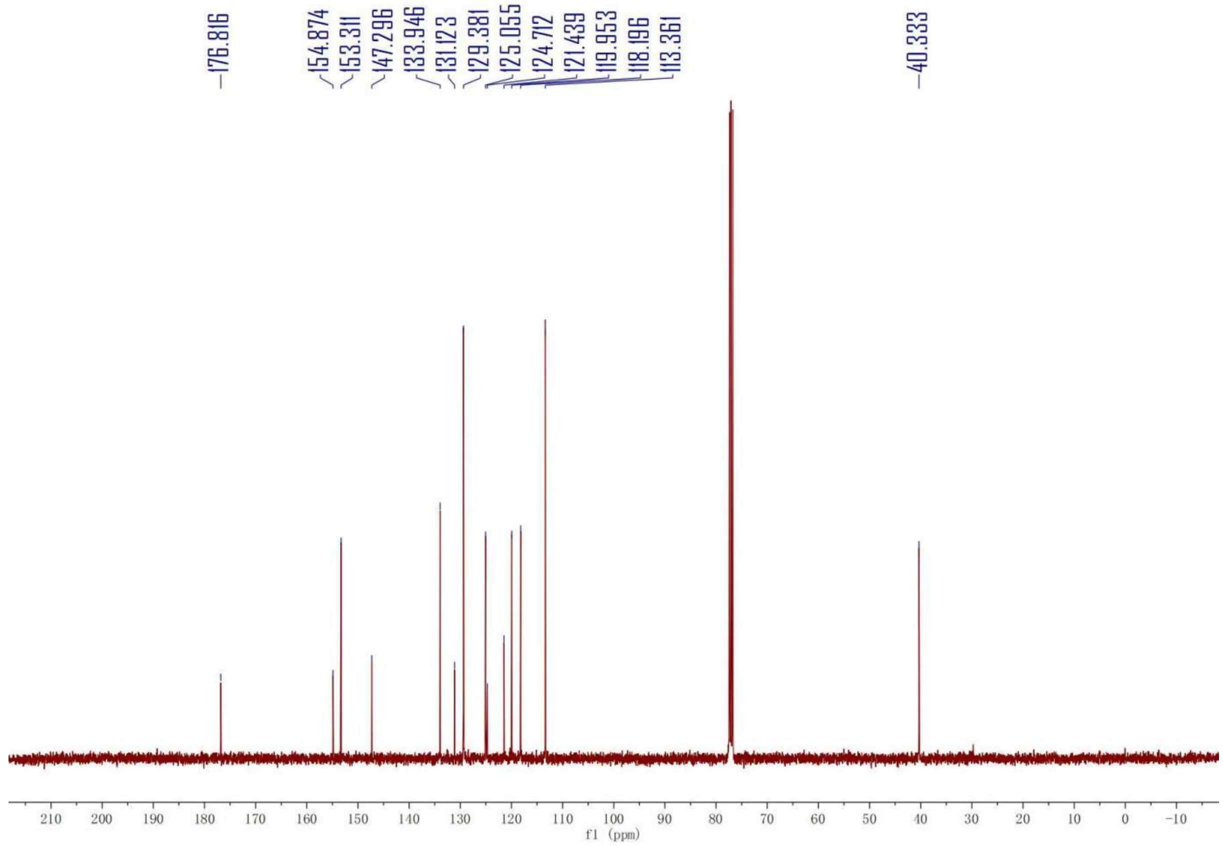

^{1}H NMR spectrum of compound 4i

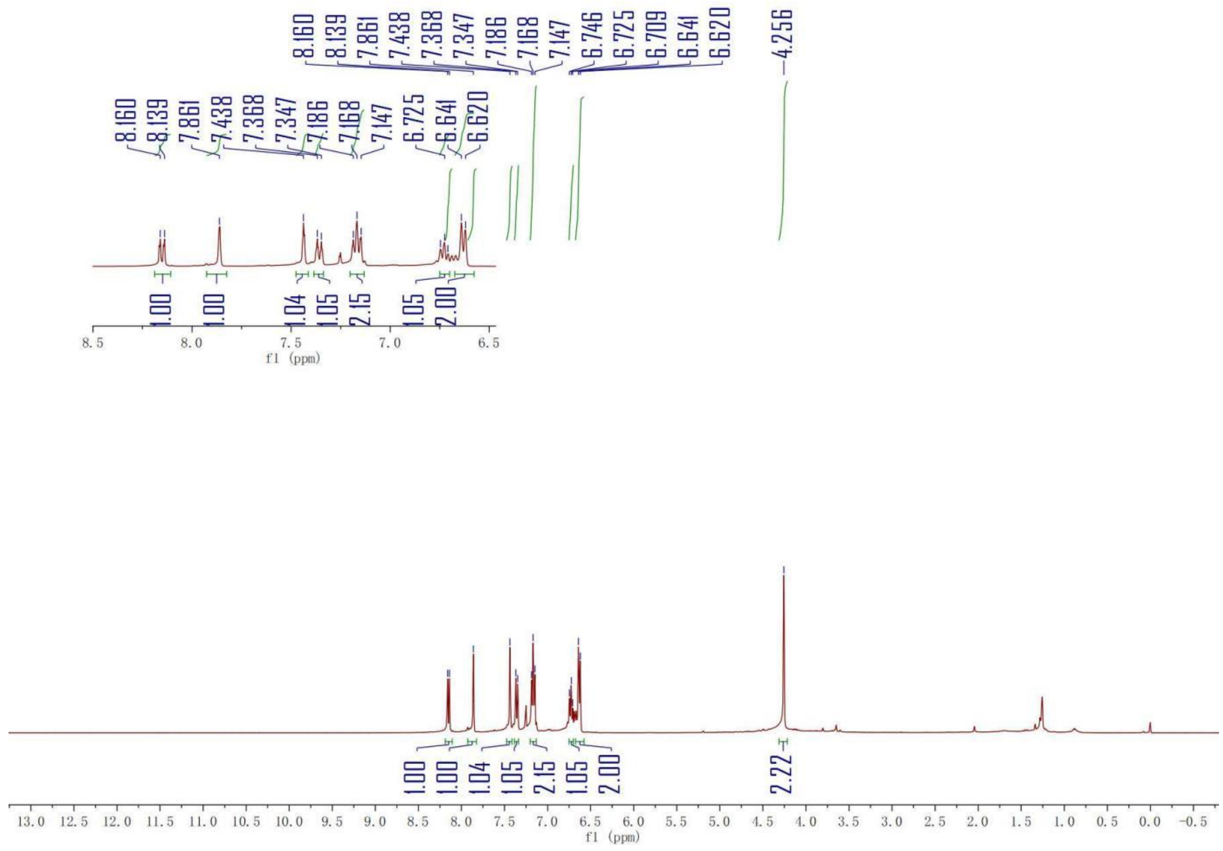

^{13}C NMR spectrum of compound 4i

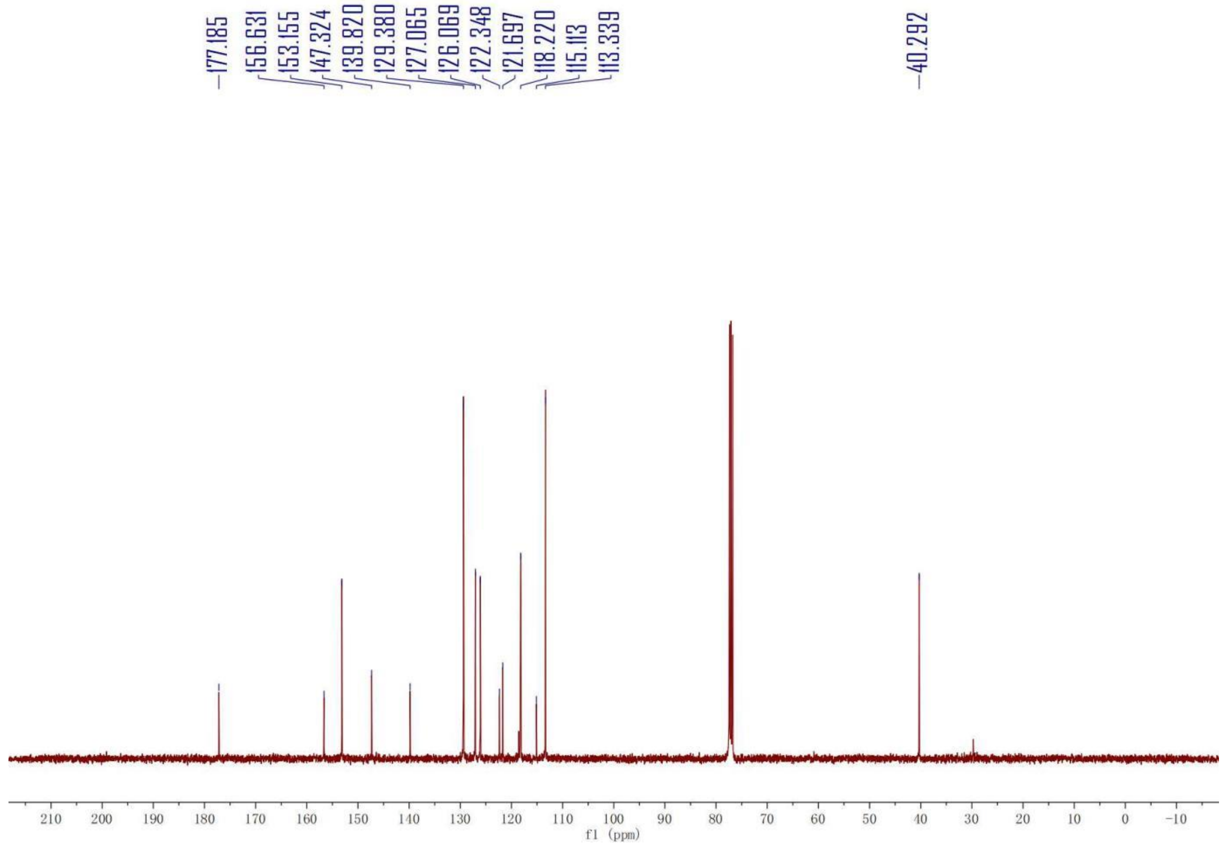

^{1}H NMR spectrum of compound 4j

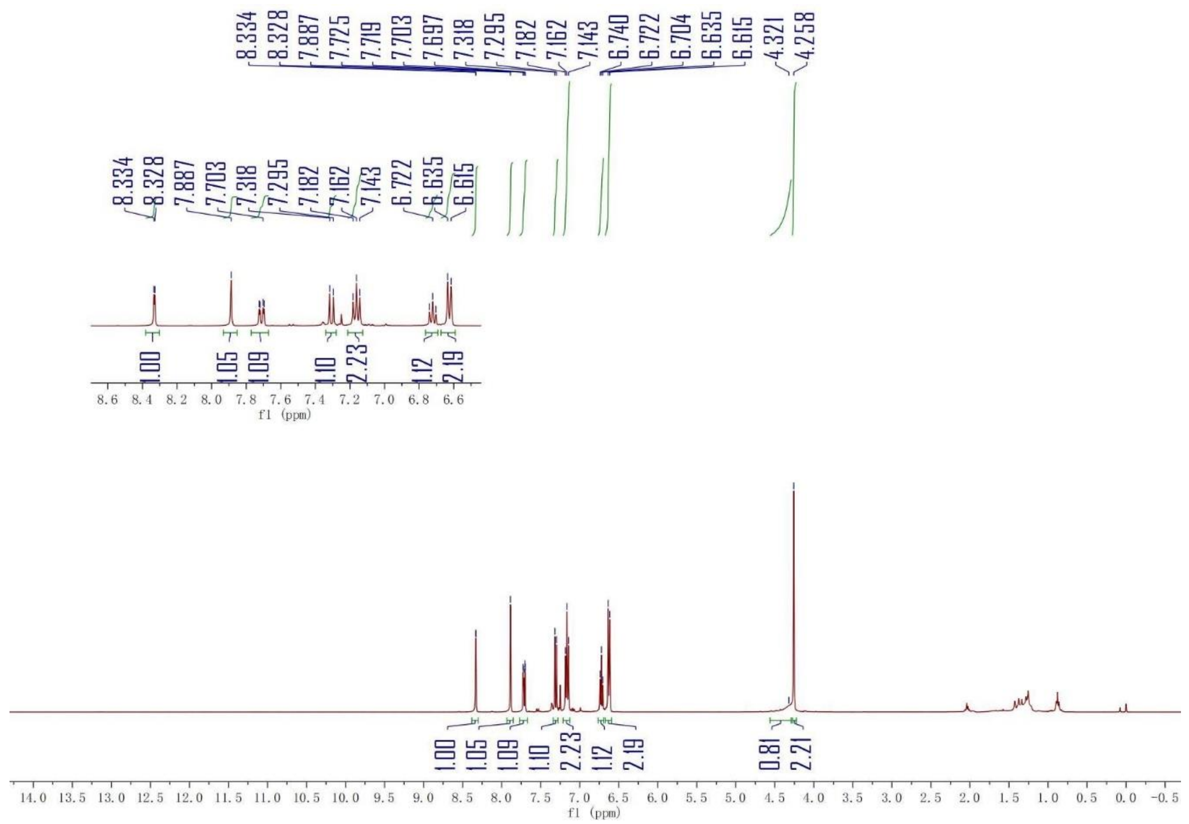

^{13}C NMR spectrum of compound 4j

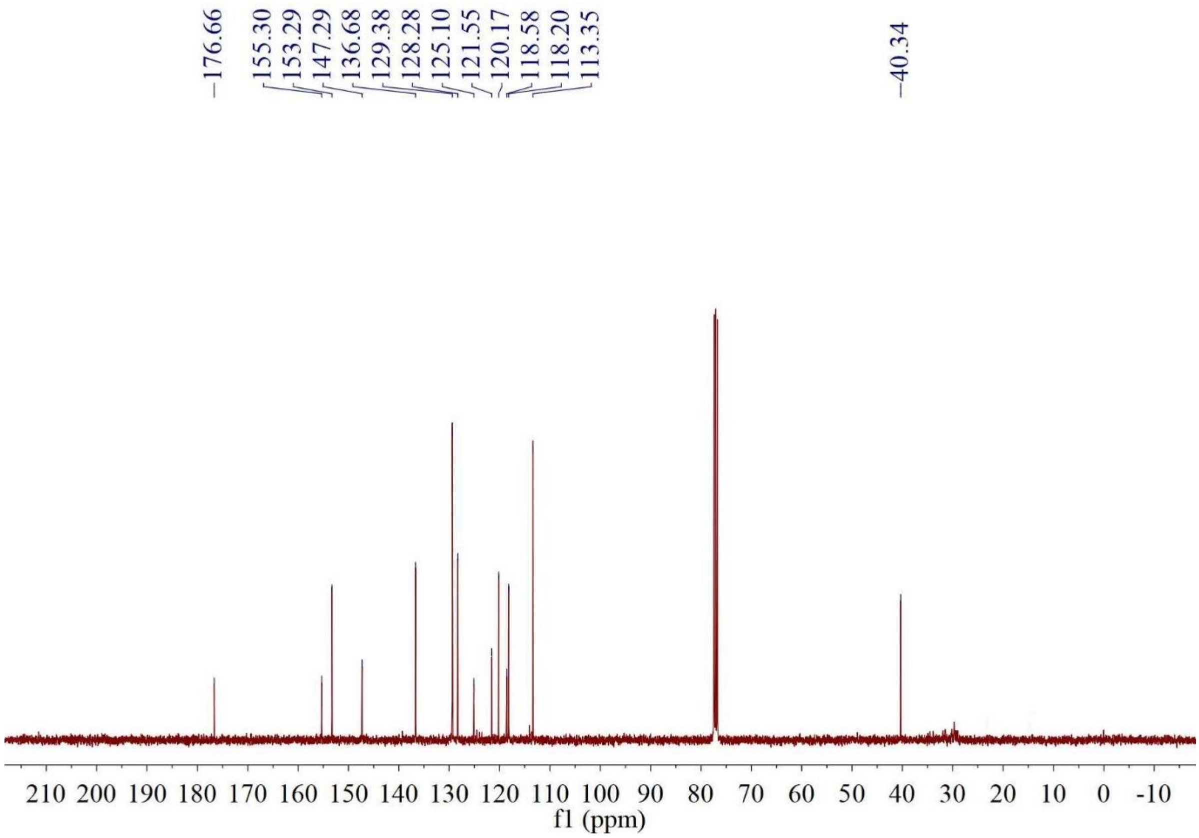

^{1}H NMR spectrum of compound 4k

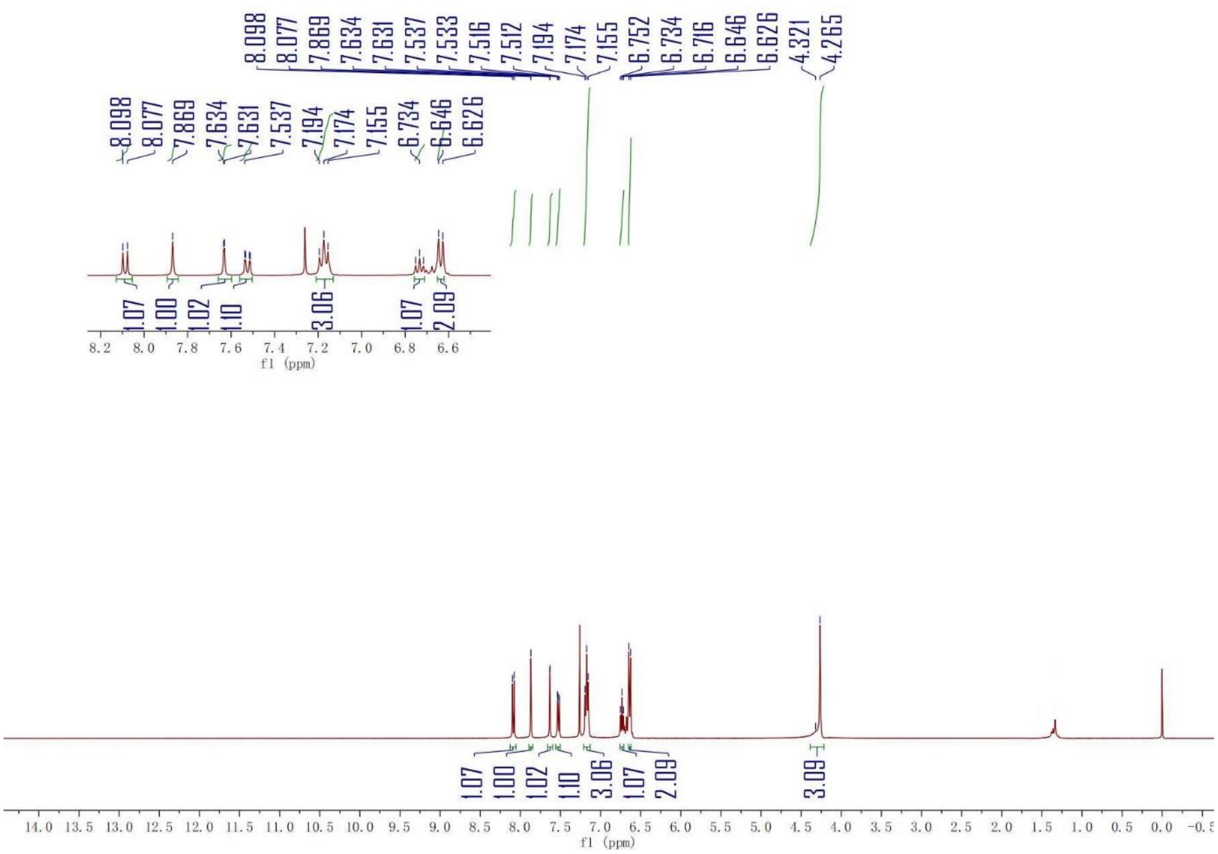

^{13}C NMR spectrum of compound 4k

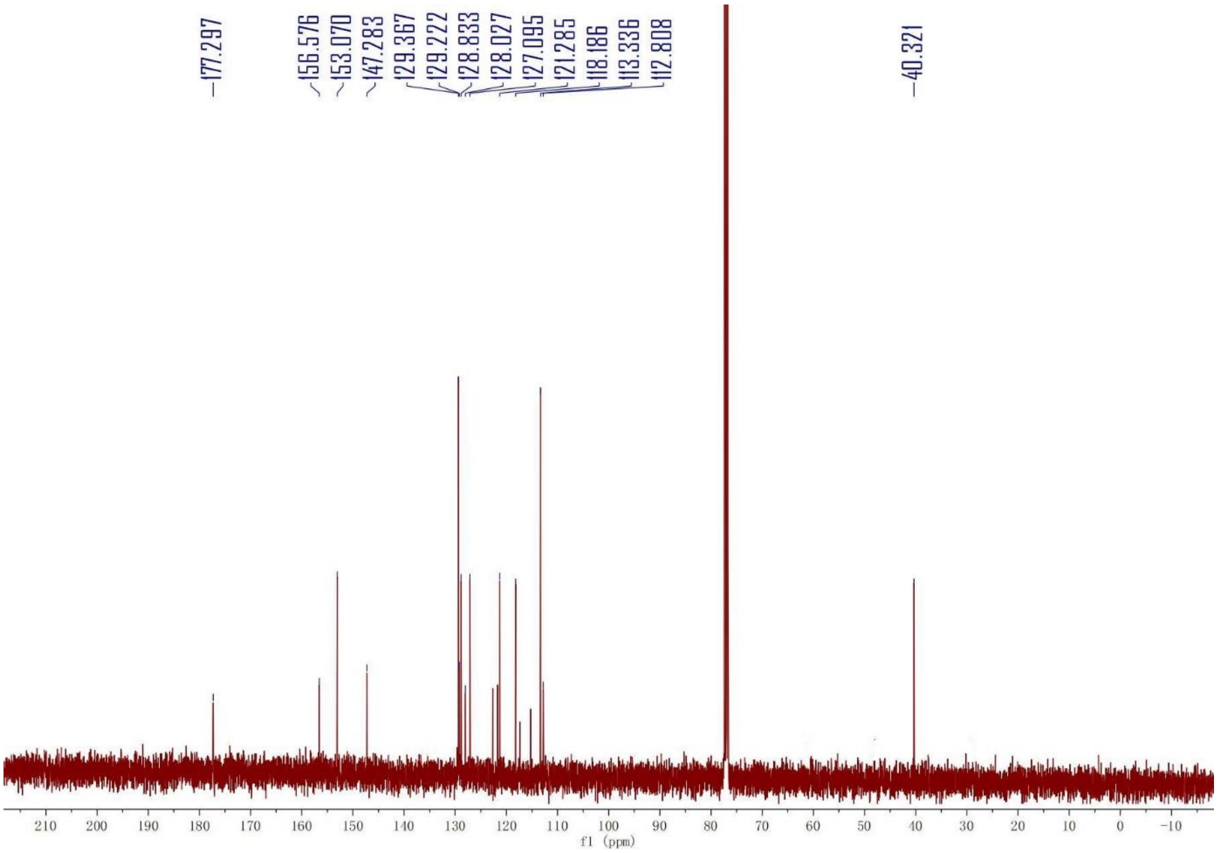

^{1}H NMR spectrum of compound 4l

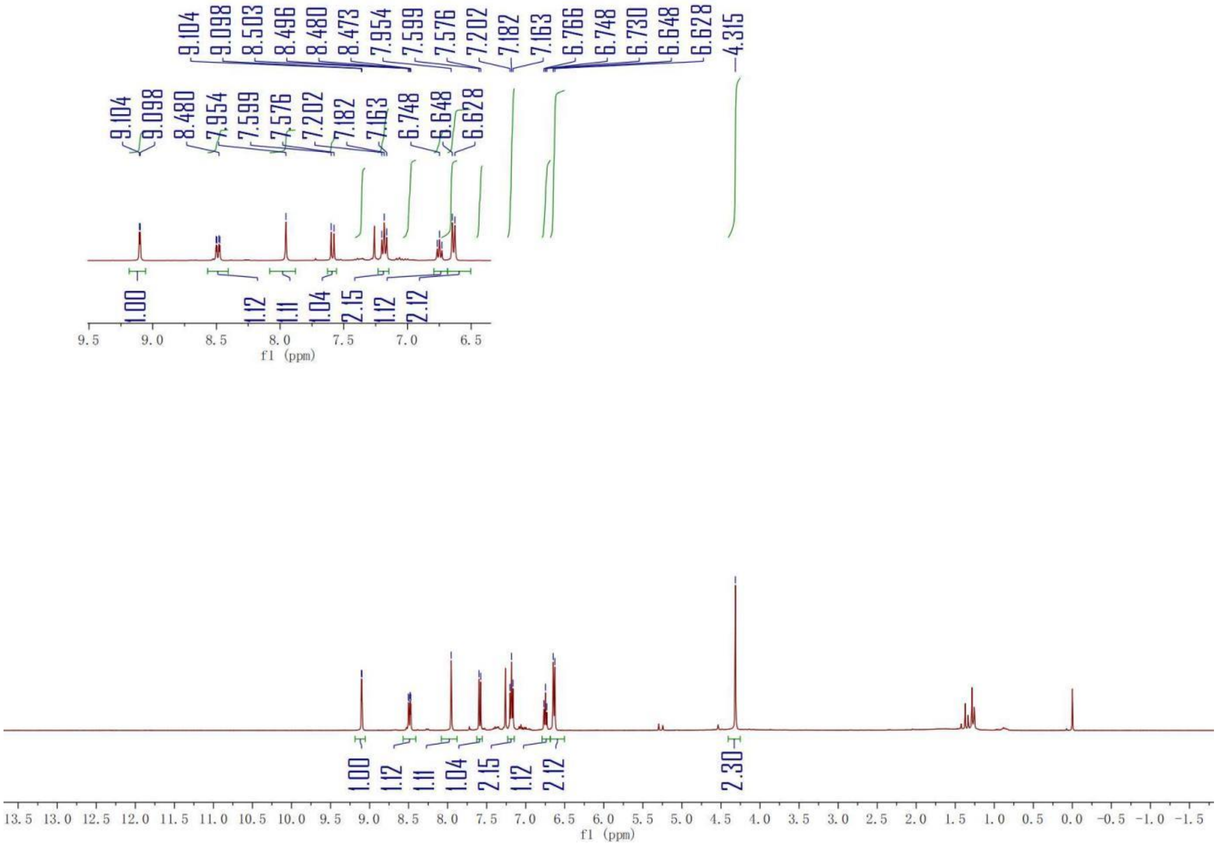

^{13}C NMR spectrum of compound 4l

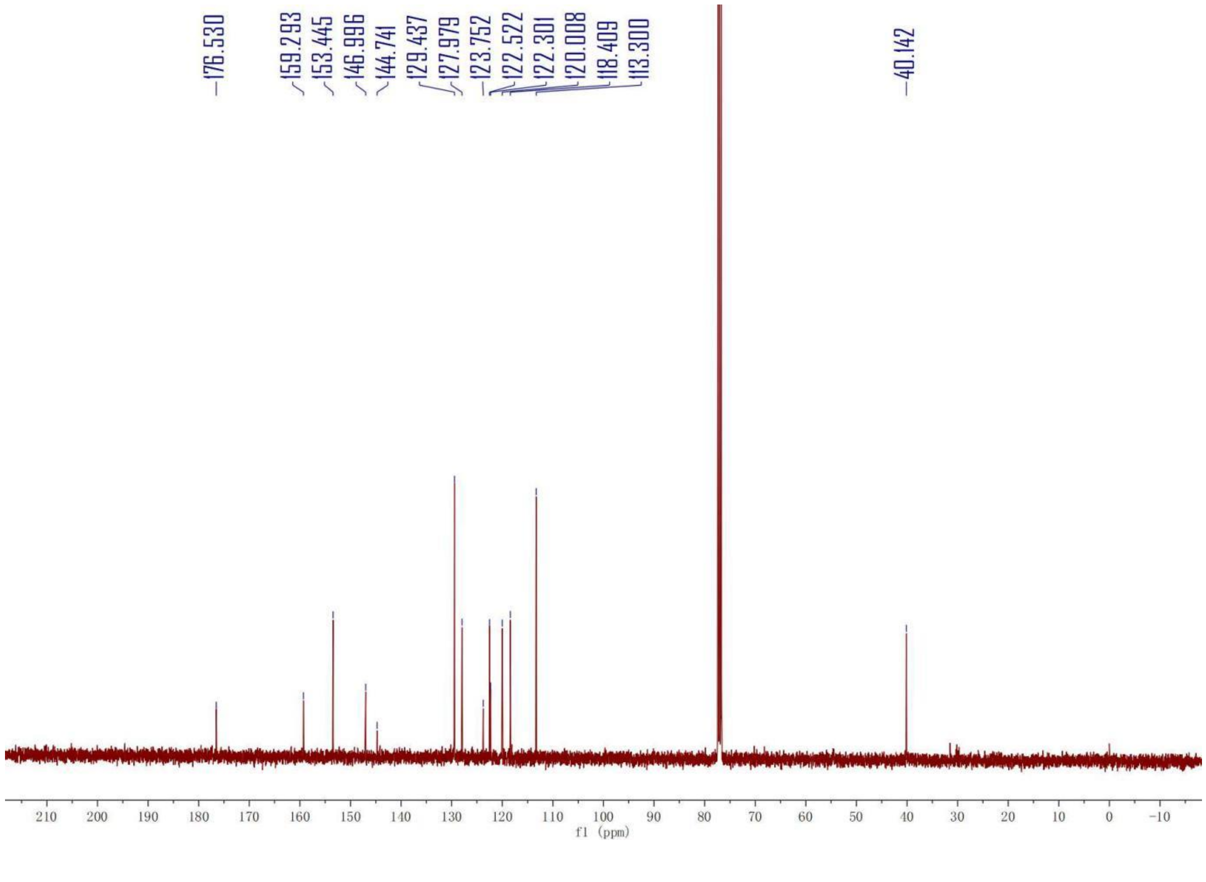

^{1}H NMR spectrum of compound 4m

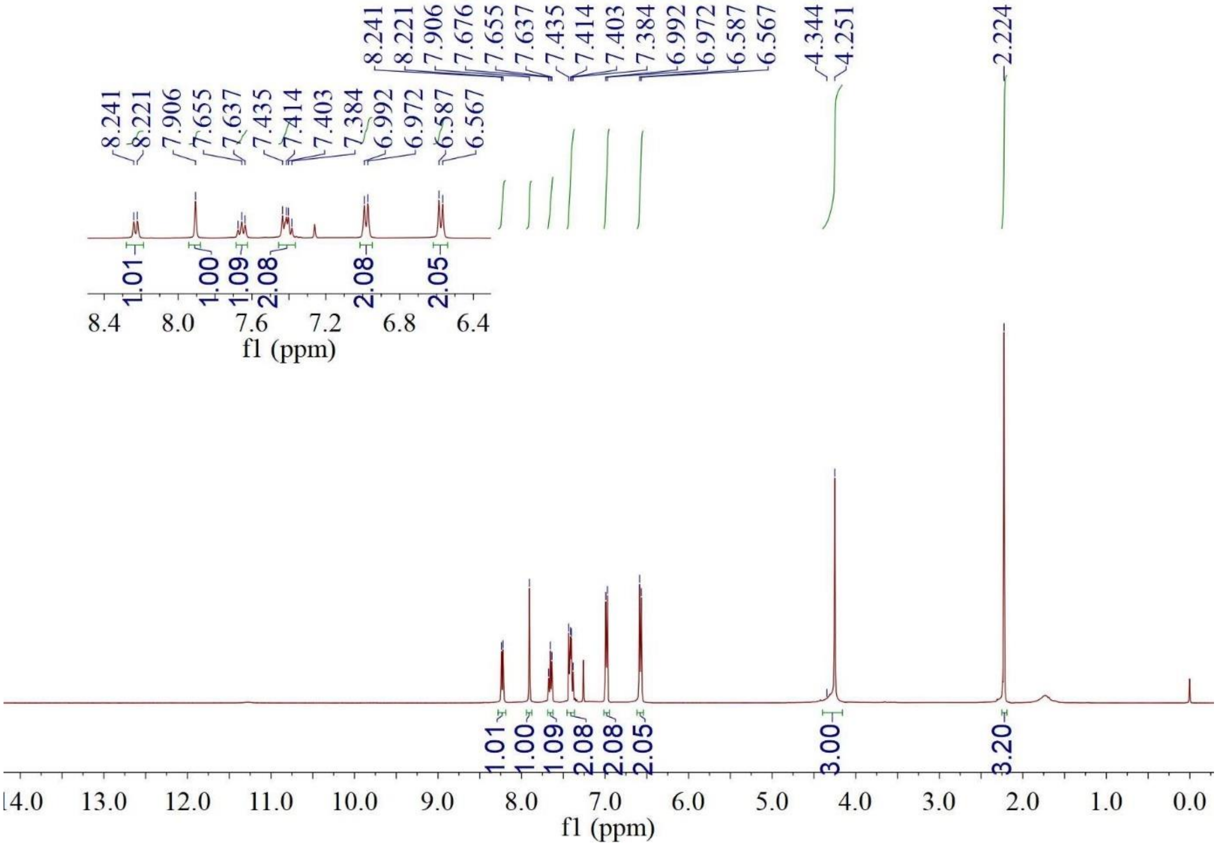

^{13}C NMR spectrum of compound 4m

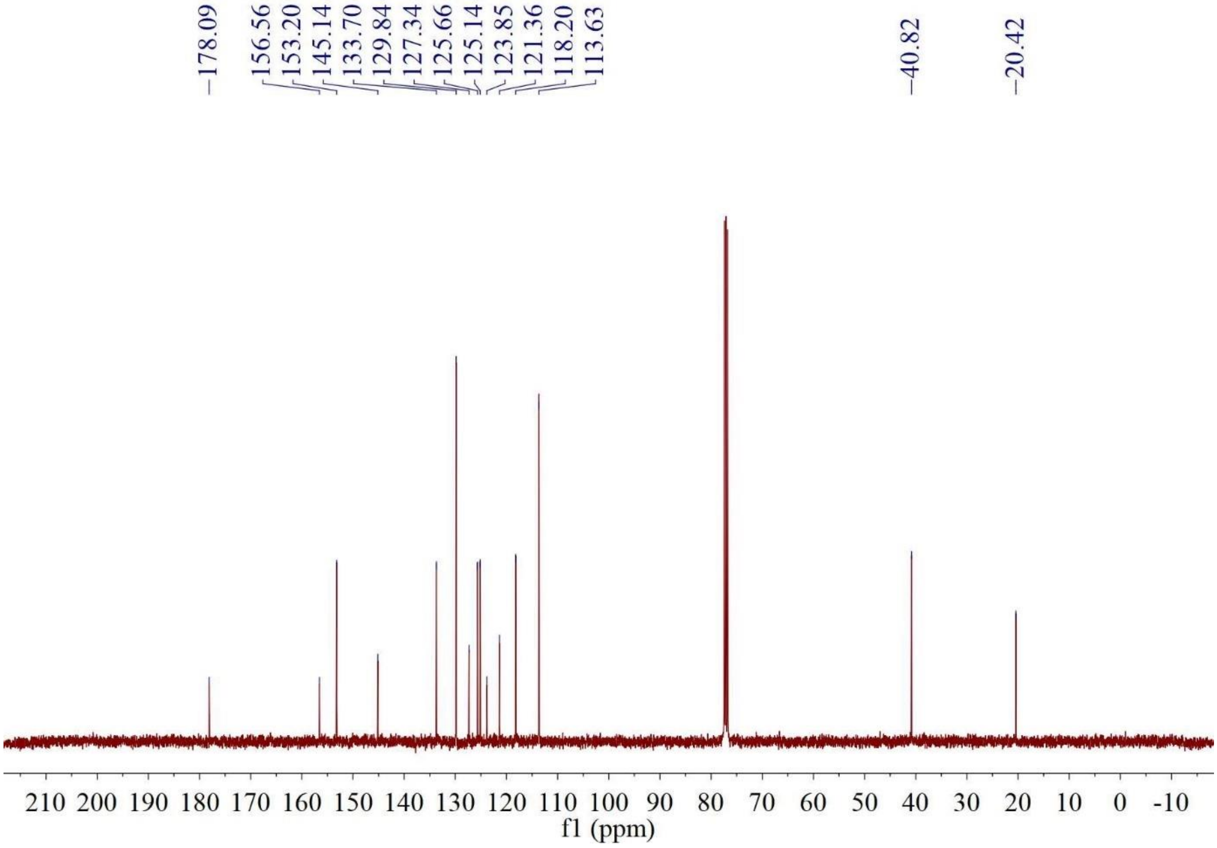

^{1}H NMR spectrum of compound 4n

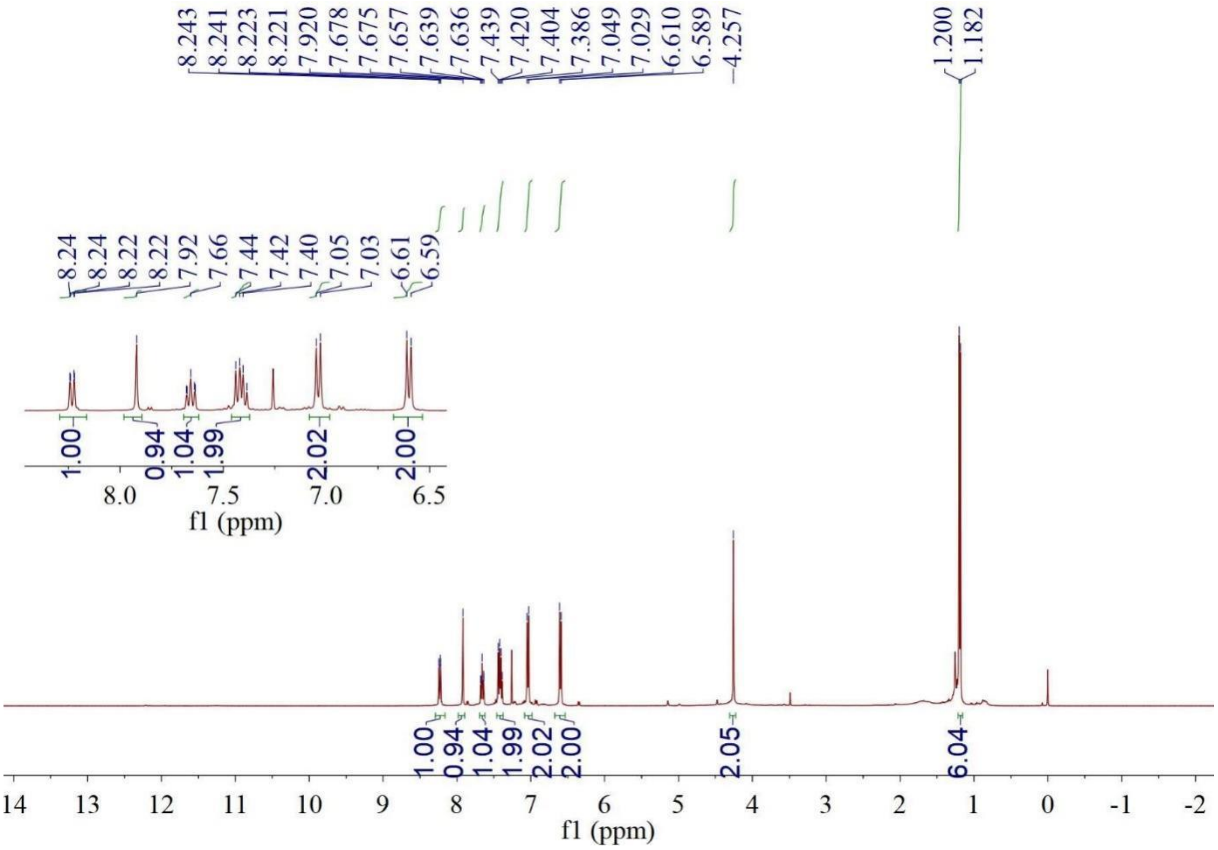

^{13}C NMR spectrum of compound 4n

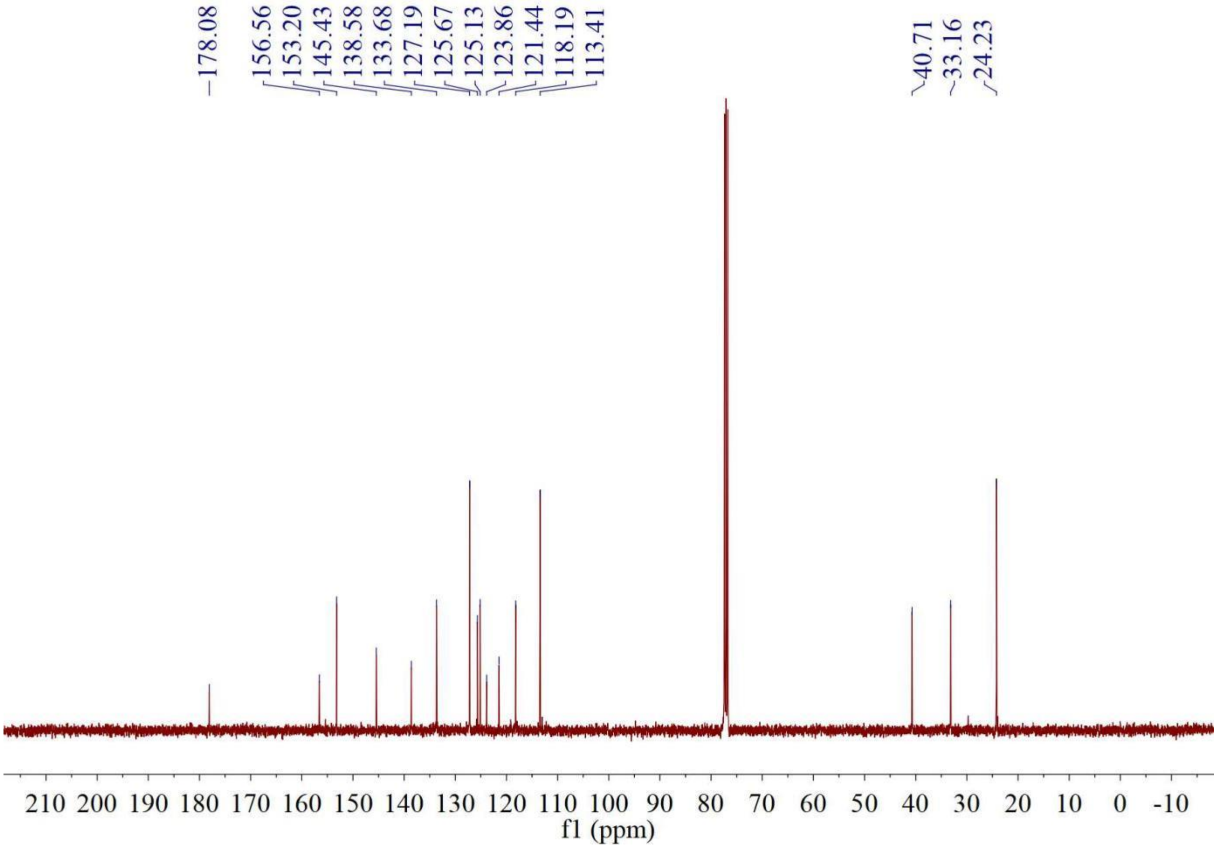

^{1}H NMR spectrum of compound 4o

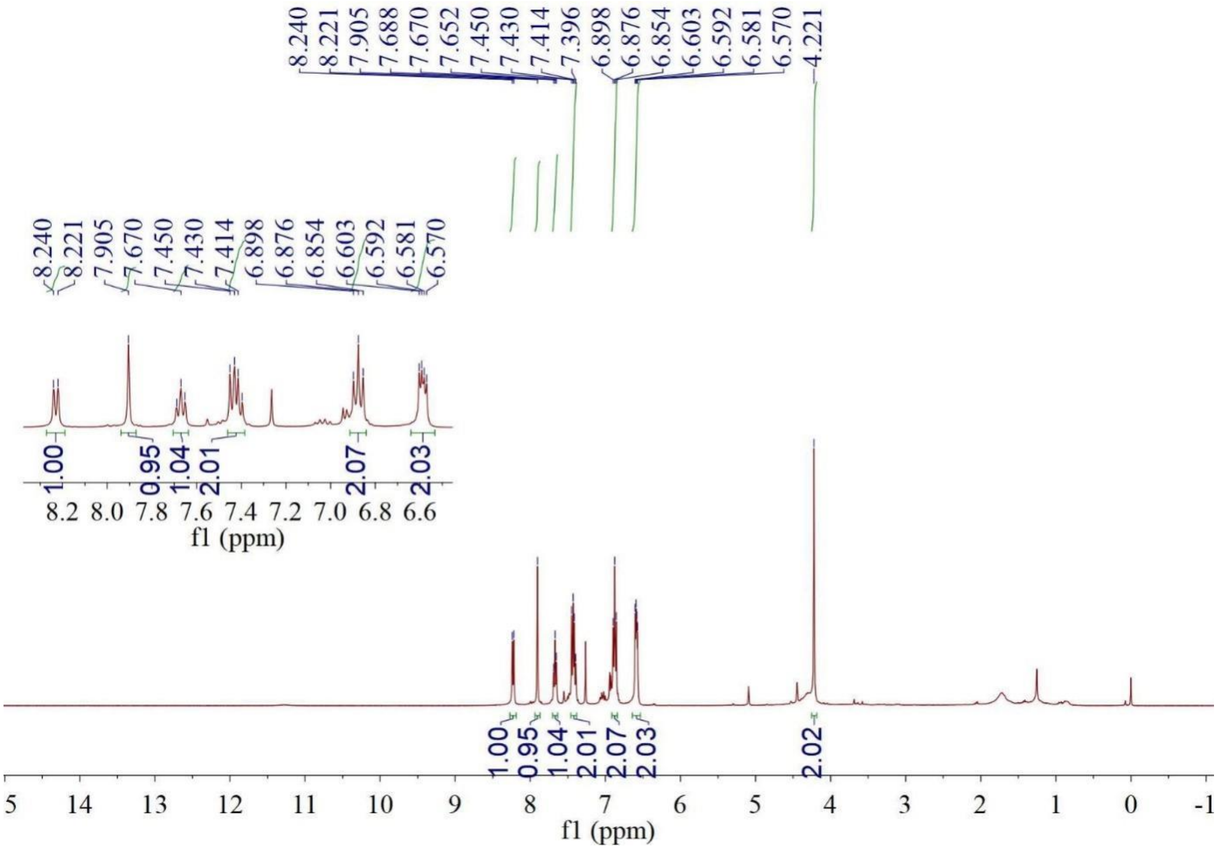

^{13}C NMR spectrum of compound 4o

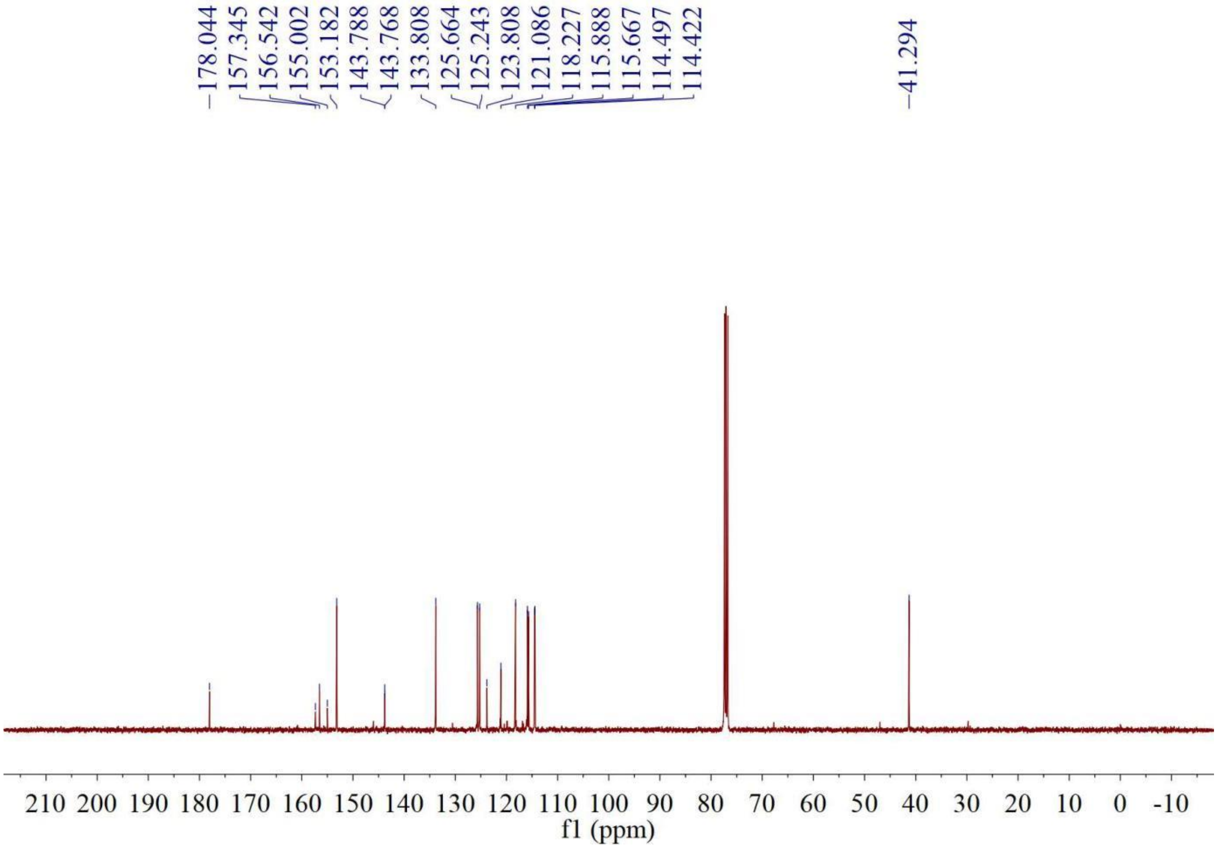

^{19}F NMR spectrum of compound 4o

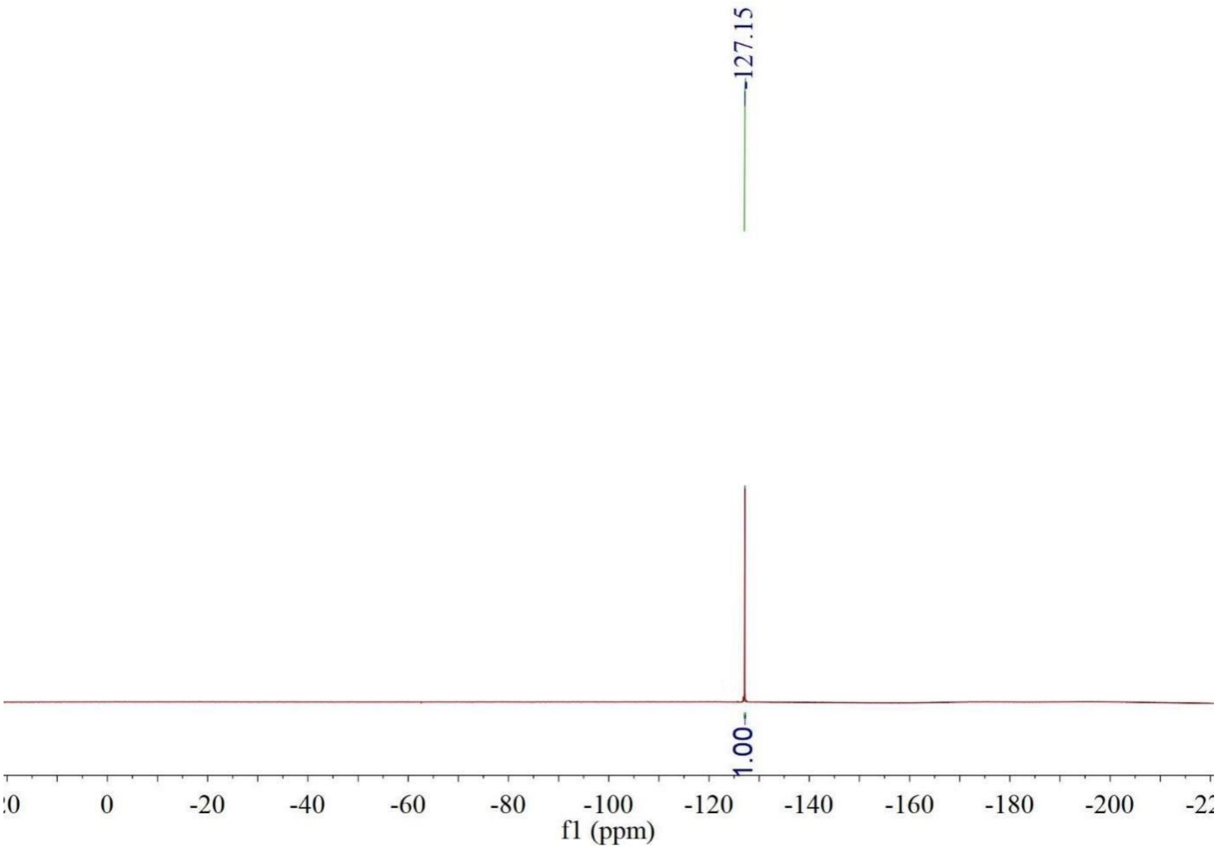

^{1}H NMR spectrum of compound 4p

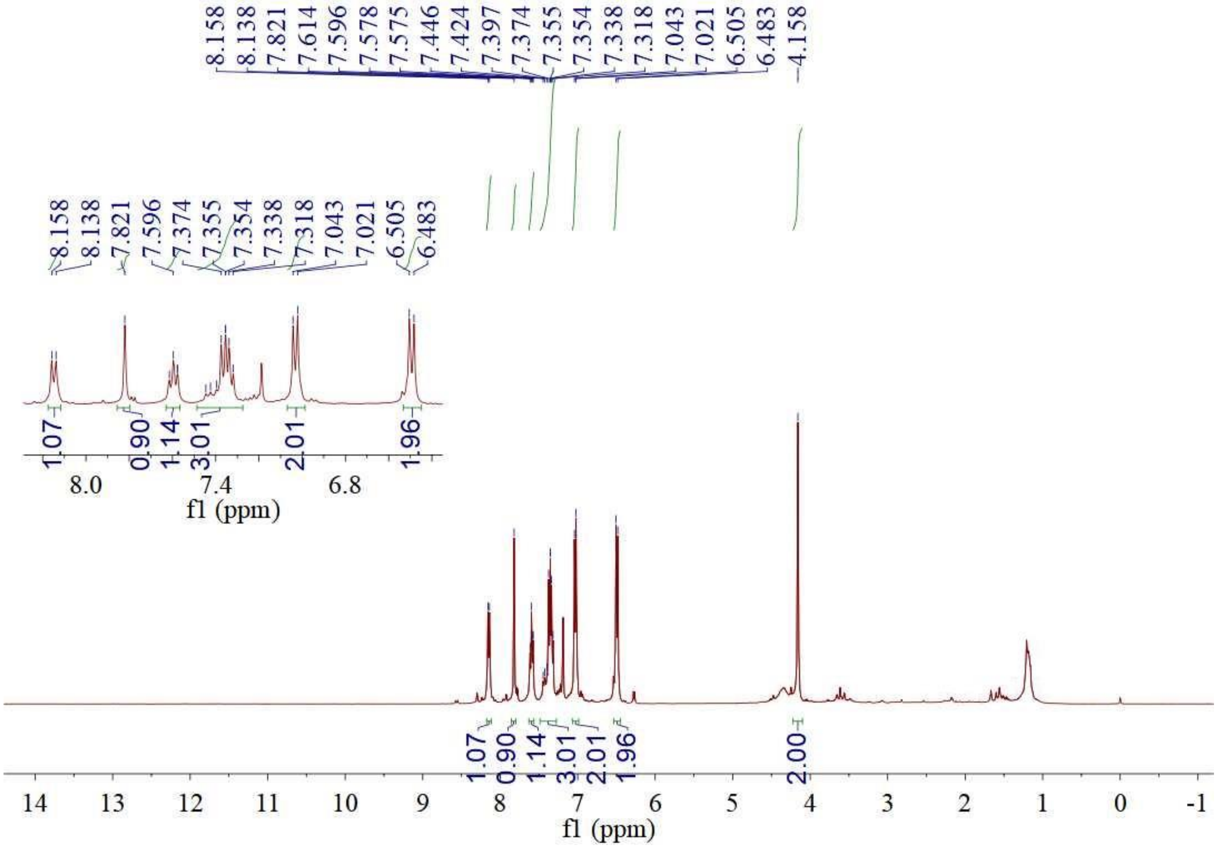

^{13}C NMR spectrum of compound 4p

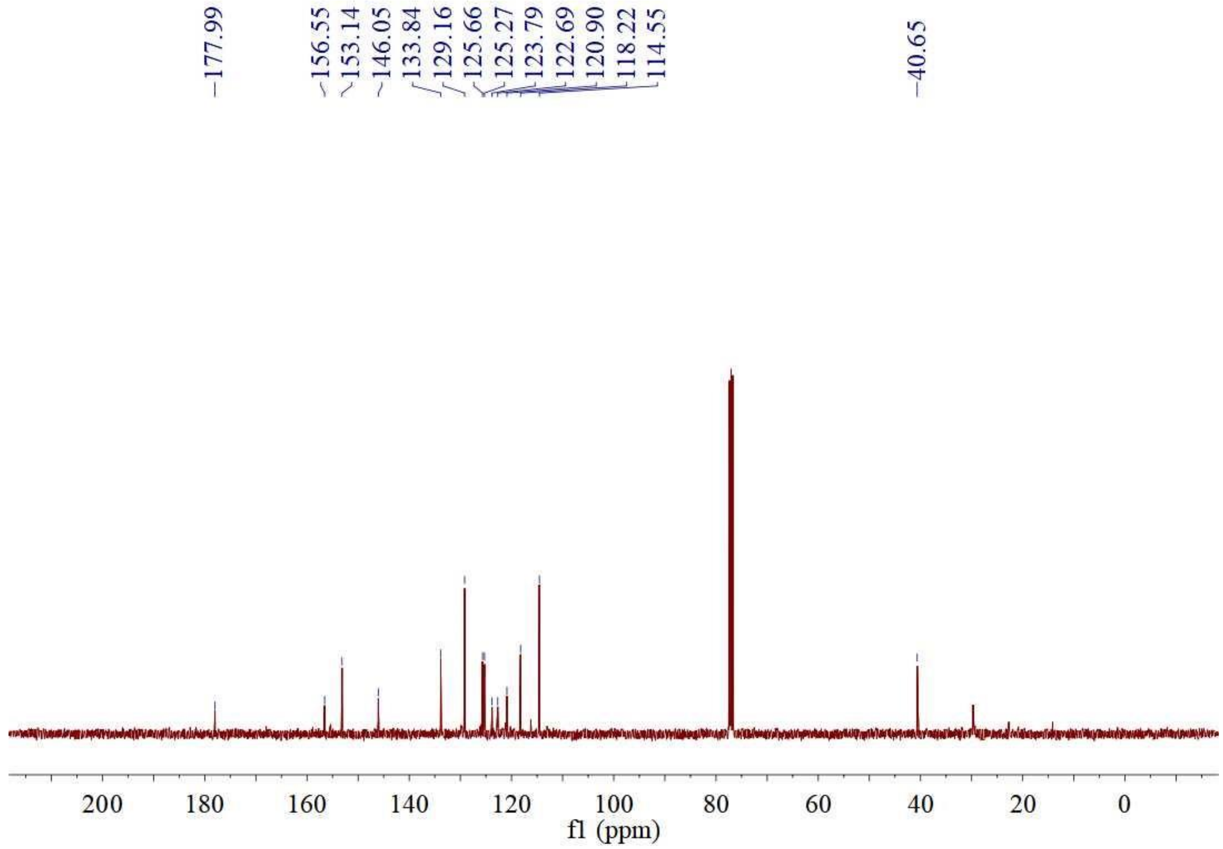

^{1}H NMR spectrum of compound 4q

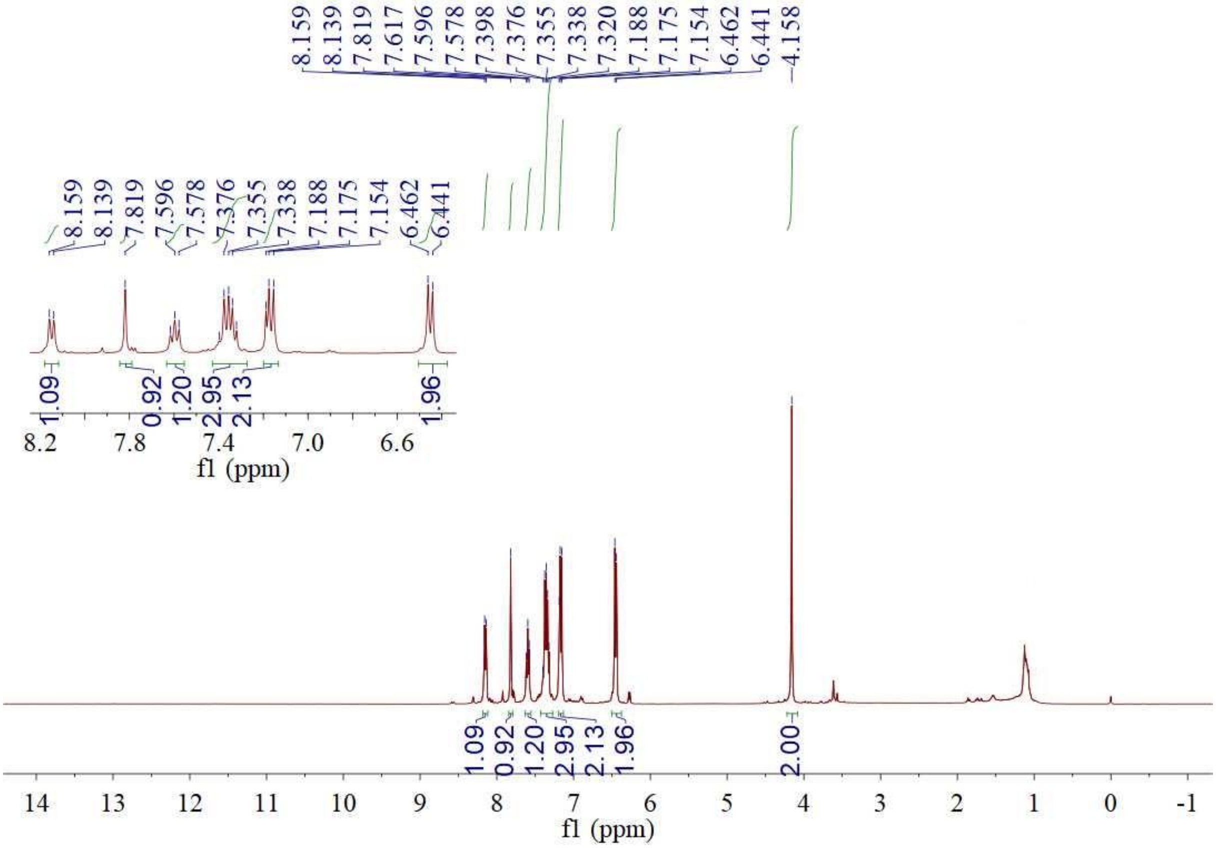

^{13}C NMR spectrum of compound 4q

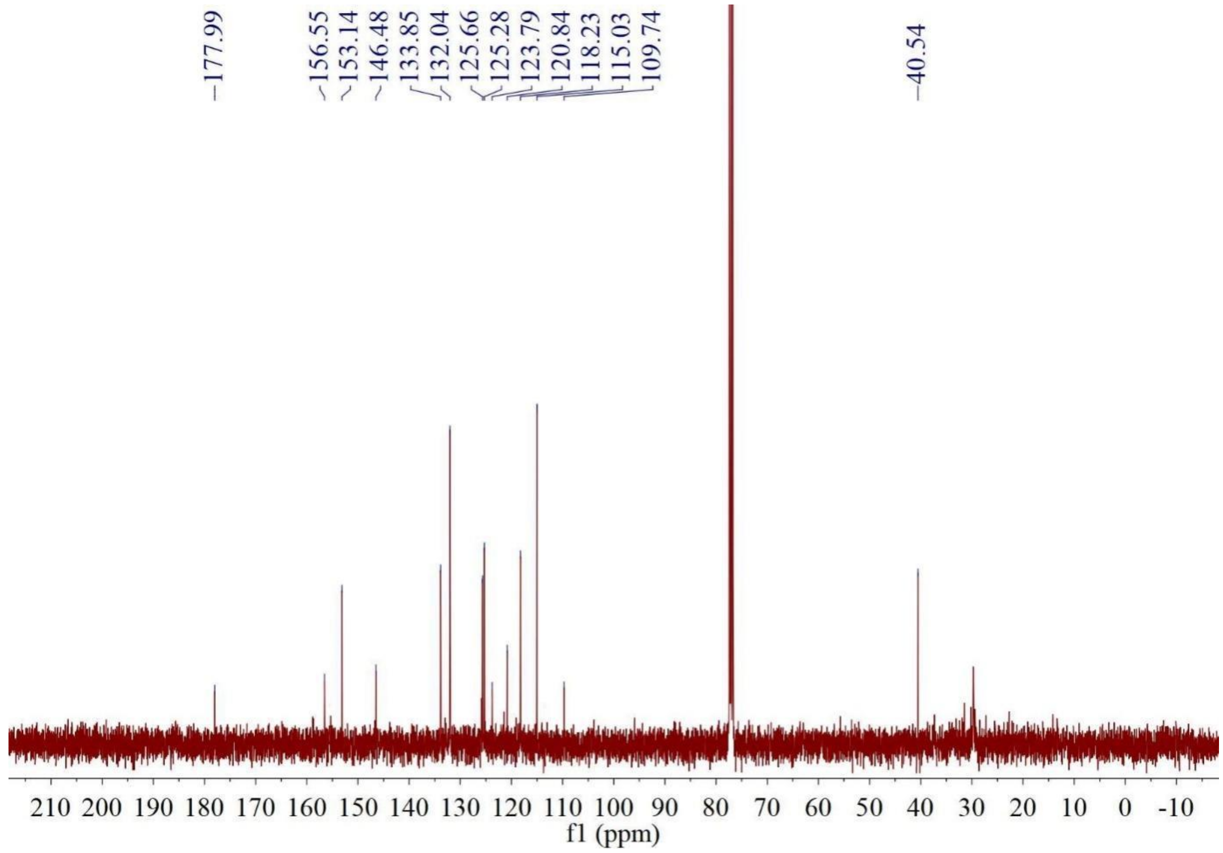

^{1}H NMR spectrum of compound 4r

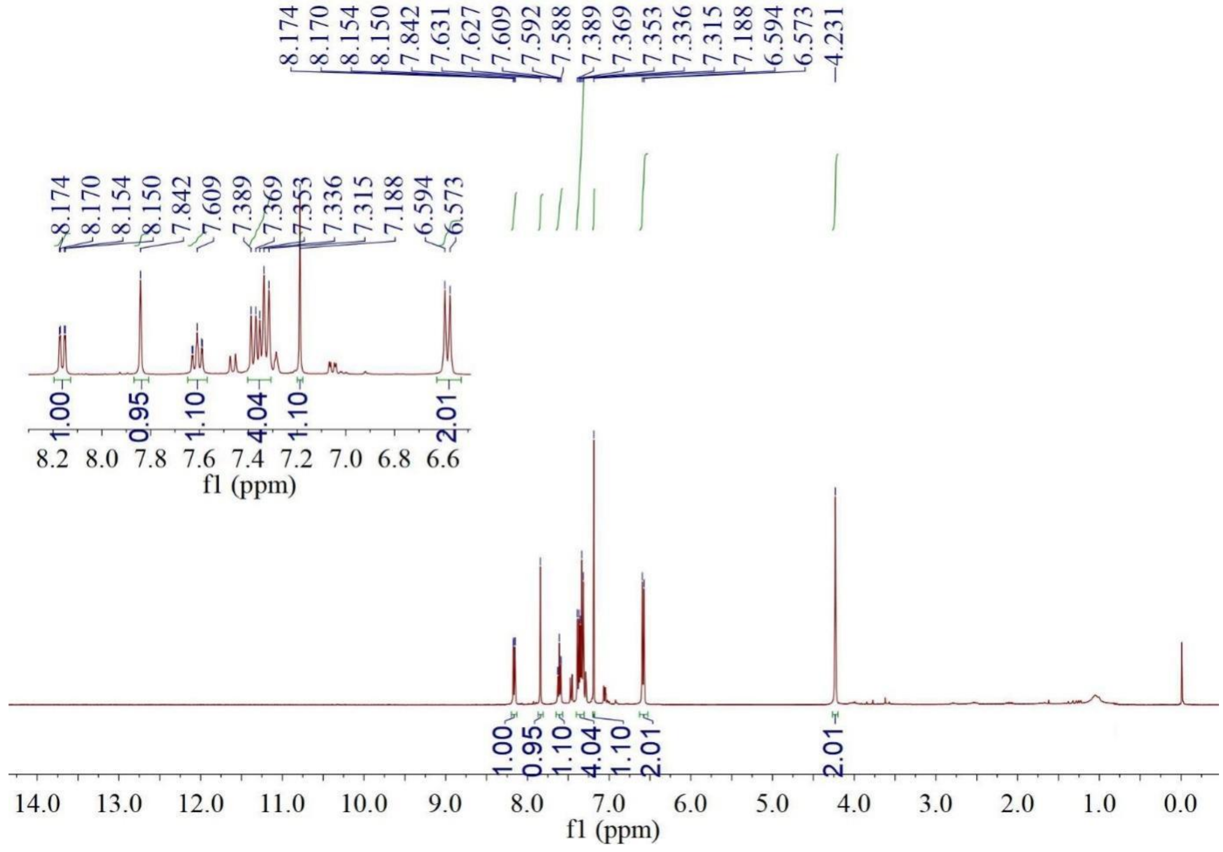

^{13}C NMR spectrum of compound 4r

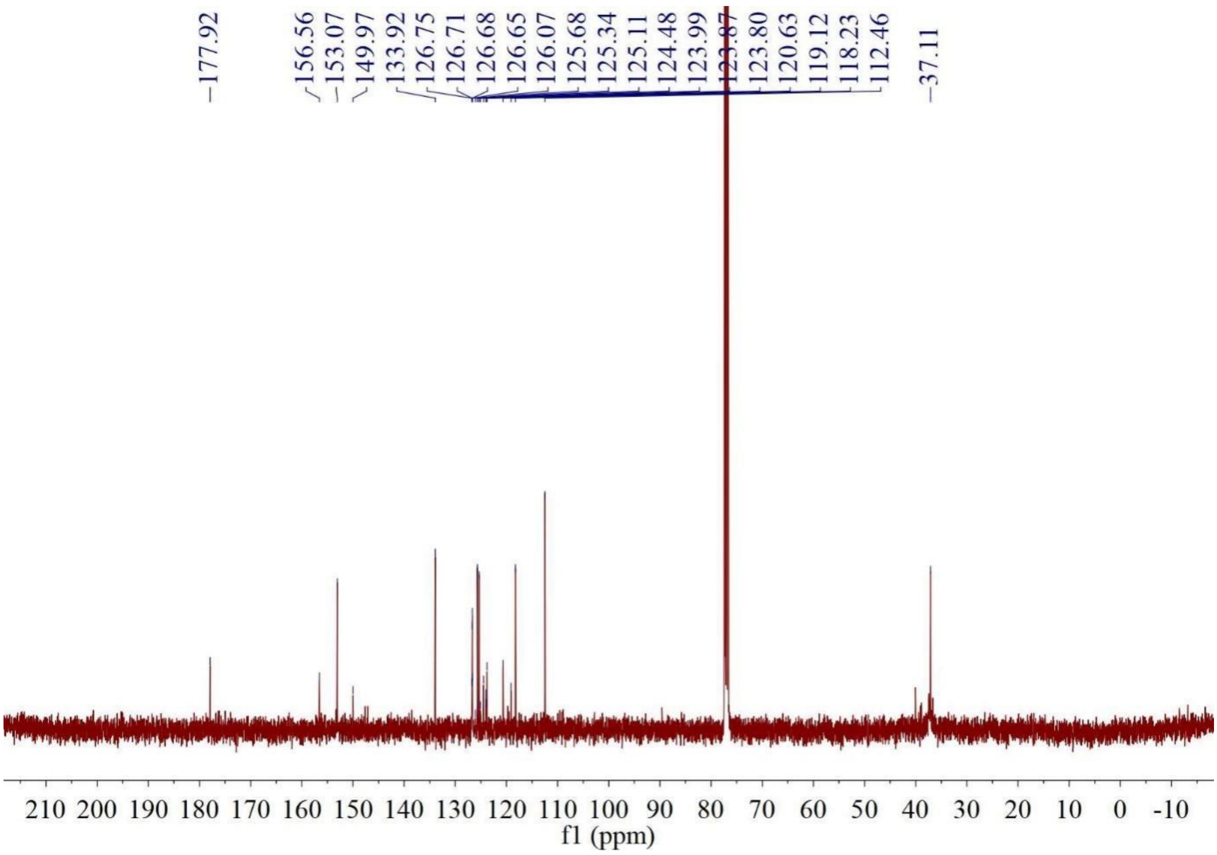

^{19}F NMR spectrum of compound 4r

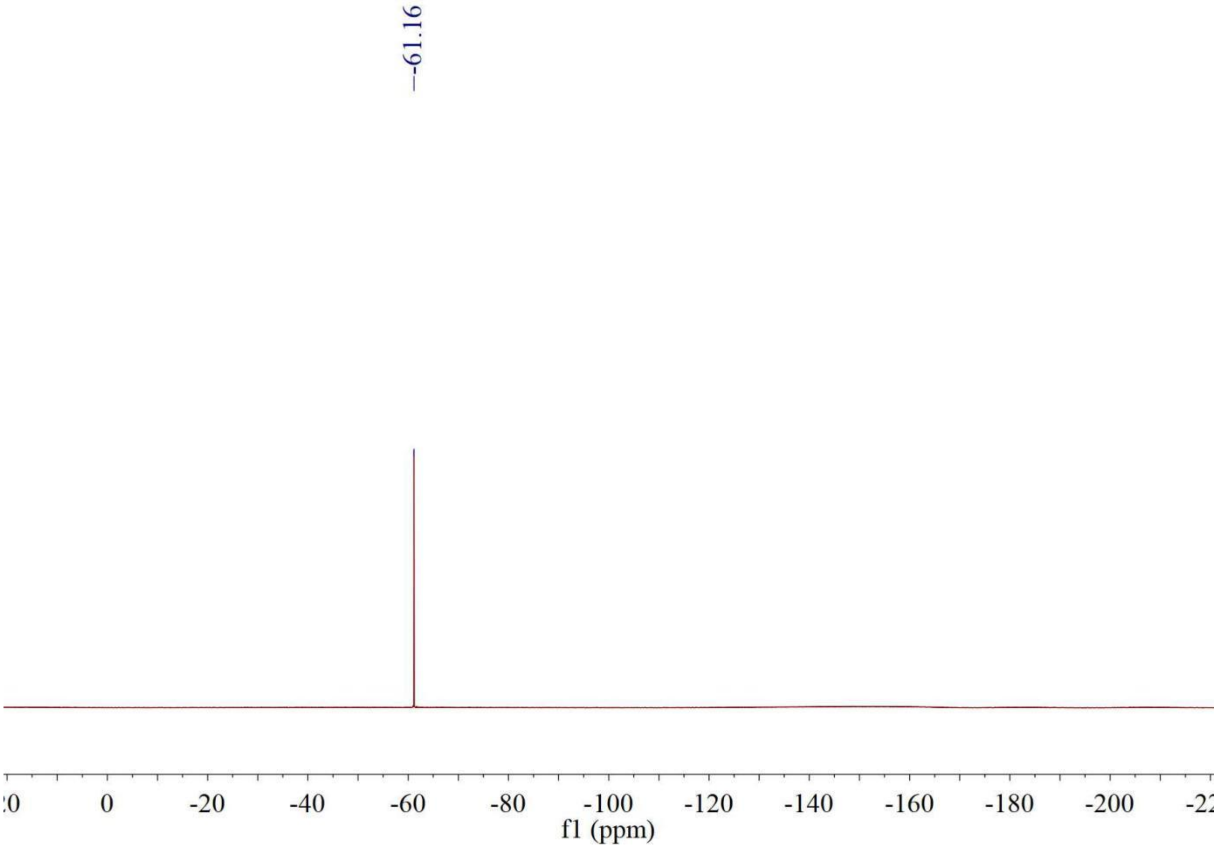

^{1}H NMR spectrum of compound 4s

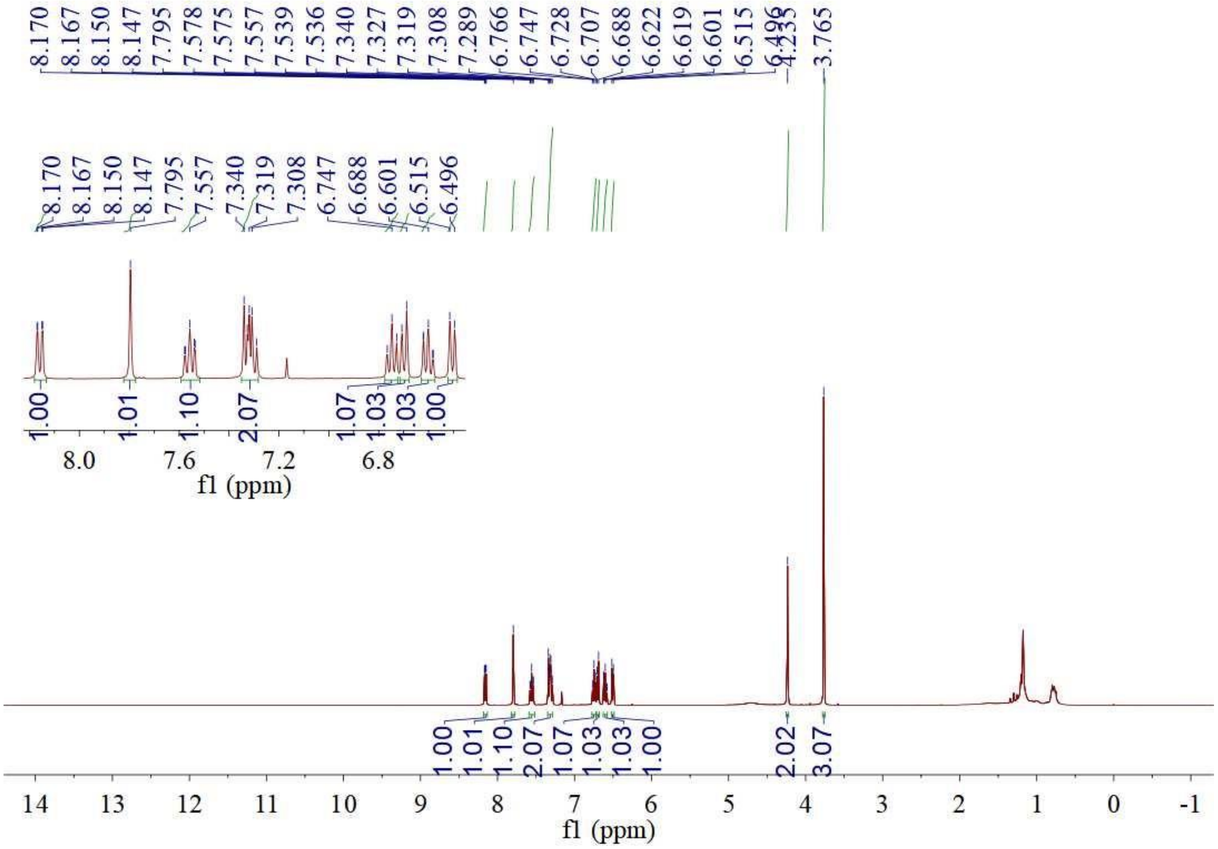

^{13}C NMR spectrum of compound 4s

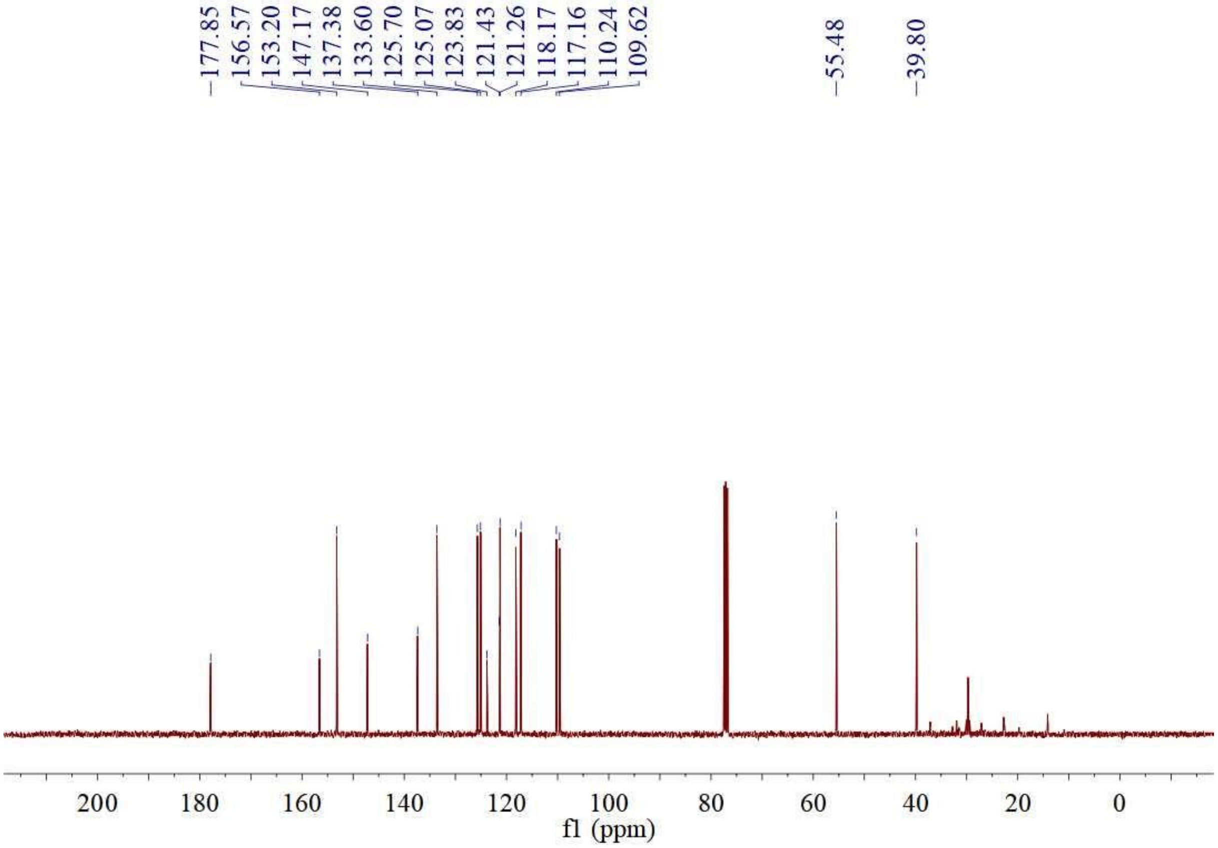

^{1}H NMR spectrum of compound 4t

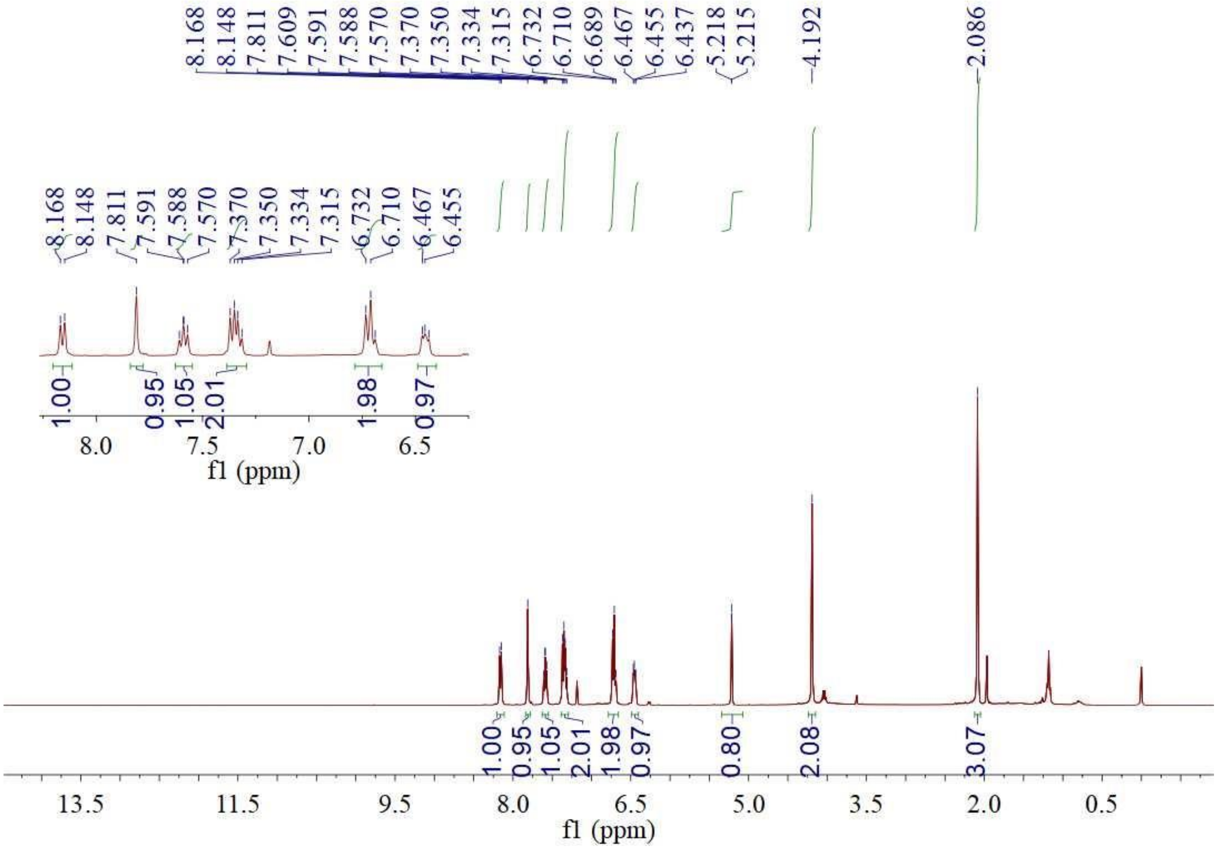

^{13}C NMR spectrum of compound 4t

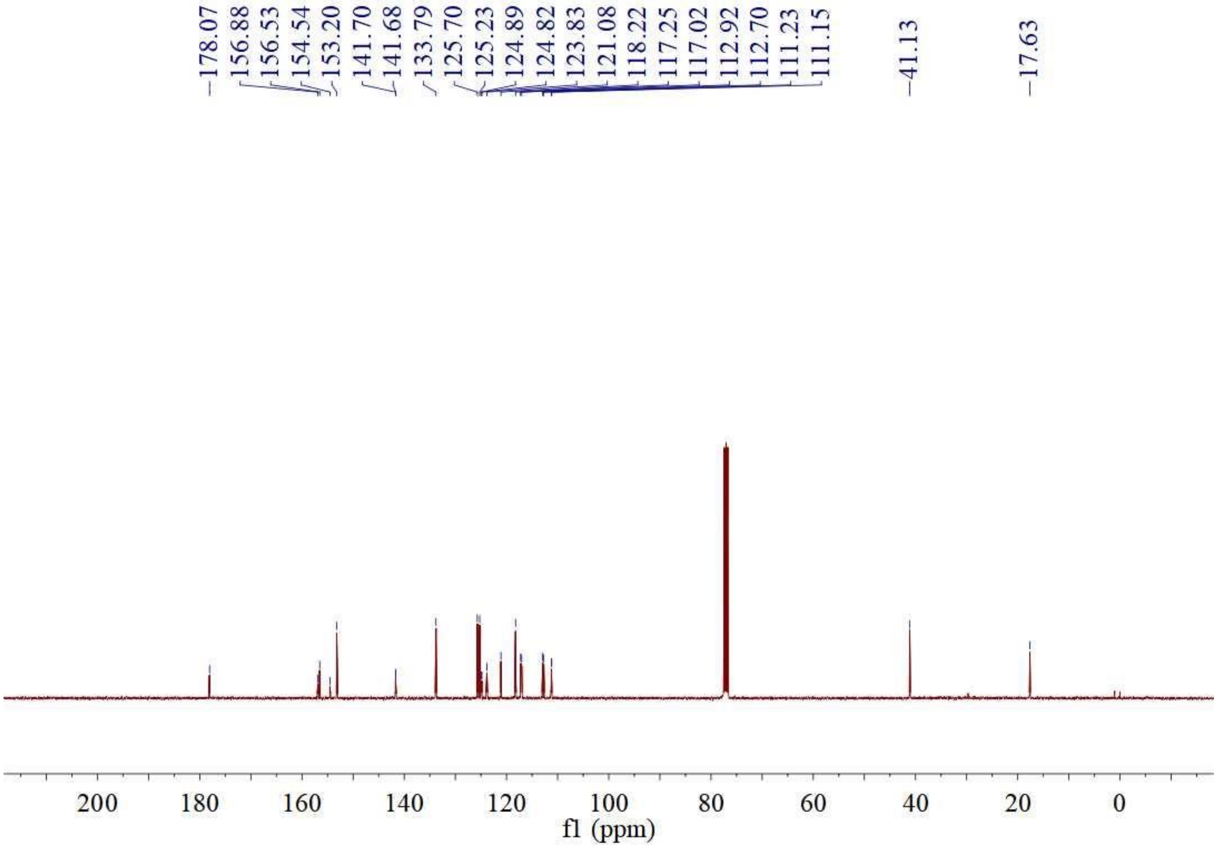

^{19}F NMR spectrum of compound 4t

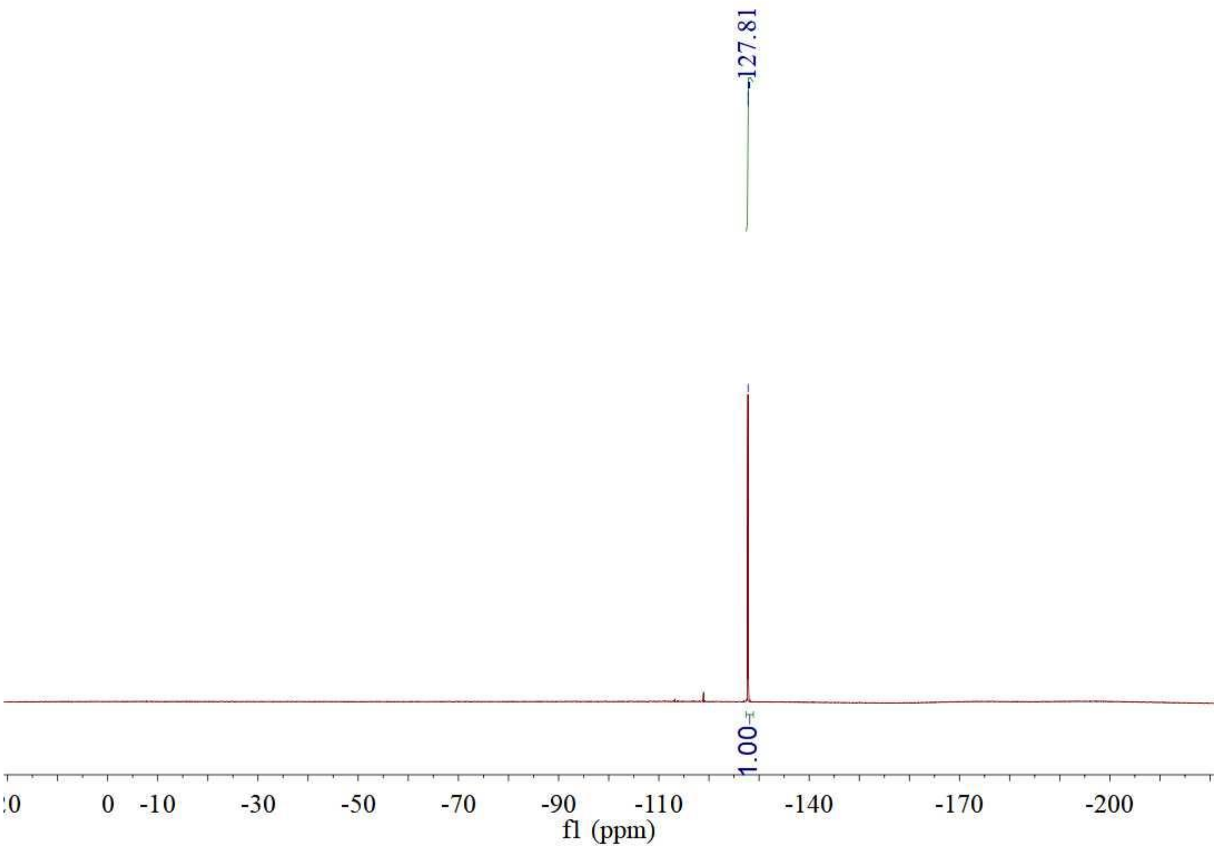

^{1}H NMR spectrum of compound 4u

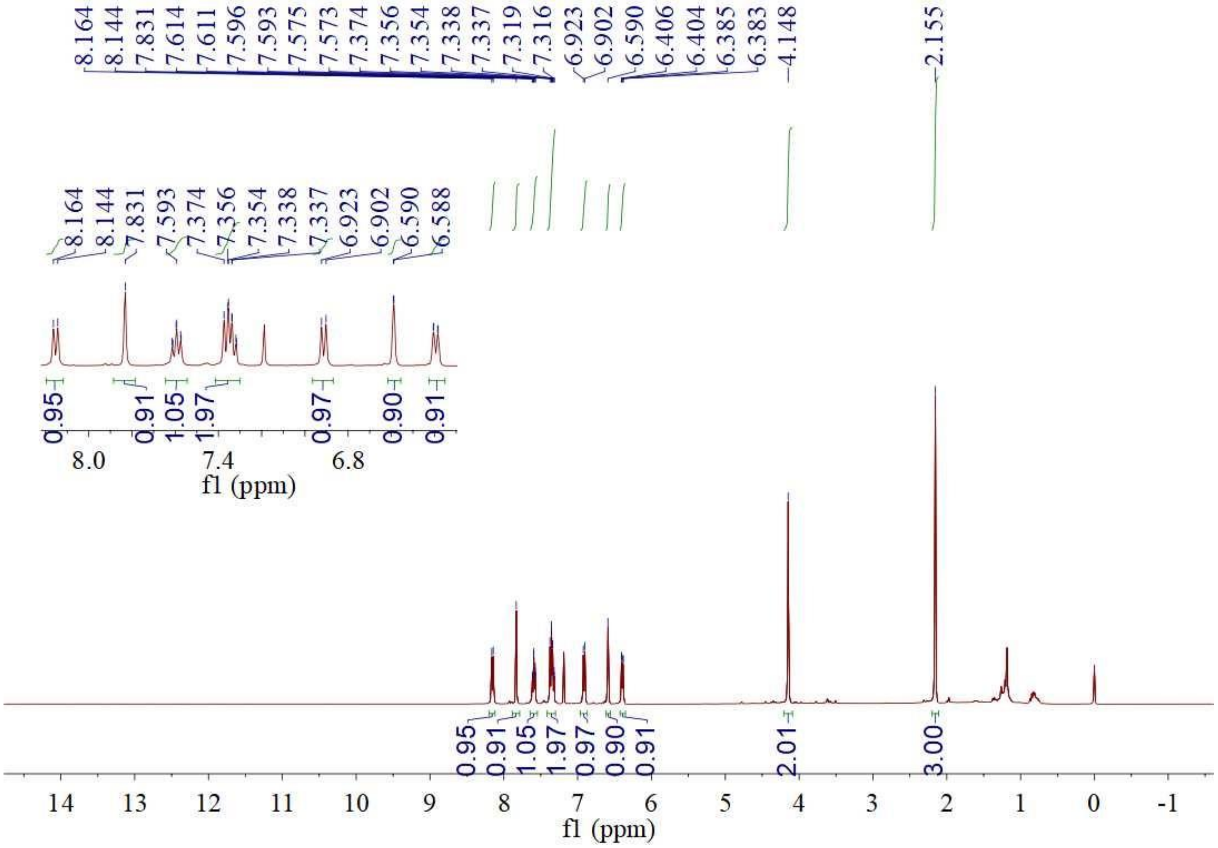

^{13}C NMR spectrum of compound 4u

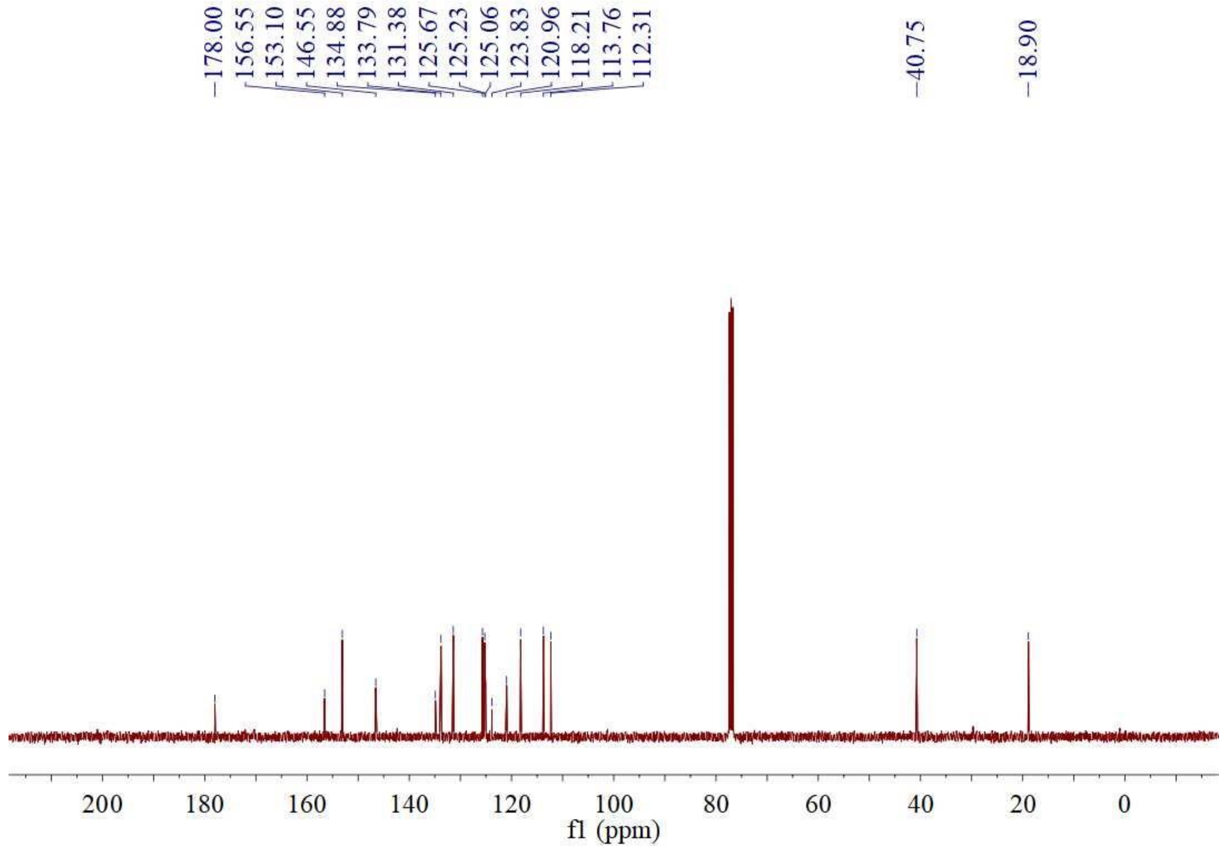

^{1}H NMR spectrum of compound 4v

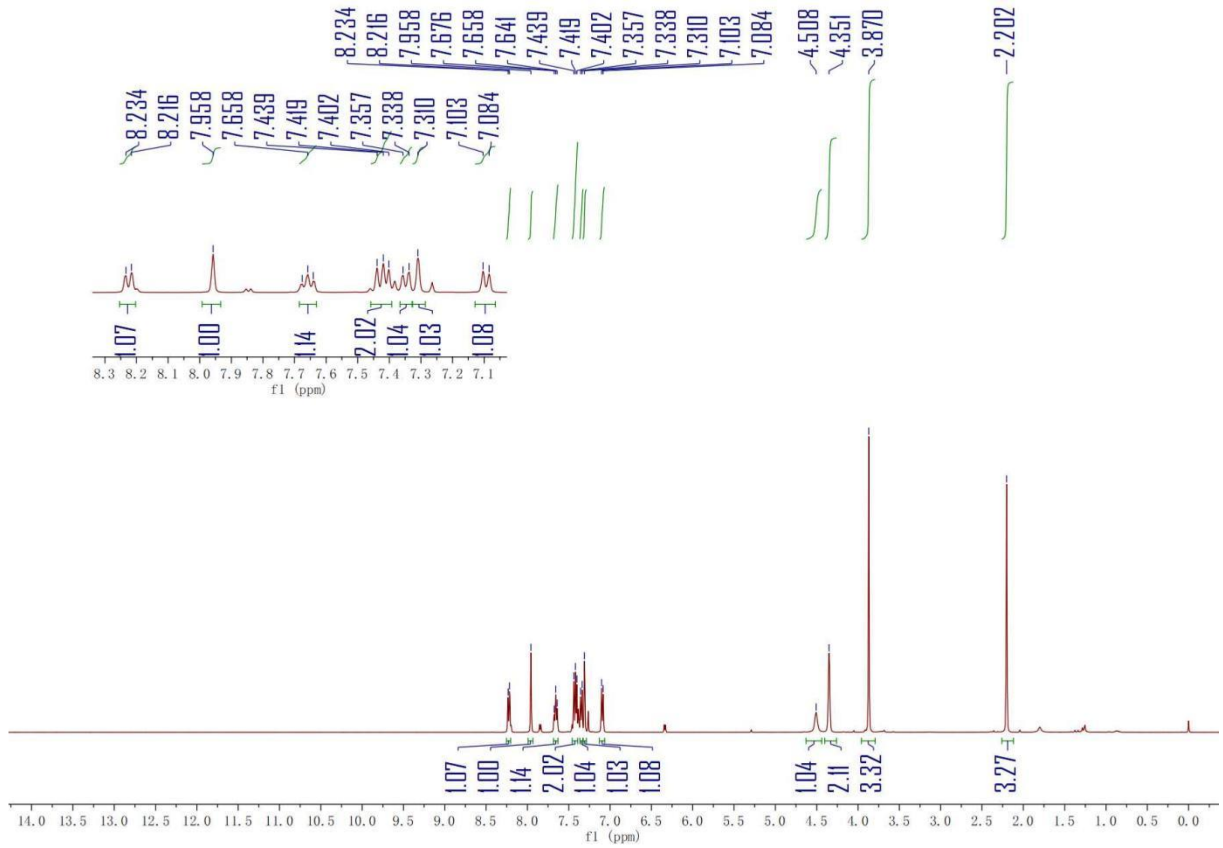

^{13}C NMR spectrum of compound 4v

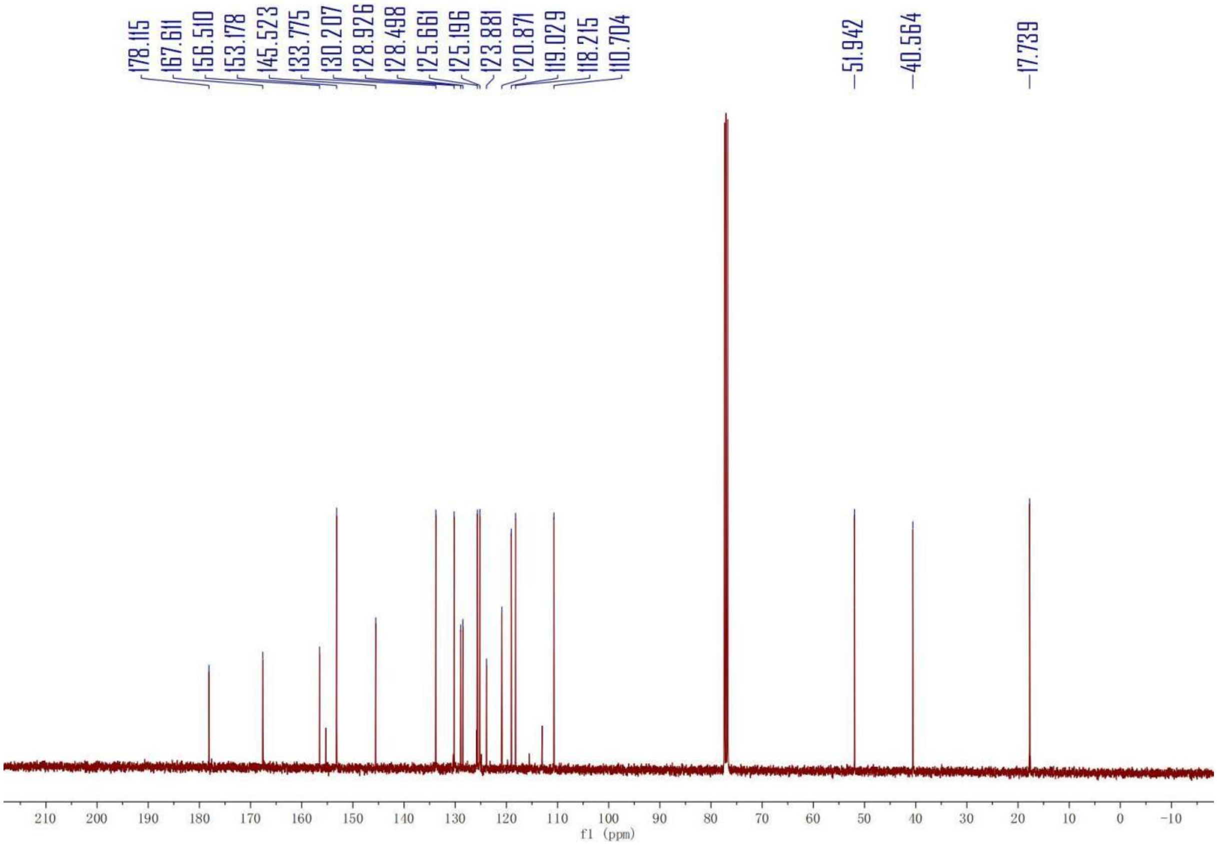

^{1}H NMR spectrum of compound 4w

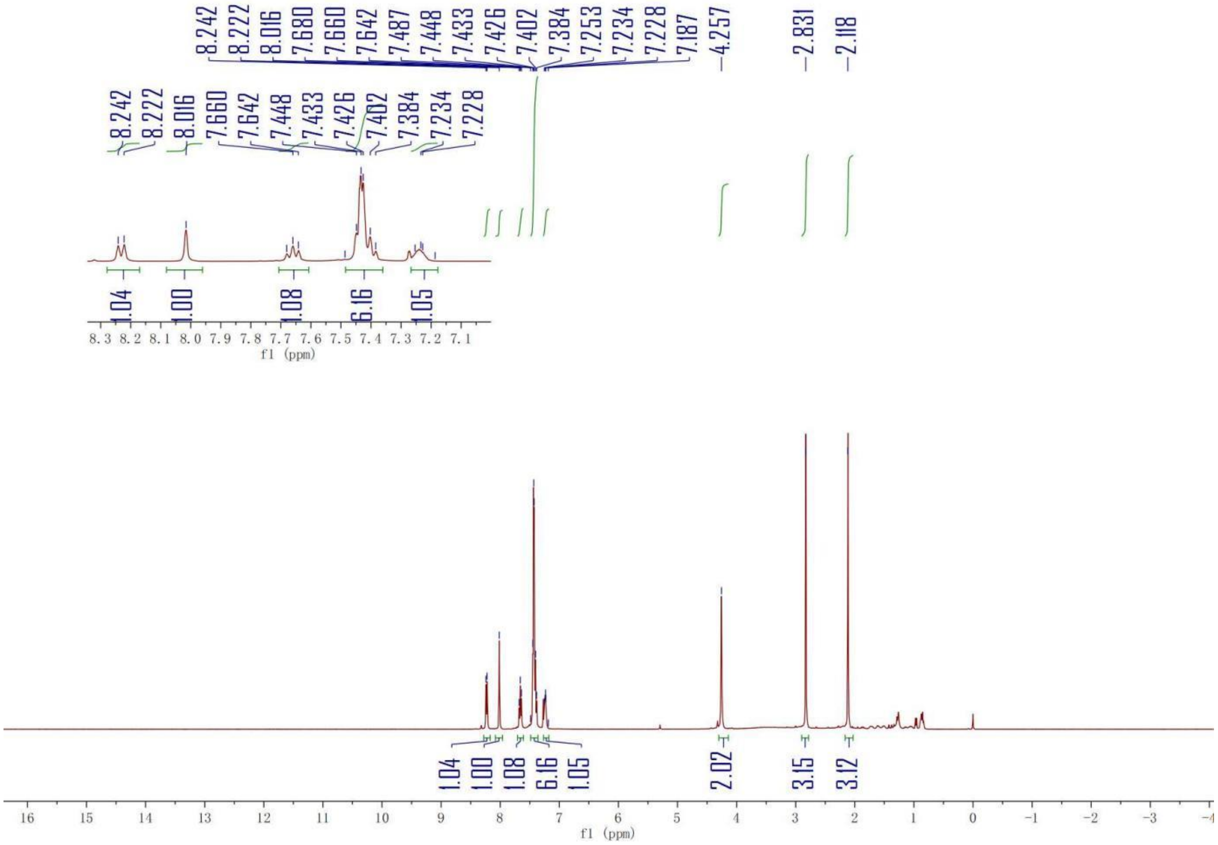

^{13}C NMR spectrum of compound 4w

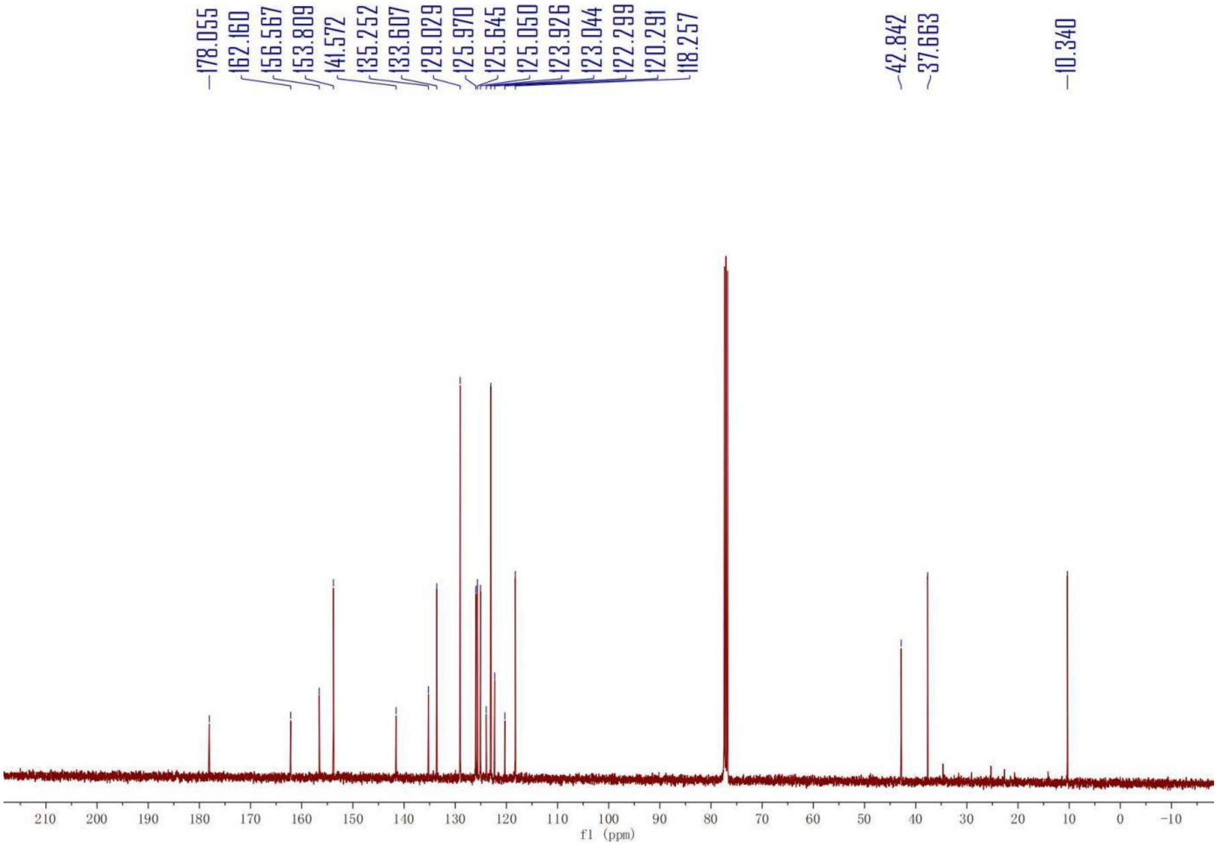

^{1}H NMR spectrum of compound 4x

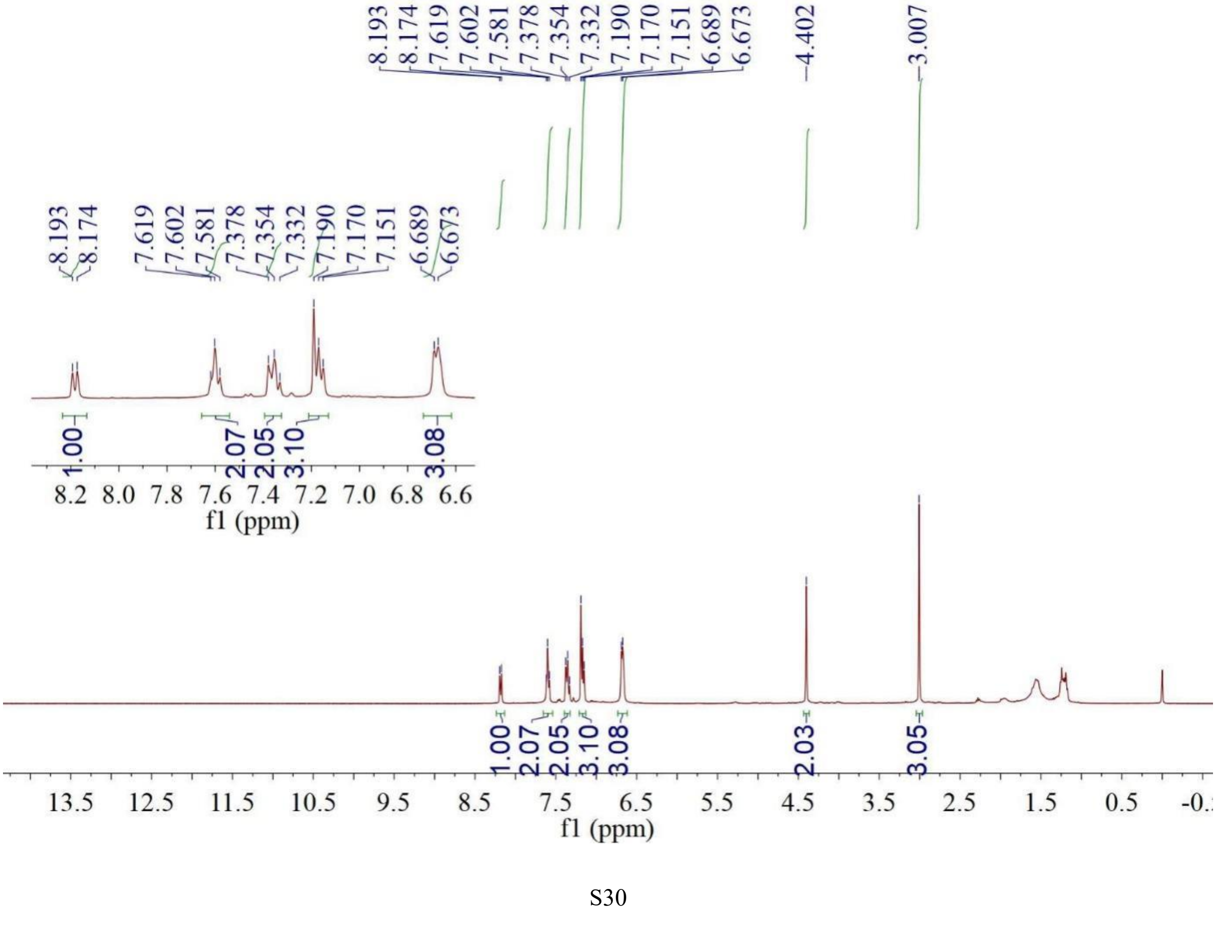

^{13}C NMR spectrum of compound 4x

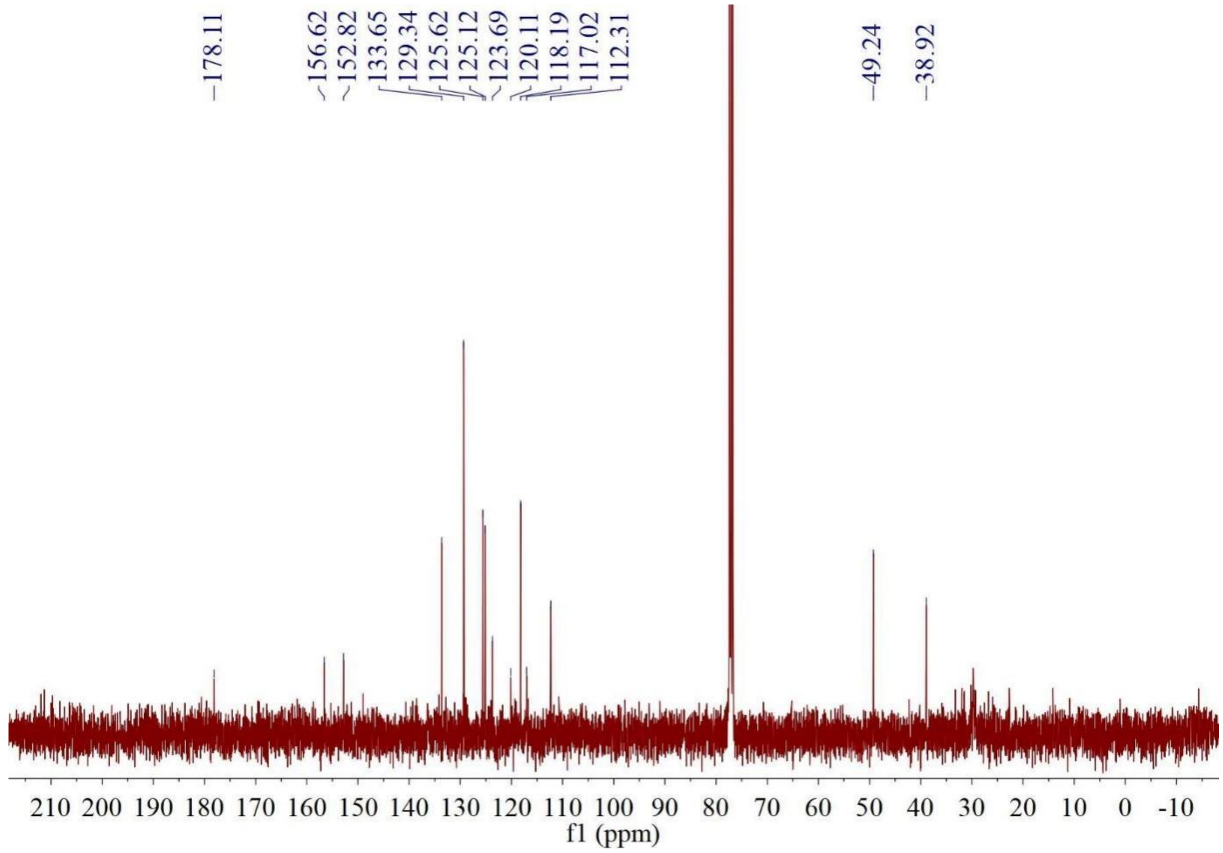

^{1}H NMR spectrum of compound 4y

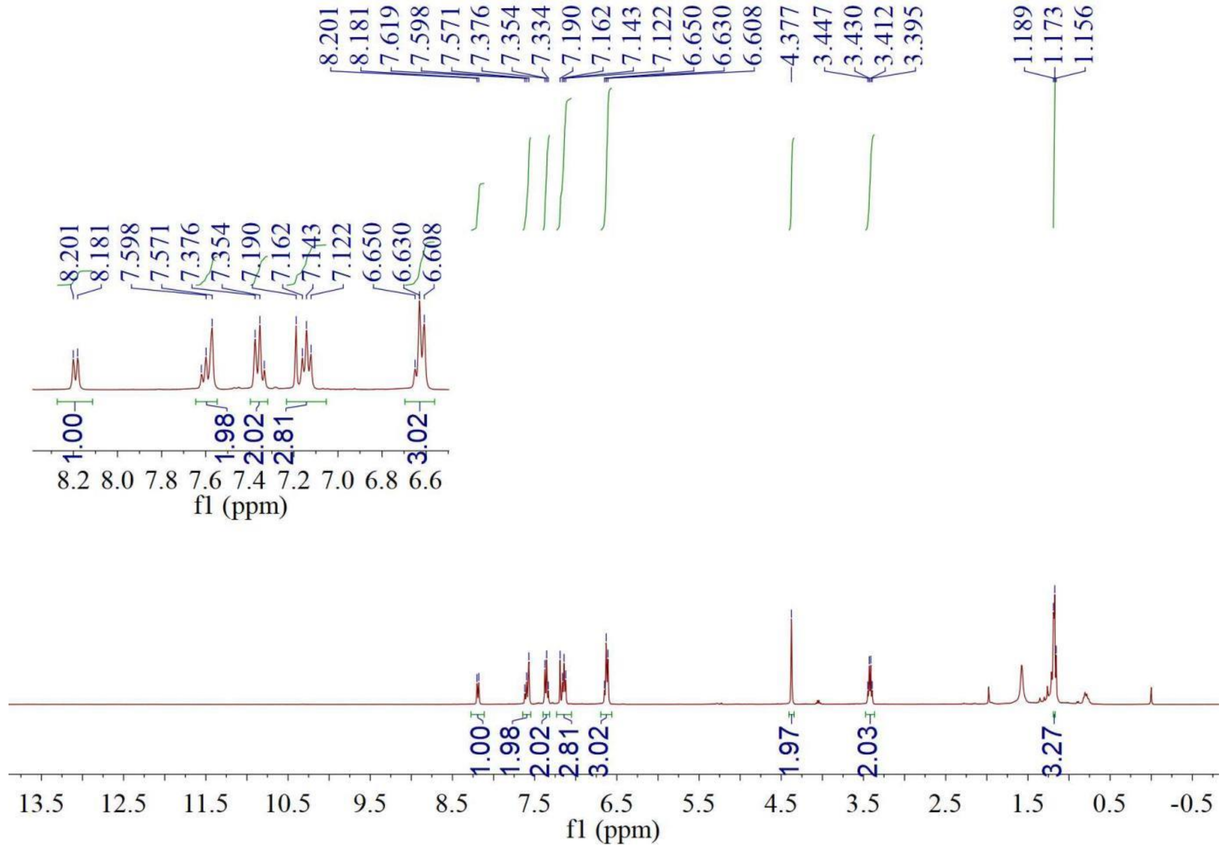

^{13}C NMR spectrum of compound 4y

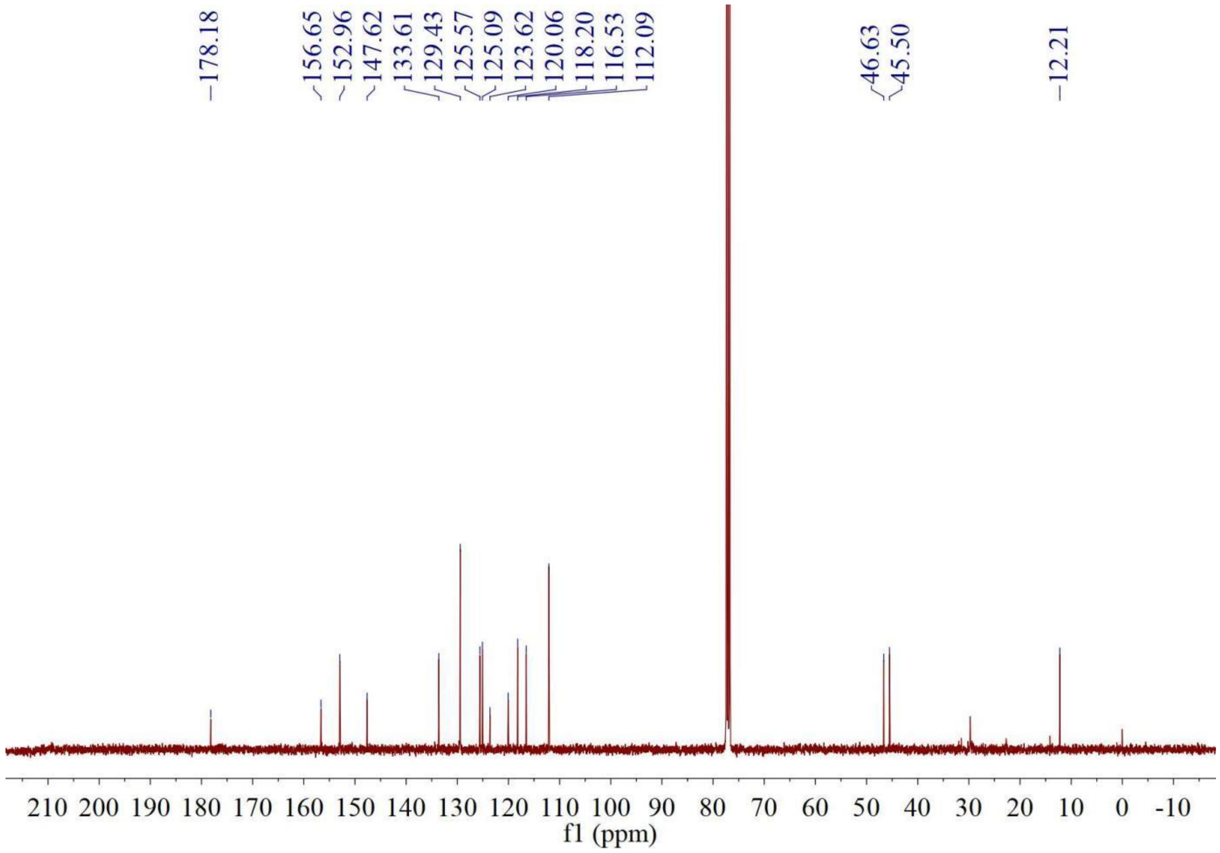

^{1}H NMR spectrum of compound 4z

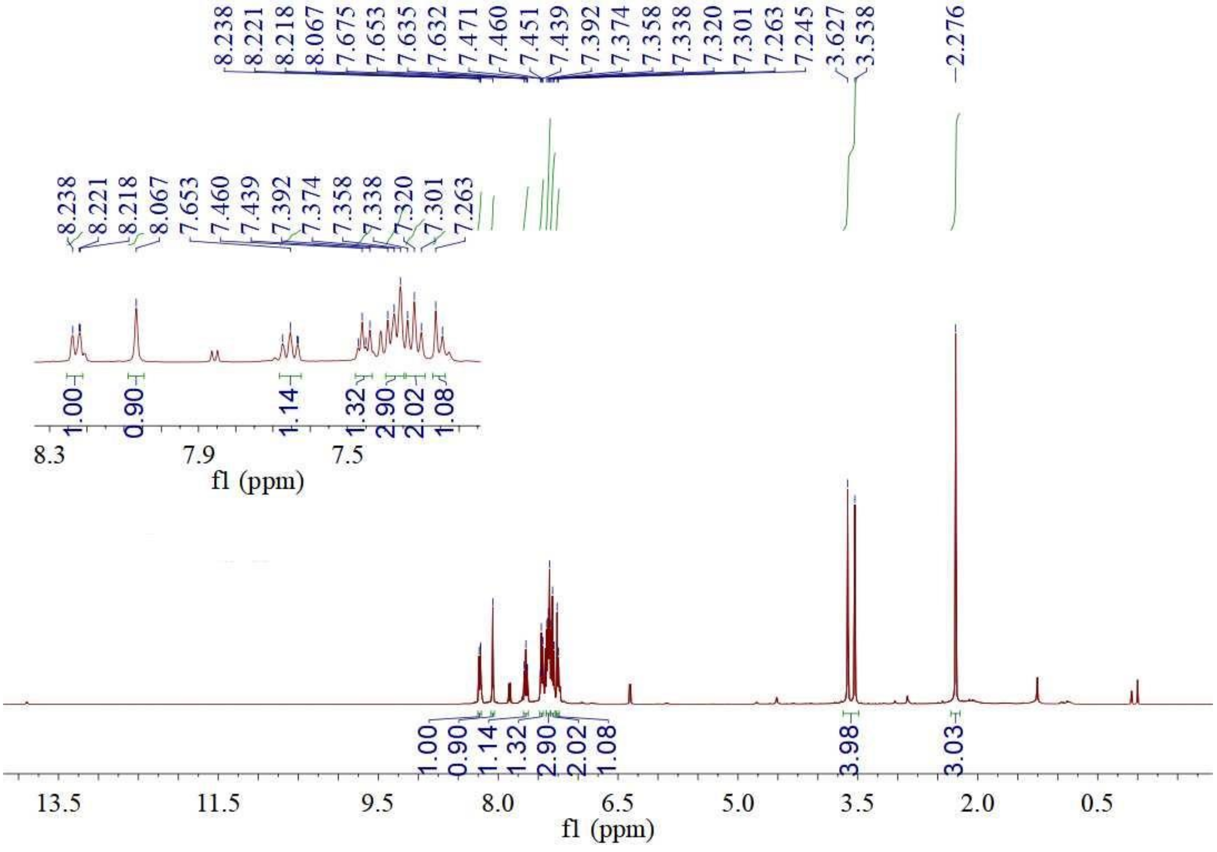

^{13}C NMR spectrum of compound 4z

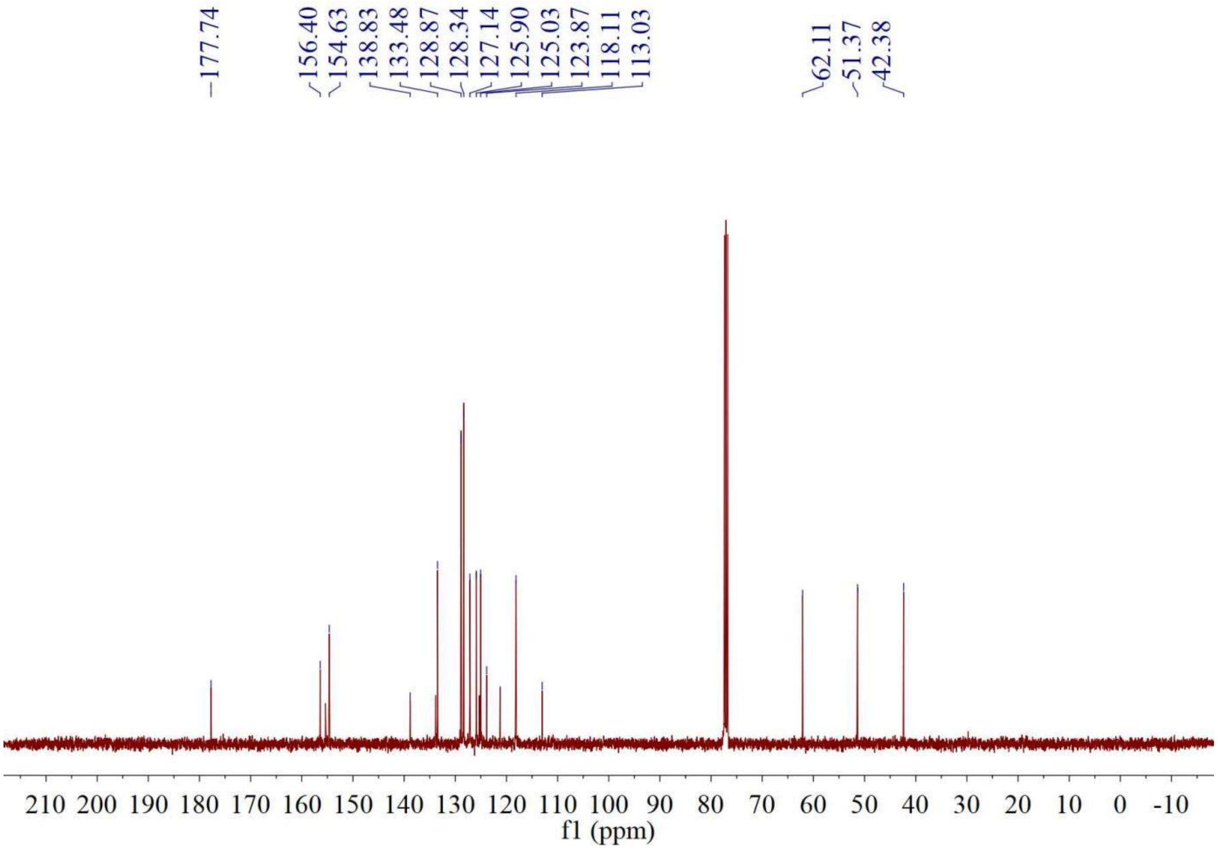

^{1}H NMR spectrum of compound 5

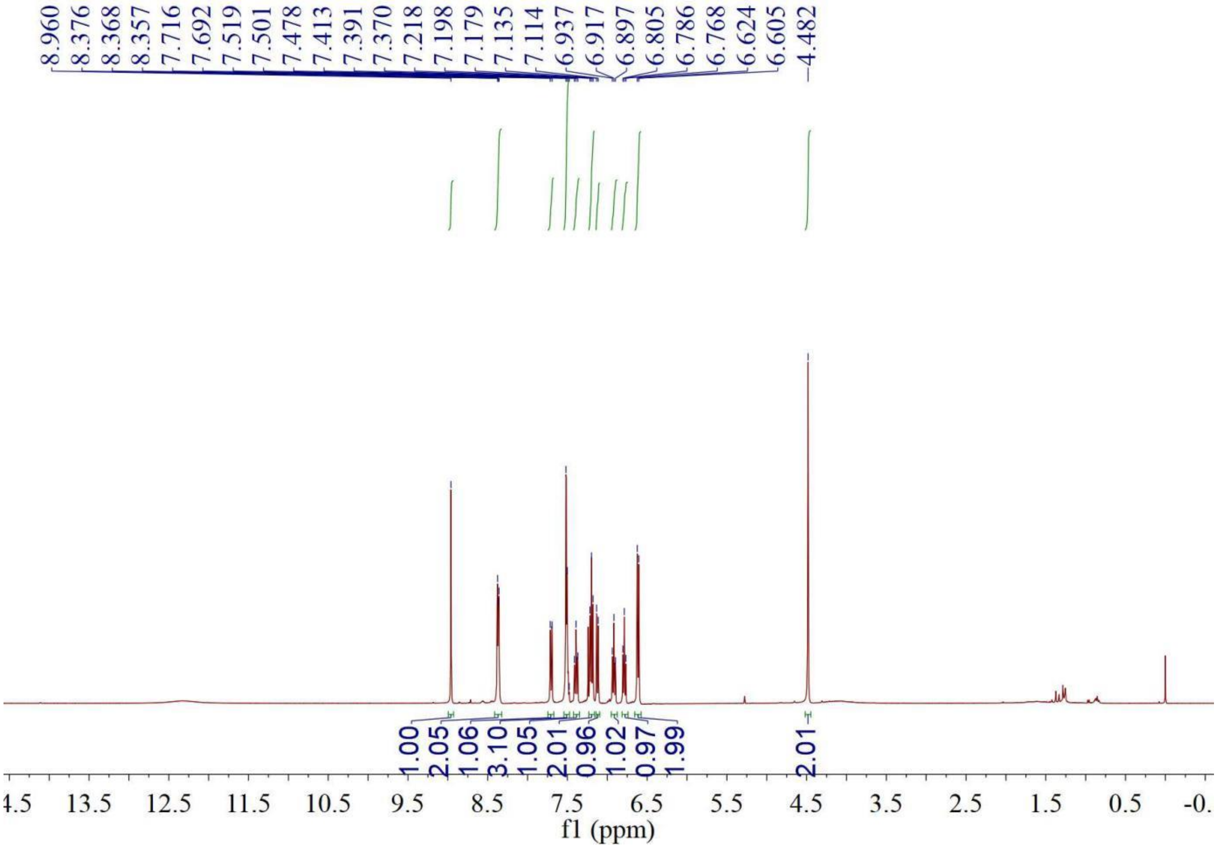

^{13}C NMR spectrum of compound 5

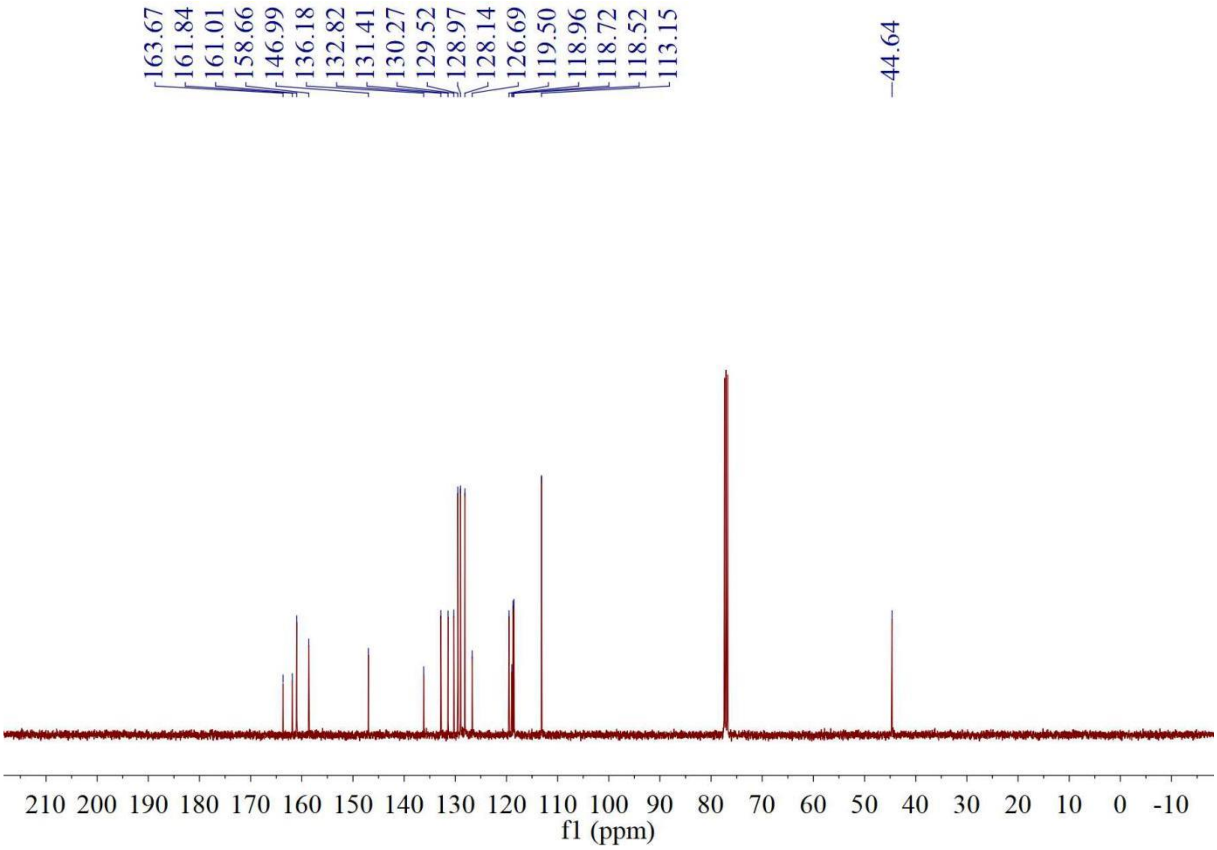

^{1}H NMR spectrum of compound 6

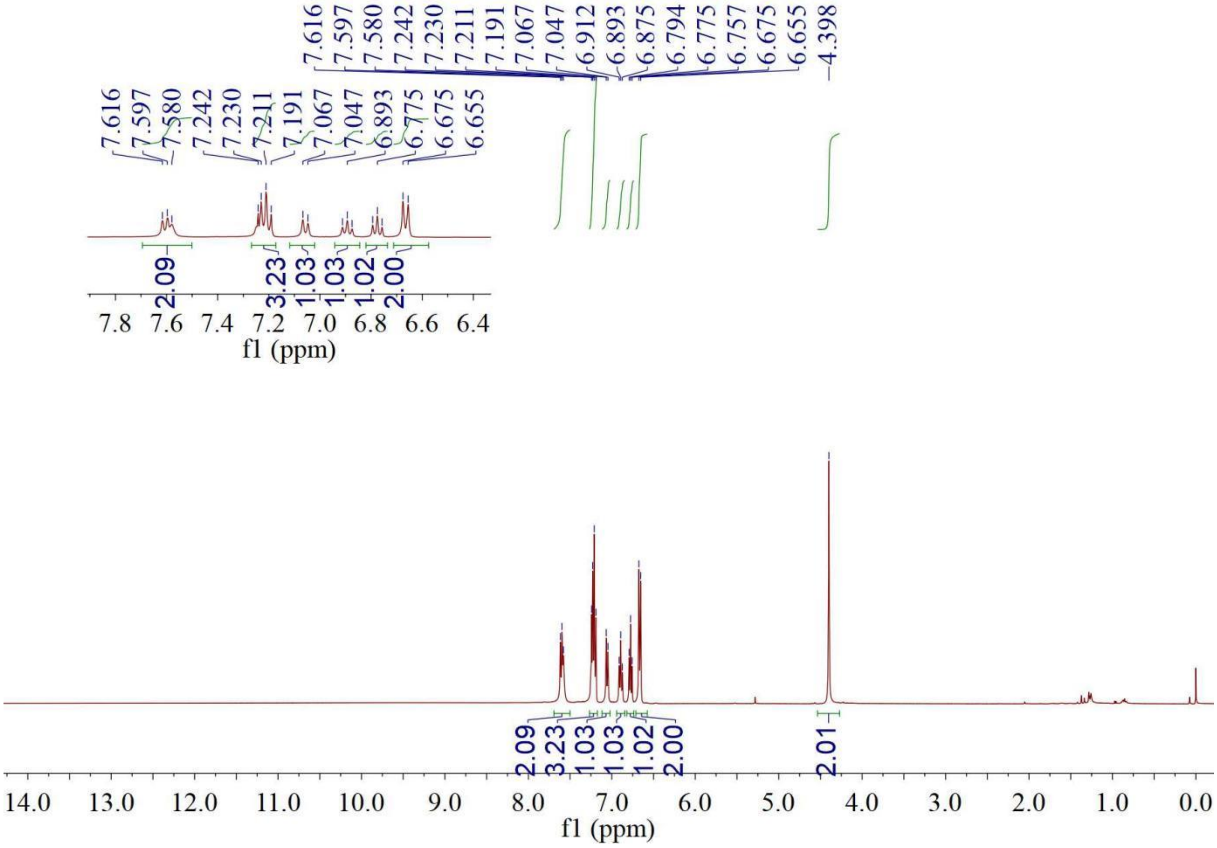

^{13}C NMR spectrum of compound 6

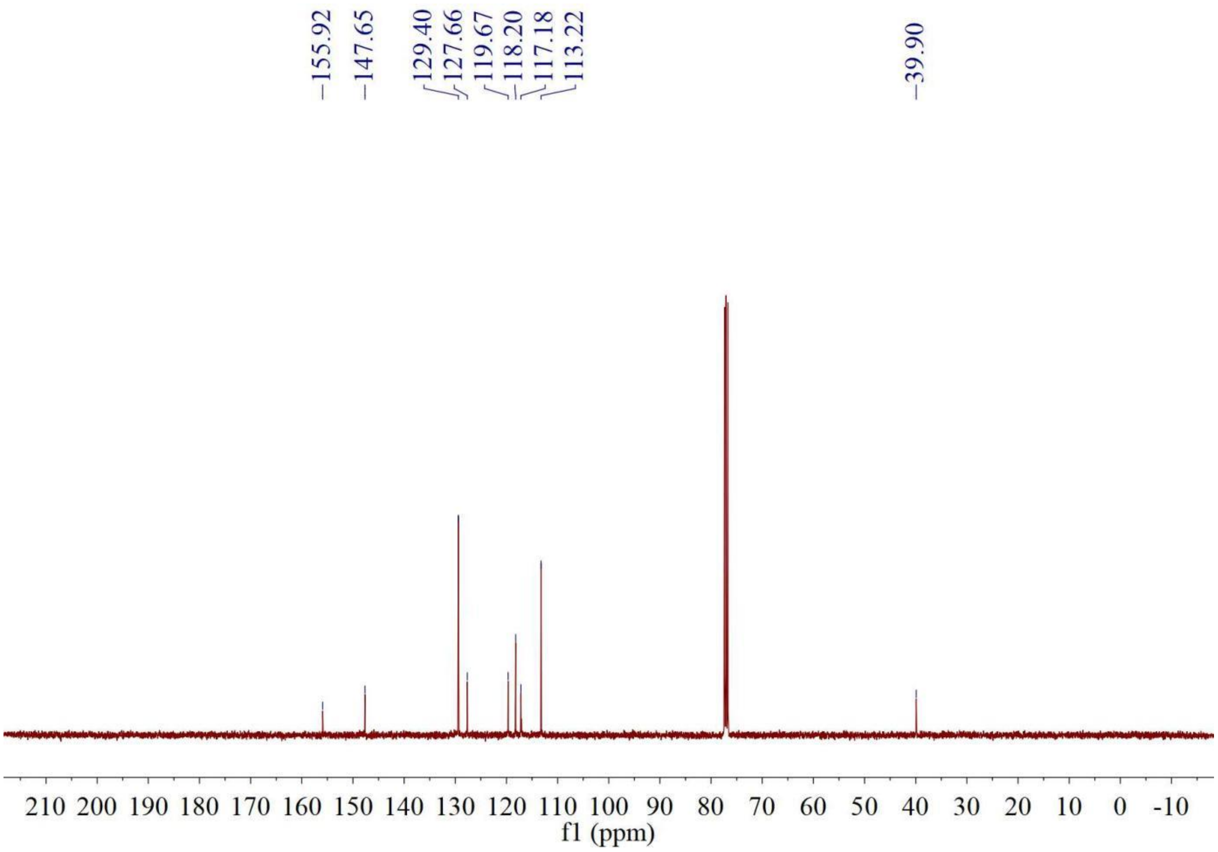

Supplement: Supplementary file 1 [file molecules-31-01432-s001.zip › molecules-4255280-supplementary.pdf]
